# Comparison of Cardiac miRNA Transcriptomes Induced by Diabetes and Rapamycin Treatment and Identification of a Rapamycin-Associated Cardiac MicroRNA Signature

**DOI:** 10.1155/2018/8364608

**Published:** 2018-12-17

**Authors:** Anthony M. Belenchia, Madhavi P. Gavini, Ryan G. Toedebusch, Vincent G. DeMarco, Lakshmi Pulakat

**Affiliations:** ^1^Dalton Cardiovascular Research Center, University of Missouri, Columbia, MO, USA; ^2^Department of Nutrition and Exercise Physiology, University of Missouri, Columbia, MO, USA; ^3^Novopyxis, Boston, MA, USA; ^4^Department of Medicine, University of Missouri, Columbia, MO, USA; ^5^Department of Medical Pharmacology and Physiology, School of Medicine, University of Missouri, Columbia, MO, USA; ^6^Harry S. Truman Memorial Veterans Hospital, University of Missouri, Columbia, MO, USA

## Abstract

Rapamycin (Rap), an inhibitor of mTORC1, reduces obesity and improves lifespan in mice. However, hyperglycemia and lipid disorders are adverse side effects in patients receiving Rap treatment. We previously reported that diabetes induces pansuppression of cardiac cytokines in Zucker obese rats (ZO-C). Rap treatment (750 *μ*g/kg/day for 12 weeks) reduced their obesity and cardiac fibrosis significantly; however, it increased their hyperglycemia and did not improve their cardiac diastolic parameters. Moreover, Rap treatment of healthy Zucker lean rats (ZL-C) induced cardiac fibrosis. Rap-induced changes in ZL-C's cardiac cytokine profile shared similarities with that of diabetes-induced ZO-C. Therefore, we hypothesized that the cardiac microRNA transcriptome induced by diabetes and Rap treatment could share similarities. Here, we compared the cardiac miRNA transcriptome of ZL-C to ZO-C, Rap-treated ZL (ZL-Rap), and ZO (ZO-Rap). We report that 80% of diabetes-induced miRNA transcriptome (40 differentially expressed miRNAs by minimum 1.5-fold in ZO-C versus ZL-C; *p* ≤ 0.05) is similar to 47% of Rap-induced miRNA transcriptome in ZL (68 differentially expressed miRNAs by minimum 1.5-fold in ZL-Rap versus ZL-C; *p* ≤ 0.05). This remarkable similarity between diabetes-induced and Rap-induced cardiac microRNA transcriptome underscores the role of miRNAs in Rap-induced insulin resistance. We also show that Rap treatment altered the expression of the same 17 miRNAs in ZL and ZO hearts indicating that these 17 miRNAs comprise a unique Rap-induced cardiac miRNA signature. Interestingly, only four miRNAs were significantly differentially expressed between ZO-C and ZO-Rap, indicating that, unlike the nondiabetic heart, Rap did not substantially change the miRNA transcriptome in the diabetic heart. *In silico* analyses showed that (a) mRNA-miRNA interactions exist between differentially expressed cardiac cytokines and miRNAs, (b) human orthologs of rat miRNAs that are strongly correlated with cardiac fibrosis may modulate profibrotic TGF-*β* signaling, and (c) changes in miRNA transcriptome caused by diabetes or Rap treatment include cardioprotective miRNAs indicating a concurrent activation of an adaptive mechanism to protect the heart in conditions that exacerbate diabetes.

## 1. Introduction

Obesity and diabetes are metabolic diseases that increase risks for cardiovascular, immune, and inflammatory disease. Chronic inflammation in patients with obesity and diabetes is characterized by an impaired immune response and increased risk of infections [1, 2]. We recently reported that diabetic Zucker obese (ZO) rats exhibit an intracardiac cytokine protein expression profile that reflects deficient host defense compared to that of age-matched healthy Zucker lean (ZL) rats [3]. Moreover, we observed that host defense deficiency entailed suppression of both proinflammatory and anti-inflammatory cytokines. We also reported that ZL and diabetic ZO rats exhibited differential metabolic, cardiac structural, functional, and immune responses to rapamycin, an immunosuppressive agent that inhibits the mechanistic target of rapamycin complex 1 (mTORC1) [3].

Rapamycin (Rap) is a macrolide antibiotic and is used as an effective immunosuppressant during solid organ transplantation [4–6]. It is used as an anticancer drug because mTORC1 signaling is hyperactivated in up to 70% of human cancers [7–12]. mTORC1 inhibition has been proposed as an effective strategy for stabilization of atherosclerotic plaques [13]. The mTOR signaling network is implicated in cellular senescence, aging, and lifespan regulation, and rapamycin treatment improves lifespan in different model organisms [14–16]. In brief, Rap and rapalogues (new inhibitors of mTORC1) exert several beneficial effects in the treatment of chronic diseases. On the other hand, accumulating evidence from clinical trials indicates that adverse metabolic side effects of Rap treatment include new onset diabetes and lipid disorders [17–19]. While Rap reduced mortality in healthy mice, paradoxically, long-term rapamycin treatment increased mortality in diabetic mice [20]. We recently reported the suppression of intracardiac expression of GM-CSF, IL-2, IFN-*γ*, and IL-10, as well as increased decorin and prolactin in diabetic rats and rapamycin-treated nondiabetic rats, indicating a similarity in cardiac cytokine signaling associated with both diabetes and Rap treatment [3]. These observations underscore the need for a better understanding of the molecular regulators that mediate the effects of rapamycin in the diabetic heart.

We have shown previously that when diabetic ZO rats were treated for 12 weeks with a low dose of Rap (750 *μ*g/kg/day delivered via subcutaneous injection), they exhibited significant increase in their fasting glucose levels [3]. While Rap treatment suppressed cardiac fibrosis in ZO rats, it induced cardiac fibrosis in heathy ZL rats, suggesting that mTORC1 inhibition exerts differential effects in diabetic versus healthy animals [3]. Recent studies have identified several microRNAs that mediate the effects of Rap treatment in different cancers [21–26]. MicroRNAs are short (~23 nt) noncoding RNA molecules that function as master regulators of networks of gene expression by virtue of their ability to bind hundreds or thousands of mRNAs [27]. The human genome has over 2000 microRNAs that are predicted to regulate one-third of the genes in the genome [28]. To date, there are no reports that describe how Rap treatment modulates the microRNA expression profiles in healthy and diabetic hearts.

Given the similarities between the intracardiac cytokine expression patterns of Rap-treated and diabetic hearts and that induction of diabetes is one of the main adverse effects of Rap treatment, we hypothesized that there could be significant similarities in the cardiac microRNA transcriptome induced by diabetes and Rap treatment. We further hypothesized that Rap treatment may induce a shift towards increased expression of miRNAs implicated in fibrosis in healthy rat hearts. This hypothesis was tested in the present study using the same four rat models (and corresponding individual rats within those four groups) that we used to characterize how diabetes and Rap treatment modulate intracardiac cytokines. ZL rats (ZL-C) served as baseline controls for miRNA expression in the healthy heart. ZO rats (ZO-C) served as the controls for obesity- and diabetes-induced changes in cardiac miRNA expression. Parallel groups of ZL and ZO rats were treated with Rap for 12 weeks to evaluate the effects of Rap treatment on cardiac miRNA expression in healthy and diabetic hearts, respectively.

We used the GeneChip miRNA 4.0 Array (Thermo Fisher Scientific) to profile miRNA expression in placebo or Rap-treated ZL and ZO rat hearts. Here, we report that changes in intracardiac miRNA transcriptomes induced by rapamycin treatment and diabetes shared significant similarities and provide new insights into mechanisms underlying adverse effects of rapamycin. Differentially expressed miRNAs showed significant correlation with cardiac fibrosis in Rap-treated healthy ZL and diabetic ZO rats. This analysis also uncovered a new, Rap-induced cardiac microRNA signature. Additionally, our *in silico* analysis indicated that human orthologs of rat miRNAs that were highly correlated with cardiac fibrosis in the rats used in this study are involved in modulating the profibrotic TGF-*β* pathway.

## 2. Methods

### 2.1. Rapamycin Treatment of Rats

Rap treatment of 8-week-old ZL and ZO rats was performed as described previously [3]. All animal procedures used in this study were approved by the Harry S. Truman Memorial Veterans Hospital (HSTMVH) Subcommittee for Animal Safety and University of Missouri IACUC before commencing. All animals were cared for in accordance with the guidelines for the care and use of laboratory animals (National Institutes of Health publication 85-23). Briefly, 8-week-old ZO (fa/fa) and lean (ZL) rats (Charles River Laboratories) were maintained on ad libitum food and water and housed singly at the HSTMVH animal housing facility under standard laboratory conditions at room temperature 21–22°C. Animals were entrained to have dark cycle (12 hr: awake time) during the day and light cycle (12 hr: sleep time) during the night so that all interactions with animals matched their awake time. Placebo pellets or rapamycin pellets designed to deliver Rap at a concentration of 750 *μ*g/kg/day for 21 days (from Innovative Research of America Inc., Sarasota, FL) were surgically placed under the skin behind the shoulder blades under brief isoflurane anesthesia, and this procedure was repeated 3 times to achieve a 12-week treatment. ZL and ZO rats that received placebo pellets are referred as ZL-C and ZO-, and those received Rap pellets are referred as ZL-Rap and ZO-Rap, respectively.

### 2.2. Cardiac miRNA Isolation, Microarray Analysis, and Quantitative Real-Time PCR

Frozen heart tissue from saline- and Rap-treated ZL and ZO rats stored at −80°C was powdered under liquid nitrogen, and miRNA isolation was performed using mirVana miRNA isolation kit (Ambion) following the manufacturer's protocol and quantified using NanoDrop (Thermo Scientific) as described previously [3]. FlashTag™ Biotin HS RNA Labeling Kit for GeneChip® miRNA Array was used for generating miRNA probes as per manufacturer's instructions using 300 ng of miRNA as input per reaction. Hybridization and scanning of the arrays were performed at the Microarray Core Lab at the University of Colorado, Denver, for a fee. GeneChip miRNA 4.0 Arrays are designed to interrogate all mature miRNA sequences in miRBase release 20. CEL files generated from the scanning of arrays were analyzed using the miRNA microarray data QC analysis as described in the Affymetrix Expression Console Software 1.4 user manual for data normalization. The robust multichip analysis (RMA) + DBAG workflow (Rat), that performs quantile normalization and has a general background correction, was used to generate CHP files. Threshold test showed that all CHIP files were within bounds. Significance of differentially expressed miRNAs between different pairs (ZL-C versus ZO-C, ZL-C versus ZL-Rap, and ZL-C versus ZO-Rap, as well as ZO-C versus ZO-Rap) was determined using unpaired two-tailed *t*-test. cDNA was generated from the previously isolated miRNA using the miScript II RT Kit (Qiagen, Valencia, CA). Real-time PCR reactions were performed in triplicate using miScript II SYBR Green PCR Kit and prevalidated Qiagen miScript Primer Assays for miR-21-5p (cat. #MS00013216), miR-144-3p (cat. #MS00021833), miR-155-5p (cat. #MS0001701), miR-101b-3p (cat. #MS00012964), miR-26b-3p (cat. #MS00000140), miR-30e-3p (cat. #MS00013426), and miR-34b-3p (cat. #MS0027468). Reactions were performed using the Bio-Rad IQ5 (Bio-Rad, Hercules, CA) under cycle conditions specified by the manufacturer. The expression levels of target miRNAs relative to endogenous control (RNU6-2; cat. #MS00033740, Qiagen) were quantified by a comparative quantitation cycle method. Relative quantification (RQ) values were obtained by determining ΔCt values followed by determining ΔΔCt values and then RQ values via the equation 2(−ΔΔCt).

### 2.3. Principal Component Analysis (PCA)

RMA + DBAG workflow identified 1218 rat miRNAs. Of the 1218 miRNAs, one-way ANOVA (SAS 9.4, PROC ANOVA) revealed 70 miRNAs that were differentially expressed (*p* < 0.05) by at least 1.5 log2-fold between one or more groups. To identify similarities in miRNA expression patterns between the groups, PCA was performed on these 70 miRNAs using SAS 9.4 software, PROC PRINCOMP.

### 2.4. *In Silico* Analysis for Identifying Differentially Expressed Cytokines Targeted by Differentially Expressed miRNAs

NCBI gene database was used to retrieve complete mRNA sequence data for differentially expressed cytokines identified from pairwise comparison of ZO-C or ZO-Rap versus ZL-C that we reported previously [3]. RegRNA software [29] was used to retrieve predicted miRNA binding sites for each of the cytokines. miRNA binding sites for each cytokine mRNA in the differentially expressed cytokine list for a given pairwise comparison (e.g., ZO-C versus ZL-C) were compared with the list of differentially expressed miRNAs in the same pairwise comparison. Then, the data were compiled, and the list of different cytokines targeted by a given miRNA was organized and presented in Table 1.

### 2.5. *In Silico* Analysis to Determine Correlation of Fibrosis with Differentially Expressed miRNAs

Human orthologs for miRNAs demonstrating a significant relationship (*p* < 0.05) with cardiac fibrosis were entered into DIANA-miRPath v3.0 software [30] to determine associated pathways in humans, using both Kyoto Encyclopedia of Genes and Genomes (KEGG) and Gene Ontology-Biological Processes (GO-bp) analyses, and target genes associated with those pathways. Secondary KEGG pathway enrichment analyses and subsequent identification of target genes along significantly enriched pathways were performed using miRNet (http://www.miRNet.ca/).

### 2.6. Statistical Analysis

Results are reported as means ± SE. Statistical analysis was performed using SigmaStat or SAS 9.4 software. Unpaired two-tailed *t*-test was performed for pairwise comparisons. A *p* value < 0.05 was deemed significant. Spearman correlation coefficients were obtained for miRNA expression and measures of cardiac fibrosis, independent of the treatment group, using PROC REG (SAS 9.4).

## 3. Results

**Figure 1:**
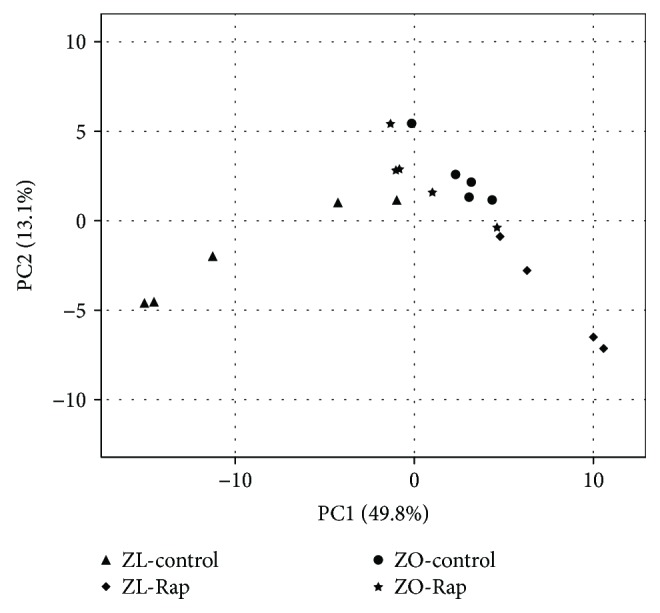
PCA analysis of the 70 miRNAs determined to be differentially expressed (*p* < 0.05) by at least 1.5 log2-fold between one or more groups. Comparisons revealed three moderately distinct clusters (1) ZL-control (ZL-C), (2) ZL-Rap, and (3) ZO-control (ZO-C) and ZO-Rap.

One-way ANOVA of the 1218 rat miRNAs used as probes in this study showed that 70 miRNAs exhibited statistically significant (*p* < 0.05) differential expression by at least 1.5 log2-fold between one or more groups (Figure 1). Principal component analysis (PCA) of these 70 miRNAs showed that ZL-C and ZL-Rap were distinct groups whereas ZO-C and ZO-Rap groups clustered together. This observation suggested that while Rap treatment had strong effects on the miRNA transcriptome of ZL rats, Rap did not alter the miRNA transcriptome of ZO-rats to the same extent.

### 3.1. Comparison of Cardiac miRNA Transcriptome in ZL-C and ZO-C

**Figure 2:**
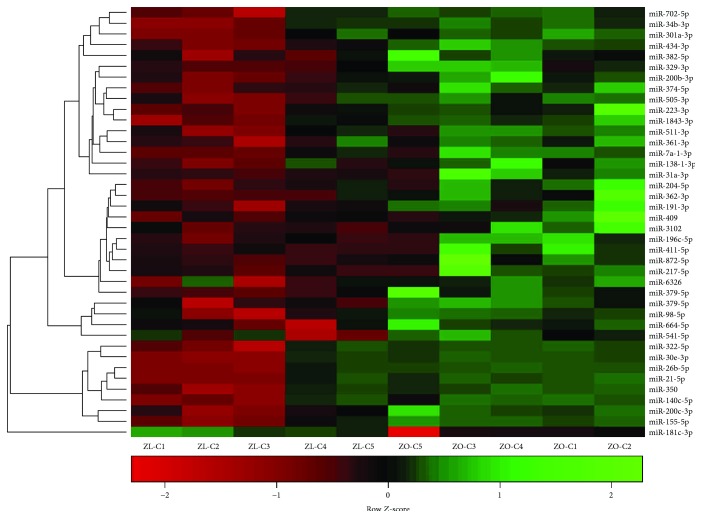
Hierarchal cluster heat map of differentially expressed cardiac miRNAs in ZL-C (*n* = 5) vs. ZL-Rap (*n* = 4) that exhibited a 2.25-fold change in expression in either direction. Red signals indicate lower expression levels, and green signals indicate higher expression levels.

We previously reported metabolic and cardiac parameters of ZL-C and ZO-C used in this study and differences in their intracardiac cytokine profiles [3]. In this study, miRNA transcriptomes of the same heart tissues were analyzed. Diabetes induced significant differential expression of a total of 177 cardiac microRNAs in ZO-C (Supplemental Table 1). Specifically, 105 miRNAs showed an increase, and 72 miRNAs showed a reduction in their expression in ZO-C compared to ZL-C. Among them, 40 microRNAs showed differential expression ≥ 1.5 log2-fold (Figure 2). A literature search provided evidence that 20 of these miRNAs are associated with diabetes and/or cardiac fibrosis and/or different cardiovascular diseases (Table 2). The microRNA miR-155-5p, a biomarker that is increased in the plasma of patients with type 1 diabetes [31, 32] and in gingival crevicular fluid of patients with periodontitis and type 2 diabetes [33] and contributes to cardiac hypertrophy and coronary heart disease [34, 35], was increased by 5.21-fold in the ZO rat heart, consistent with the cardiovascular detrimental effects of diabetes. Other miRNAs that have defined roles in cardiovascular damage included miR-872-5p, miR-350, miR-362-3p, miR-223-3p, miR-204, miR-98, miR-217, miR-379, and miR-181-3p (Table 2 and references therein). MicroRNAs that contribute to fibrosis such as miR-21, miR-382, miR-155, miR-223-3p, and miR-217 were also increased in ZO-C hearts compared to ZL-C hearts (Table 2). However, miRNAs that suppress fibrosis (miR-200b/c, miR-411-5p, miR-140, miR-322, and miR-98) and render cardiovascular protective effects (miR-21 and miR-140) were also simultaneously increased in the ZO-C heart indicating the activation of an adaptive mechanism to regulate fibrosis induced by high glucose and insulin resistance.

### 3.2. Comparison of Cardiac miRNA Transcriptome in ZL-C and ZL-Rap

**Figure 3:**
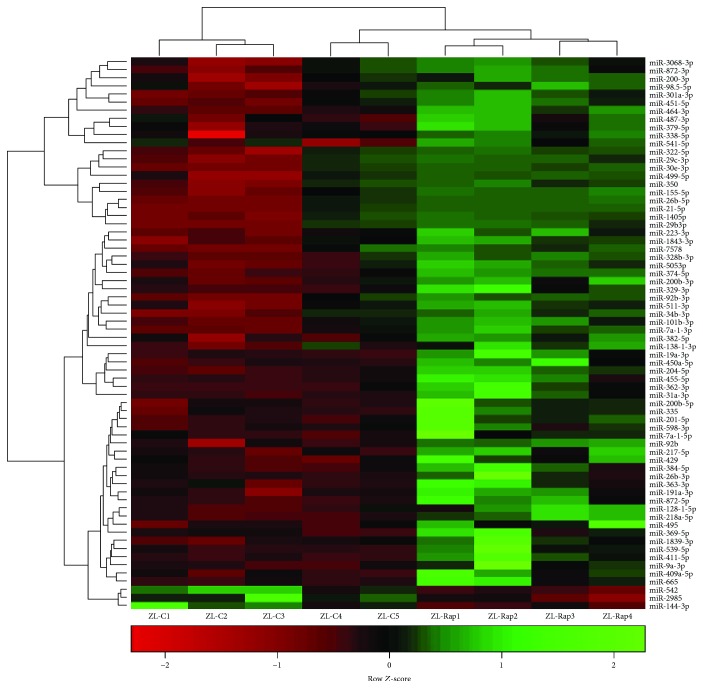
Hierarchal cluster heat map of differentially expressed cardiac miRNAs in ZL-C (*n* = 5) vs. ZO-C (*n* = 5) that exhibited a 2.25-fold change in expression in either direction. Red signals indicate lower expression levels, and green signals indicate higher expression levels.

**Figure 4:**
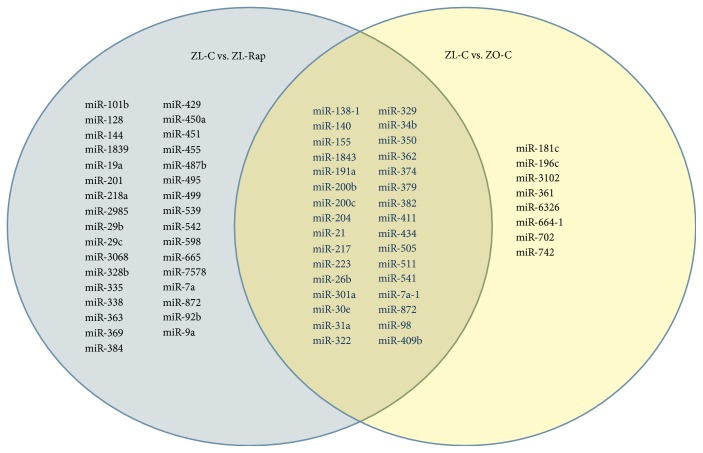
Venn diagram demonstrating miRNA that exhibited a 2.25-fold change in expression in either direction between ZL-C vs. ZL-Rap (left) and ZL-C vs. ZO-C (right), as well as those that were differentially expressed in both comparisons (center).

A total of 221 cardiac miRNAs were differentially expressed between ZL-C and ZL-Rap that showed statistical significance. Out of these, 131 cardiac miRNAs had increased expression and 90 miRNAs had decreased expression in ZL-Rap compared to ZL-C (Supplemental Table 2). Further selection of miRs that exhibited at least a 1.5 log2-fold difference showed that 68 miRNAs were differentially expressed between ZL-C and ZL-Rap (Figure 3). We found that 32 of these miRNAs (47%) were the same and showed the same directionality of expression as those that were found to be differentially expressed between ZL-C and ZO-C (Figure 4). This accounted for 80% of the differentially expressed cardiac miRNAs in ZO-C. Therefore, there is a remarkable similarity between the cardiac transcriptome activated by Rap treatment in healthy hearts and that activated in response to diabetes. Table 2 lists 20 of these miRNAs that showed the same expression pattern in ZL-Rap and ZO-C and that are also implicated in diabetes and/or fibrosis and/or cardiovascular damage.

Table 3 lists those miRNAs that were significantly differentially expressed by at least 1.5 log2-fold in either direction in ZL-Rap compared to ZL-C and show some relationship with diabetes and/or fibrosis and/or cardiovascular damage according to literature. These miRNAs were not differentially expressed by 1.5 log2-fold in ZO-C compared to ZL-C and therefore were unique to the ZL-Rap hearts. Several miRNAs that are diabetes markers and contribute to insulin resistance, pancreatic beta cell death, and progression of diabetes are included in this list. For example, miR-29 family miRNAs are significantly increased in patients with T1DM and T2DM and involved in pancreatic beta cell death and progression of diabetes [36–38]. MicroRNAs miR-19a, miR-499, miR-539, miR-363, miR-495, miR-7a, and miR-429, which are reported to be increased in diabetic patients or animal models and/or contribute to insulin resistance, were increased in ZL-Rap hearts suggesting that Rap treatment induces a diabetes-associated miRNA transcriptome in ZL-Rap hearts. However, miR-455 and miR-451, which are reported to be suppressed in diabetes, were found to be increased in ZL-Rap hearts, whereas miR-144 that is shown to be increased in diabetes was suppressed in ZL-Rap hearts [39–41]. Additionally, a miRNA implicated in promoting diabetic wound healing, miR-335, was also among the miRNAs that had increased transcription in ZL-Rap hearts [42].

Several of these miRNAs are also linked to CVD (Table 3). miRNAs that are associated with exacerbating different CVDs according to literature and increased in ZL-Rap hearts include miR-451, miR29 family, miR-19a, miR-499, miR-539, miR-363, miR-495, and miR-429 (Table 3 and references therein). Additionally, loss of miR-144 and miR-542 also exacerbated CVD. Conversely, miR-101b and miR-7a, which are cardioprotective (Table 3 and references therein), are also among the cardiac miRNAs that are increased by Rap treatment.

### 3.3. Comparison of Cardiac miRNA Transcriptome in ZO-C and ZO-Rap

**Figure 5:**
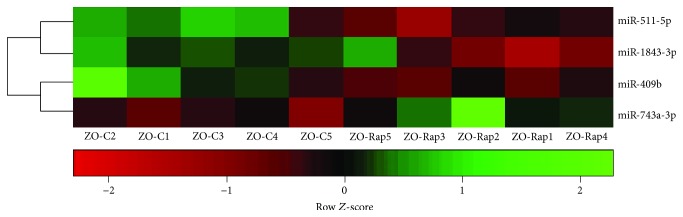
Hierarchal cluster heat map of differentially expressed cardiac miRNAs in ZO-C (*n* = 5) vs. ZO-Rap (*n* = 5) that exhibited a 2.25-fold change in expression in either direction. Red signals indicate lower expression levels, and green signals indicate higher expression levels.

Rap treatment of ZO-C resulted in differential myocardial expression of 128 miRNAs that included 84 miRNAs with increased expression and 44 miRNAs with suppressed expression (Supplemental Table 3). However, only four miRNAs from this group met the ±1.5 log2-fold change threshold (Figure 5). Associations between these miRNAs to specific diseases are shown in Table 4. miR-743a-5p that mediates mitochondrial oxidative stress was increased in the ZO-Rap heart, but not in the ZL-Rap heart, indicating that this is a unique effect caused by the combination of diabetes and Rap treatment (Table 4) [43]. miR-511-3p was the only miRNA that showed similar expression changes in Rap-treated ZL and ZO rats (suppressed in both cases), and loss of miR-511-3p is associated with minimally oxidized low-density lipid-associated increase in atherosclerosis (Table 4) [44]. There is very little information regarding miR-1843-3p and miR-409b in the literature; however, Rap treatment had opposing effects on the expression of these miRNAs in healthy ZL-C and diabetic ZO-C (Table 4).

### 3.4. Comparison of Cardiac miRNA Transcriptome in ZL-C and ZO-Rap

**Figure 6:**
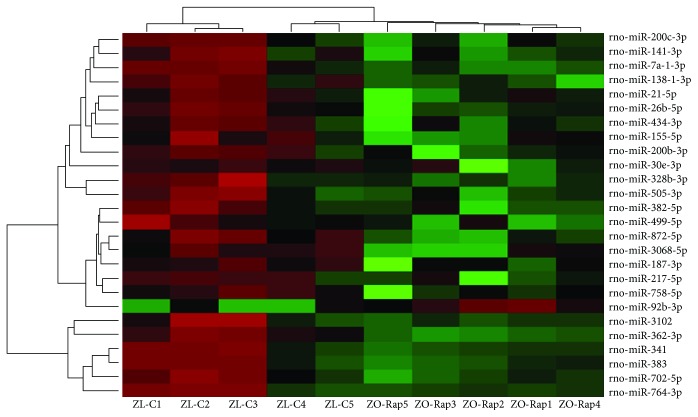
Hierarchal cluster heat map of differentially expressed cardiac miRNAs in ZL-C (*n* = 5) vs. ZO-Rap (*n* = 5) that exhibited a 2.25-fold change in expression in either direction. Red signals indicate lower expression levels, and green signals indicate higher expression levels.

Next, we compared the cardiac miRNA transcriptome of ZO-Rap with that of ZL-C. Of the 116 miRNAs that were differentially expressed between these two groups (Supplemental Table 4), 83 had increased expression and 33 exhibited suppressed expression in ZO-Rap hearts. However, only 27 of these miRNAs met the ±1.5 log2-fold change threshold (Figure 6). Importantly, 17 of these miRNAs were the same as those that were increased in ZL-Rap (Table 1). Among these miRNAs, miR-30e-3p is shown to be suppressed by insulin [45]; however, another study showed that its suppression is associated with myocardial injury induced by coronary microembolization via autophagy activation [46]. Moreover, miR-92b-3p is shown to be suppressed by hypoxia [47], but its expression is elevated in the heart tissues of both Rap-treated groups. One miRNA in this group, miR-7a-1-3p, is not known to associate with diabetes, fibrosis, or CVD. The remaining miRNAs in this group are associated with either diabetes, fibrosis, or cardiovascular diseases as shown in Table 1. Because myocardial expression of these 17 miRNAs is increased by Rap treatment in both ZL and ZO rats, we propose that these miRNAs constitute a Rap-induced cardiac microRNA signature.

### 3.5. Cytokines Targeted by the Differentially Expressed miRNAs

We previously reported which intracardiac cytokines were differentially expressed in response to diabetes (ZL-C versus ZO-C) and Rap treatment (ZL-C versus ZL-Rap) [3]. Because there was significant overlap between the miRNAs that were differentially expressed between these two groups, we hypothesized that these miRNAs would play a role in regulating differentially expressed cytokines in these groups. To determine if the differentially expressed miRNA transcriptome exerts the regulation of the corresponding cytokine profile, we performed an *in silico* analysis using miRbase miRNA target analysis. The miRNAs that had binding sites on the 3′ untranslated sites of the mRNAs expressing differentially expressed cytokines from each pairwise comparison were selected.  Table 5 shows miRNAs that exhibited increased expression and their target cytokines that carried binding sites for the corresponding miRNAs on the 3′ untranslated regions of their mRNAs. As shown in Table 5, differentially expressed miRNAs had binding sites on the 3′ untranslated regions of most of the differentially expressed cytokines. Therefore, changes in expression patterns of intracardiac cytokines in response to diabetes and rapamycin treatments in rat hearts were associated with changes in their miRNA transcriptomes.

### 3.6. Differentially Expressed miRNAs That Correlated with Fibrosis and Validated by Real-Time PCR

**Figure 7:**
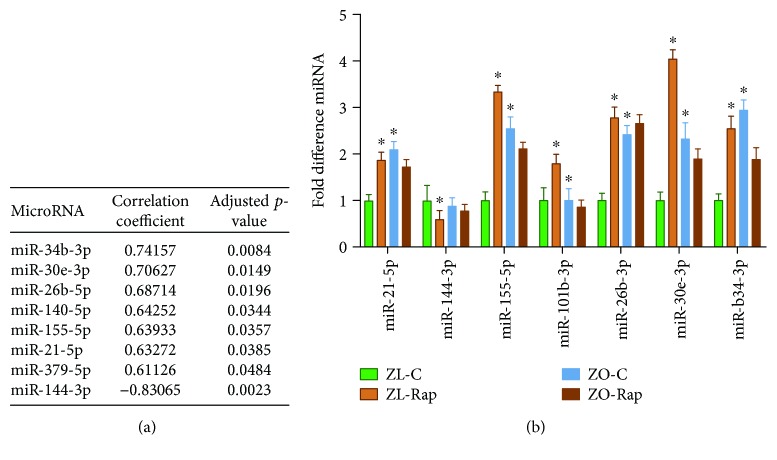
(a) Correlation coefficients and *p* values of fibrotic scores and differentially expressed miRNA from all groups. (b) Comparative miRNA expression levels of several miRNAs that were associated with cardiac fibrosis scores. Data represents means ± SEM. *n* = 4 for all groups. ^∗^ *p* < 0.05 vs. the ZL-C group.

We have previously published that ZL rats treated with rapamycin have increased cardiac fibrosis compared with untreated ZL rats [3]. However, in ZO rats, treatment with rapamycin attenuated cardiac fibrosis [3]. In order to determine which specific miRNA may be contributing to cardiac fibrosis, we performed a correlation analysis using all differentially expressed miRNAs that exhibited at least 1.5 log2-fold change in pairwise comparisons. Analysis of the combined data from all treatment groups showed that seven miRNAs were positively correlated (miR-140-5p, miR-155-5p, miR-21-5p, miR-26b-5p, miR-30e-3p, miR-34b-3p, and miR-379-5p; *p* < 0.05) and one miRNA, miR-144-3p, was inversely correlated (miR-144; *p* < 0.05) with the degree of cardiac fibrosis (Figure 7). Of the seven positively correlated miRNAs, there is evidence supporting strong profibrotic activity for miR-21 and miR-34b [48–52]. In contrast to the positive correlation with fibrosis observed here, miR-140 is considered antifibrotic [53, 54]. miR-155 and miR-34b demonstrate both pro- and antifibrotic activities [55, 56]. miR-140, the only one demonstrating an inverse association, has well-documented antifibrotic activity [57, 58]. To further validate differential expression of some of these miRNAs, we performed quantitative RT-PCR. As shown in Figure 7, miRNAs (miR-34b-3p, miR-26b-3p, miR-140-5p, miR-155-5p, miR-21-5p, and miR-379-5p) exhibited differential expression consistent with the data obtained from microarray analysis.

## 4. Discussion

In this investigation, we have identified an identical subset of the cardiac miRNA transcriptome, comprised of 32 miRNAs, which are differentially expressed in the same direction in the hearts of diabetic ZO rats (ZO-C) and nondiabetic ZL rats treated with Rap (ZL-Rap). Since ZL rats did not develop diabetes after 3 months of Rap treatment [3] and Rap treatment is reported to mitigate aging [14–16], this high similarity between diabetes- and Rap-induced alterations to the cardiac miRNA transcriptome was surprising. Our recent findings that diabetes suppresses both inflammatory and anti-inflammatory intracardiac cytokines may shed some light in this regard [3]. As such, we posit that the similarity in the altered expression of identical miRNAs in the cardiac microRNA transcriptome between diabetic ZO-C and Rap-treated ZL rats might reflect that fact that both diabetes and Rap treatment cause significant immune suppression. Importantly, these studies also identified an identical subset of 17 miRNAs that exhibited increased expression in response to Rap treatment in both ZL and ZO hearts (Table 1). To our knowledge, this is the first evidence of a Rap-induced cardiac microRNA signature common to both healthy and diabetic hearts. It is noteworthy that most of these miRNAs seem to be associated with increasing CVD. This information is clinically relevant and provides new targets for developing drugs that can be coadministered with Rap to reduce potential detrimental effects of long-term Rap treatment in patients with comorbidities.

Previous studies to identify Rap-induced changes in the miRNA transcriptome in cell models of tuberous sclerosis (TSC) and lymphangioleiomyomatosis (LAM) have identified microRNAs 29b, 21, 24, 221, 106a, and 199a as candidate “RapamiRs” [21]. Among these miRNAs, miR-21 and miR-29b were found to be differentially expressed in ZL-Rap. miR-21 is a key miRNA biomarker of diabetes that causes fibrosis and has been characterized as a “mechano-miR” due to its response to arterial stress and hypertension [59–64]. A direct connection between miR-21 and mTOR has also been reported since miR-21 promotes mTORC1-driven tumorigenesis [65]. Additionally, miR-155 has been identified as a potent autophagy inducer that targets the mTOR signaling pathway [66]. miR-29 family miRNAs are involved in pancreatic beta cell death and exacerbate cardiomyocyte loss [36–38, 67]. It was also reported that long-term Rap treatment induced the upregulation of miR-17–92 and related clusters and downregulation of tumor suppressor miRNAs (miR-7a, miR-706, and miR-320) in rapamycin-resistant tumors [68]. Consistent with this, 3-month Rap treatment increased miR-19a, a member of this cluster. However, Rap treatment actually increased miR-7a expression in ZL-C hearts.

Consistent with the pansuppression of cardiac cytokines in the ZO-C heart [3], we observed an increase in miRNAs that target these cytokines in our *in silico* analysis (Table 5). miRNAs that have predicted binding sites on the 3′ untranslated regions of mRNAs coding for the cytokines, CTACK, PDGFAA, CINC2, IL-2, IL-1*α*, TNF-*α*, IL-10, IFN*γ*, prolactin, and decorin were among the miRNAs that were upregulated in ZO-C hearts over 1.5 log2-fold. It is noteworthy that Notch2, decorin, and prolactin were also suppressed in ZO-Rap hearts [3]. However, in ZL-Rap hearts, while IL-2, GM-CSF, IL-10, and IFN*γ* were suppressed, decorin, Notch2, Gas1, prolactin, Tim1, IL-22, and TWEAK-R were upregulated by Rap treatment. Interestingly, analysis of predicted miRNA binding sites on the 3′ untranslated regions of these mRNAs uncovered binding sites for many of the cardiac miRNAs that exhibited increased expression in ZL-Rap hearts. mRNAs encoding decorin, Notch2, Gas1, prolactin, Tim1, and IL-22 (but not TWEAK-R) carried binding sites for multiple miRNAs that were upregulated by Rap treatment. Although additional experiments are warranted to verify the validity of these miRNA binding sites, collectively, these observations suggest that the presence of a tightly regulated posttranscriptional gene expression pattern is present in the ZL-Rap heart for these cardiac cytokines.

We reported that in the ZO-Rap group, eight intracardiac cytokines were differentially expressed compared to ZO-C. However, only four miRNAs met the criteria of 1.5 log2-fold change in ZO-Rap compared to ZO-C. These miRNAs did not seem to have any predicted binding sites on the eight differentially expressed cytokines in the ZO-C heart. Importantly, cardiac miRNA transcriptome of ZO-C was already 80% similar to that of ZL-Rap and that may be why Rap treatment did not result in any additional major changes in their cardiac miRNA transcriptome that met the criteria of 1.5 log2-fold change.

### 4.1. Correlation of miRNA Transcriptome with Fibrosis

In humans and animals alike, myocardial fibrosis is associated with nearly all forms of cardiovascular disease. Myocardial fibrosis is a condition of multiple etiologies, characterized by the transformation of cardiac fibroblasts to a myofibroblast phenotype. The cardiac remodeling that takes place during this phenotypic change is attributable to several pathologies including but not limited to LV dilation, ventricular stiffening, and cardiomyocyte death; all of which play pivotal roles in the progression to heart failure. We reported that ZL rats treated with rapamycin had greater degrees of cardiac fibrosis than untreated ZL rats. However, in ZO rats, treatment with rapamycin attenuated cardiac fibrosis [3]. Data presented here indicates that changes in the miRNA transcriptome induced by diabetes and Rap treatment shared significant similarities. Interestingly, while some of the miRNAs that were differentially expressed in ZO-C and ZL-Rap compared to ZL-C were profibrotic or increased in conditions of fibrosis, others were involved in suppressing fibrosis (Tables 2 and 3). This observation suggests that while cardiac fibrosis develops in response to diabetes (ZO-C) or Rap treatment of healthy animals (ZL-Rap), a concurrent adaptive mechanism to regulate fibrosis via modulating miRNA transcriptome is also activated. Several signaling pathways have been implicated in the transformation of fibroblasts to myofibroblasts (fibrotic remodeling). Among the best understood of these signaling pathways are transforming growth factor-*β*, which is thought to be the primary regulatory pathway of pathological fibrosis [69, 70].

**Figure 8:**
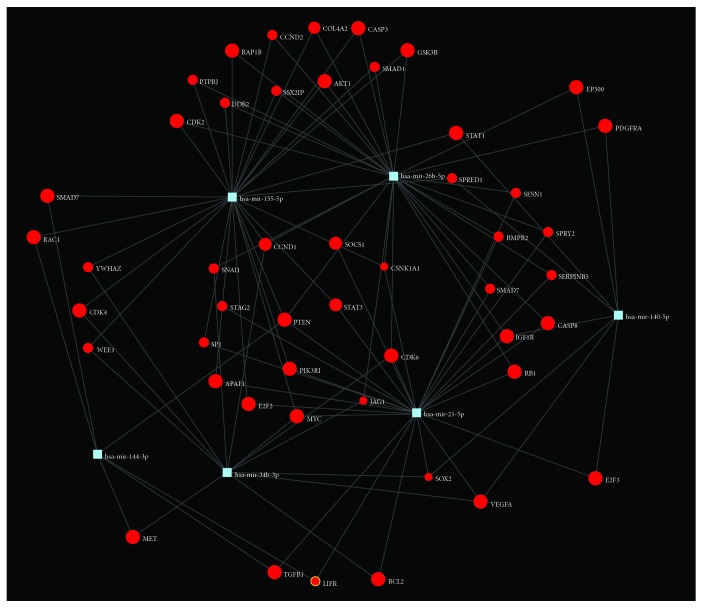
KEGG pathway enrichment analyses and subsequent identification of target genes along significantly enriched pathways associated with cardiac fibrosis. Generated using miRNet software.

While some interspecies variation exists in the expression and target specificity of miRNA, previous studies have demonstrated high degrees of similarity [71, 72]. With the goal of exploring how our differentially expressed miRNA panel may be associated with cardiac fibrosis in humans, we utilized the DIANA software [30] and human orthologs for the miRNA that were significantly correlated with cardiac fibrosis in our rats were used as input. KEGG analysis returned multiple interactions between our miRNA set and genes involved in the TGF-*β* signaling pathway (Table 6; Figure 8). Profibrotic TGF-*β* signaling involves numerous cell surface receptors in addition to TGF-*β* receptors, including those of the bone morphological protein and activin subfamilies (BMPRs and ACVRs, respectively). SMAD proteins are the primary signal transducers of these receptors and therefore are profibrotic with the exception of SMAD7 which inhibits the action of other SMADs. As with most biological pathways, the TGF-*β* pathway contains several internal feedback loops. Collectively, these data suggest that human orthologs of rat miRNAs that showed the highest correlation with cardiac fibrosis are involved in modulating the profibrotic TGF-*β* pathway.

In summary, the data presented here show that 47% of miRNA transcriptome activated in the hearts of healthy rats in response to Rap treatment are identical to 80% of the miRNA transcriptome activated in diabetic rat hearts. In diabetic rat hearts, the miRNA transcriptome could have played a significant role in inducing the pansuppression of anti-inflammatory and proinflammatory intracardiac cytokines. However, while several miRNAs had predicted profibrotic effects, others had antifibrotic effects, suggesting that the miRNA transcriptome may serve as an adaptive mechanism to regulate the progression of cardiac fibrosis. Moreover, human orthologs of rat cardiac miRNAs that exhibited the highest correlation with cardiac fibrosis have the potential to modulate the profibrotic TGF-*β* pathway.

## Figures and Tables

**Table 1:** MicroRNAs differentially expressed in ZO-Rap versus ZL-C and their similarity to microRNAs that were differentially expressed in ZL-Rap versus ZL-C.

MicroRNAs	ZO-Rap/ZL-C	ZL-Rap/ZL-C	Link to diabetes, fibrosis, and/or CVD		
			Diabetes	Fibrosis	CVD
miR-200c/b	4.71/4.099	2.98/4.99	Increased [73, 74]	Suppressed [75]	Increased in familial hypercholesterolaemia [76]
miR-7a-1-3p	4.599	6.844	—	—	—
miR-138-1-3p	4.580	4.238	—		Suppresses cardiac hypertrophy [67, 77]
miR-21	4.533	5.305	Increased [59]	Increased [60]	Reduced in hypertension; cardioprotective [61–63]
miR-26b	4.428	5.147	—	—	Increased in hypertensive patients [78]
miR-434	4.345	9.322	Induces insulin resistance [79]		Increases atrial fibrillation, cardiac damage, heart disease [79–82]
miR-155	4.125	4.55	Increased in T1DM & T2DM [31, 32]	Increased [35]	Increased in cardiac hypertrophy and CHD [33, 34]
miR-30e-3p	4.06	4.80	Suppressed by insulin [45]	—	Suppression associates with cardiac injury [46]
miR-328b-3p	3.734	4.807	—	—	—
miR-505-3p	3.652	4.938	—	—	Increased in familial hypercholesterolaemia [83]
miR-382	3.407	4.56	Increased [84]	Increased [85]	—
miR-499	3.338	3.86	Induces insulin resistance [79]		Increases atrial fibrillation, cardiac damage, heart disease [79–82]
miR-872-5p	3.255	5.519	—	—	Associated with cardiac oxidative stress and atherosclerosis [86, 87]
miR-217	2.996	2.3409	Increased [88]	Increased [89]	Increased in cardiovascular aging [90]
miR-92b-3p	2.942	3.128	—	—	Suppressed in response to hypoxia [47]
miR-362-3p	2.907	6.316	Suppressed in DM patients [91]	Increased in cardiac fibroblasts [92]	Increased in acute myocardial infarction [93]

Please see Supplemental Table 4 for the exact gene expression levels and *p* values.

**Table 2:** Similarities between differentially expressed microRNAs that modulate diabetes, cardiac fibrosis, and other cardiovascular diseases in ZO-C versus ZL-C and ZL-Rap versus ZL-C.

MicroRNA	ZO-C/ZL-C	ZL Rap/ZL-C	Nature of association with human or animal model pathology		
			Diabetes	Fibrosis	CVD
miR-155	5.21	4.55	Increased in T1DM & T2DM [31, 32]	Increased [35]	Increased in cardiac hypertrophy and CHD [33, 34]
miR-200b/c	5.17/4.00	4.99/2.98	Increased [73, 74]	Suppressed [75]	Increased in familial hypercholesterolaemia [76]
miR-21	4.59	5.28	Increased [59]	Increased [60]	Reduced in hypertension; cardioprotective [61–63]
miR-26b-5p	4.41	5.13	—	—	Increased in hypertensive patients [78]
miR-872-5p	4.32	5.52	—	—	Associated with cardiac oxidative stress and atherosclerosis [86, 87]
miR-411-5p	3.89	7.16	—	Suppresses fibrosis [94]	Increased in abdominal aortic aneurism [95]
miR-382	3.84	4.56	Increased [84]	Increased [85]	—
miR-301a	3.81	4.86	Increased [96]		Increased in the diabetic heart [96]
miR-329	3.81	5.50	—	—	Increased in ischemia [97]
miR-350	3.68	3.61	—	—	Induces pathological cardiac hypertrophy [98]
miR-505-3p	3.66	4.92	—	—	Increased in familial hypercholesterolaemia [83]
miR-140	3.58	4.00	Reduced in platelets [99]	Suppresses fibrosis [100]	Suppresses pulmonary arterial hypertension [101]
miR-322	3.56	3.84	Suppressed by high glucose [102]	Suppresses fibrosis [103]	Improved cardiac function [103]
miR-362-3p	3.48	6.28	Increased [104]	—	Associated with atherosclerosis [105]
miR-374	3.34	5.94	—	—	Increased in cardiac hypertrophy and aneurism [106, 107]
miR-223-3p	3.27	3.56	Suppressed in DM patients [91]	Increased in cardiac fibroblasts [92]	Increased in acute myocardial infarction [93]
miR-204	3.20	7.01	Suppresses insulin [108]	—	Increases endoplasmic reticulum stress [109]
miR-98	3.20	3.41	Increased [110]	Reduces collagen [111]	Reduced in myocarditis [112]; increased in postheart transplant [113]
miR-34b	3.10	3.29	—	—	Reduced in diabetic ischemic heart failure [114]
miR-217	2.99	2.98	Increased [88]	Increased [89]	Increased in cardiovascular aging [90]
miR-541	2.97	3.18	Involved in pancreas development [115]	—	Suppresses cardiac hypertrophy [116]
miR-379	2.85	2.91	—	—	Apoptosis of VSMC [117]

Please see Supplemental Tables 1 and 2 for the exact gene expression levels and *p* values.

**Table 3:** MicroRNAs that modulate diabetes, cardiac fibrosis and other cardiovascular diseases and differentially expressed in ZL-Rap versus ZL-C.

MicroRNAs	ZL-Rap/ZL-C	Link to diabetes and/or CVD	
		Diabetes	CVD
miR-455	6.76	Suppressed [39]	—
miR-451	5.1076	Suppressed [40]	Induces cardiac hypertrophy [40]
miR-101b	5.0625	—	Suppresses cardiac hypertrophy [67, 77]
miR-29b/c	4.75/3.83	Increased in diabetes [36–38]	Correlates with CVD progression in diabetes [36–38, 118]
miR-19a	4.7089	Increased in diabetes [119]	Induces heart failure and vascular inflammation [120–122]
miR-499	3.8636	Induces insulin resistance [79]	Increases atrial fibrillation, cardiac damage, heart disease [79–82]
miR-539	3.7249	Increased in diabetes [123]	Induces mitochondrial fission, cardiomyocyte apoptosis [123, 124]
miR-363	3.7249	Urinary marker for diabetes [125]	Inhibition protects cardiomyocytes from apoptosis [126]
miR-335	3.2761	Improves diabetic wound healing [42]	—
miR-92b	3.0976	—	Increased in heart failure patients [127]
miR-495	3.0976	Urinary marker for diabetes [125]	Involved in causing hypertrophy [128, 129]
miR-7a	2.8224	Inhibits glucose-stimulated insulin secretion [130]	Protects against cardiomyocyte injury [131, 132]
miR-429	2.56	Impairs intestinal barrier in diabetic mice [133]	Causes cardiomyocyte apoptosis [134]
miR-487b	2.3409	—	Mitigates chronic heart failure [135]
miR-144	−2.28	Increased in diabetes [41]	Loss of miR-144 impairs cardioprotection [136]
miR-542	−2.28	—	Involved in aortic calcification [137]

Please see Supplemental Table 2 for the exact gene expression levels and *p* values.

**Table 4:** MicroRNAs differentially expressed in ZO-Rap versus ZO-C and their similarity to microRNAs that were differentially expressed in ZL-Rap versus ZL-C.

MicroRNA	ZO-Rap/ZO-C	ZL-Rap/ZL-C	Nature of Association with human or animal model pathology		
			Diabetes	Cardiac fibrosis	CVD
miR-743a-5p	2.25	1.09; *p* = N.S.	—	—	Mediates mitochondrial oxidative stress [43]
miR-1843-3p	−2.25	2	—	—	—
miR-511-3p	−2.25	−1.7	—	—	Suppressed in moX-LDL-induced VSMC transformation in atherosclerosis [44]
miR-409b	−2.25	1.45; *p* = N.S.	—	—	—

N.S.: not significant. Please see Supplemental Table 3 for the exact gene expression levels and *p* values.

**Table 5:** Differentially expressed miRNAs in ZL-C versus ZO-C and ZL-C versus ZL-Rap pairwise comparisons that target differentially expressed cytokines in the corresponding pairwise comparisons and as determined by binding sites on the 3′ untranslated region of their mRNAs by *in silico* analysis in the corresponding sets.

miRNA	Targeted cytokines increased in			miRNA	Targeted cytokines	
	ZO-C	ZL-Rap	Both		ZO-C	ZL-Rap
miR-21	CTACK, PDGFAA, CINC2	Notch2		miR-350		Notch2
miR-411	IL-2, IL-1*α*, TNF-*α* (2 sites)		IL-2, IL-10	miR-505	CINC2	Gas1
miR-7a^∗^	PDGFAA	IL-2, decorin, IL-22	IL-10	miR-217		Notch 2, IL-1*α*
miR-384-5p		IL-2, IL-10, Notch2		miR-541		Notch 2, CINC2
miR-30e^∗^		IL-22, Notch2	IL-10, IFN*γ*	miR-328b-3p		Notch 2, Gas1
miR-322			IFN*γ*	miR-29b		Notch 2
miR-382		Notch2	IFN*γ*, Prolactin	miR-455		Notch2
miR-742			IFN*γ*	miR-34b		Gas1
miR-539		IFN*γ*		miR-19a		Gas1
miR-329^∗^	PDGFAA	Notch2	IL-10	miR-26b-3p		Gas1
miR-140		Notch2	IL-10, decorin	miR-429		Gas1, Tim1
miR-204	TNF-*α*	Gas1	IL-10	miR-128-1-5p		Gas1
miR-98	IL-1*α*		IL-10	miR-144		Gas1
miR-665		IL-10, Notch 2 (3 sites), Tim1		miR-2985		Gas1
miR-335		IL-10, Notch2, Tim 1		miR-301a		Prolactin
miR-495		IL-10 (2 sites), Notch 2, Gas1 (2 sites)		miR-362^∗^		Tim1
miR-338^∗^		IL-10, Notch2		miR-451^∗^		Tim1
miR-200b, c			Decorin	miR-409-5p		Tim1
miR-223			Decorin	miR-218a		Tim1
miR-499		Decorin (2 sites), Gas1		miR-369-5p		Tim1
miR-542-3p		Decorin, Tim1		miR-434		IL-22
miR-138-1^∗^	B7–1/CD80	Notch2		miR-379	TREM1, FGF-BP	
miR-196c	Decorin, TNF-*α*			miR-217	Notch 2, IL-1*α*	

∗ indicates a functional but nonpredominant miRNA as indicated by miRbase.

**Table 6:** miRNAs associated with cardiac fibrosis, their overall effect on fibrosis/TGF-*β* signaling, and the predicted target genes along the TGF-*β* signaling pathway.

MicroRNA	Assoc. with fibrosis (*r* ^2^, *p* value)	Fibrotic effect	Predicted gene targets in the TGF-beta signaling pathway^†^
hsa-miR-140	**0.6443**, **0.034**	Anti [1]	*PITX2*, *BMP2*
hsa-miR-144	**−0.83**, **0.002**	Anti [2, 3]	**ACVR2B**, *SMAD9*, *ROCK1*, *BMPR1B*, CDKN2B, ID4
hsa-miR-155	**0.639**, **0.036**	Pro/anti^∗^ [4, 5]	*ACVR1C*, *BMPR2*, *SMAD5*, *SMAD2*, PPP2CA, *GDF6*, *SP1*, *RPS6KB1*
hsa-miR-21	**0.633**, **0.039**	Pro [6–8]	**ACVR2A**, *BMPR2*, SMAD7, TGFB2
hsa-miR-26b	**0.687**, **0.02**	Pro/anti^∗^ [9, 10]	*ACVR1C*, *BMPR1B*, *BMPR2*, *CREBBP*, *EP300*, IFN*γ*, INHBA, INHBB, *SMAD1*, *SMAD2*
hsa-miR-34b	**0.742**, **0.008**	Pro [11]	**ACVR2A**, *ACVR1C*, FST, *SMAD5*, SMAD7, SMURF1, *RPS6KB1*

^∗^Physiological conditionally based effects. ^†^Emphasis indicates the predicted effect that miRNA inactivation of that gene would have on fibrosis. Italic emphasis refers to decreased fibrosis. Underlined emphasis refers to increased fibrosis. Bold emphasis refers to effect dependent on ligand milieu. ACVR1C: activin receptor type-1C; ACVR2A: activin receptor type-2A; ACVR2B: activin receptor type-2B; BMP2: bone morphological protein-2; BMPR2: bone morphological protein receptor type-2; CDKN2B: cyclin-dependent kinase inhibitor 2B; CREBBP: cAMP response element-binding protein; EP300: E1A-associated protein p300; FST: follistatin; GDF6: growth differentiation factor 6; ID4: inhibitor of DNA-binding protein 4; IFN*γ*: interferon-gamma; INHBA: inhibin beta A subunit; INHBB: inhibin beta B subunit; PITX2: paired-like homeodomain transcription factor 2; PPP2CA: protein phosphatase 2 catalytic subunit alpha; ROCK1: Rho-associated coiled-coil-containing protein; RPS6KB1: ribosomal protein S6 kinase B1; SMURF1: SMAD-specific E3 ubiquitin protein ligase 1; TGFB2: transforming growth factor beta 2.

## Data Availability

The data used to support the findings of this study are available from the corresponding author upon request.

## Supplementary Materials

Supplementary Materials Supplemental Table 1: differentially expressed miRNAs that were significant (*p* < 0.05) in ZO-C vs. ZL-C hearts. Supplemental Table 2: differentially expressed miRNAs that were significant (*p* < 0.05) in ZL-Rap vs. ZL-C hearts. Supplemental Table 3: differentially expressed miRNAs that were significant (*p* < 0.05) in ZO-Rap vs. ZO-C hearts. Supplemental Table 4: differentially expressed miRNAs that were significant (*p* < 0.05) in ZO-Rap vs. ZO-C hearts. Supplemental Table 5: KEGG pathway enrichment analysis of pathways corresponding to differentially expressed miRNA^∗^.

## References

[1] Muller L. M. A. J., Gorter K. J., Hak E. (2005). Increased risk of common infections in patients with type 1 and type 2 diabetes mellitus. *Clinical Infectious Diseases*.

[2] Chirillo F., Bacchion F., Pedrocco A. (2010). Infective endocarditis in patients with diabetes mellitus. *The Journal of Heart Valve Disease*.

[3] Luck C., DeMarco V. G., Mahmood A., Gavini M. P., Pulakat L. (2017). Differential regulation of cardiac function and intracardiac cytokines by rapamycin in healthy and diabetic rats. *Oxidative Medicine and Cellular Longevity*.

[4] Gude E., Gullestad L., Andreassen A. K. (2017). Everolimus immunosuppression for renal protection, reduction of allograft vasculopathy and prevention of allograft rejection in de-novo heart transplant recipients: could we have it all?. *Current Opinion in Organ Transplantation*.

[5] Pascual J., Royuela A., Fernández A. M. (2016). Role of mTOR inhibitors for the control of viral infection in solid organ transplant recipients. *Transplant Infectious Disease*.

[6] De Simone P., Fagiuoli S., Cescon M. (2017). Use of everolimus in liver transplantation: recommendations from a working group. *Transplantation*.

[7] Forbes S. A., Bindal N., Bamford S. (2010). COSMIC: mining complete cancer genomes in the Catalogue of Somatic Mutations in Cancer. *Nucleic Acids Research*.

[8] Martelli A. M., Buontempo F., McCubrey J. A. (2018). Drug discovery targeting the mTOR pathway. *Clinical Science*.

[9] Tannir N. M., Pal S. K., Atkins M. B. (2018). Second‐line treatment landscape for renal cell carcinoma: a comprehensive review. *The Oncologist*.

[10] Castrellon A. B. (2017). Novel strategies to improve the endocrine therapy of breast cancer. *Oncology Reviews*.

[11] Fazio N., Buzzoni R., Delle Fave G. (2018). Everolimus in advanced, progressive, well-differentiated, non-functional neuroendocrine tumors: RADIANT-4 lung subgroup analysis. *Cancer Science*.

[12] Guri Y., Nordmann T. M., Roszik J. (2018). mTOR at the transmitting and receiving ends in tumor immunity. *Frontiers in Immunology*.

[13] Martinet W., de Loof H., de Meyer G. R. Y. (2014). mTOR inhibition: a promising strategy for stabilization of atherosclerotic plaques. *Atherosclerosis*.

[14] Weichhart T. (2018). mTOR as regulator of lifespan, aging, and cellular senescence: a mini-review. *Gerontology*.

[15] Harrison D. E., Strong R., Sharp Z. D. (2009). Rapamycin fed late in life extends lifespan in genetically heterogeneous mice. *Nature*.

[16] Johnson S. C., Yanos M. E., Bitto A. (2015). Dose-dependent effects of mTOR inhibition on weight and mitochondrial disease in mice. *Frontiers in Genetics*.

[17] Morviducci L., Rota F., Rizza L. (2018). Everolimus is a new anti-cancer molecule: metabolic side effects as lipid disorders and hyperglycemia. *Diabetes Research and Clinical Practice*.

[18] Murakami N., Riella L. V., Funakoshi T. (2014). Risk of metabolic complications in kidney transplantation after conversion to mTOR inhibitor: a systematic review and meta-analysis. *American Journal of Transplantation*.

[19] Barlow A. D., Nicholson M. L., Herbert T. P. (2013). Evidence for rapamycin toxicity in pancreatic *β*-cells and a review of the underlying molecular mechanisms. *Diabetes*.

[20] Sataranatarajan K., Ikeno Y., Bokov A. (2016). Rapamycin increases mortality in *db*/*db* mice, a mouse model of type 2 diabetes. *The Journals of Gerontology Series A: Biological Sciences and Medical Sciences*.

[21] Trindade A. J., Medvetz D. A., Neuman N. A. (2013). MicroRNA-21 is induced by rapamycin in a model of tuberous sclerosis (TSC) and lymphangioleiomyomatosis (LAM). *PLoS One*.

[22] Zhou Y., Zhao R. H., Tseng K. F. (2016). Sirolimus induces apoptosis and reverses multidrug resistance in human osteosarcoma cells *in vitro* via increasing microRNA-34b expression. *Acta Pharmacologica Sinica*.

[23] Papadopoulos E. I., Yousef G. M., Scorilas A. (2015). Cytotoxic activity of sunitinib and everolimus in Caki-1 renal cancer cells is accompanied by modulations in the expression of apoptosis-related microRNA clusters and *BCL2* family genes. *Biomedicine & Pharmacotherapy*.

[24] Cifarelli V., Lashinger L. M., Devlin K. L. (2015). Metformin and rapamycin reduce pancreatic cancer growth in obese prediabetic mice by distinct microRNA-regulated mechanisms. *Diabetes*.

[25] Cheng K., Rai P., Plagov A. (2013). Rapamycin-induced modulation of miRNA expression is associated with amelioration of HIV-associated nephropathy (HIVAN). *Experimental Cell Research*.

[26] Roccaro A. M., Sacco A., Jia X. (2012). Mechanisms of activity of the TORC1 inhibitor everolimus in Waldenstrom macroglobulinemia. *Clinical Cancer Research*.

[27] Bartel D. P. (2009). MicroRNAs: target recognition and regulatory functions. *Cell*.

[28] Hammond S. M. (2015). An overview of microRNAs. *Advanced Drug Delivery Reviews*.

[29] Chang T. H., Huang H. Y., Hsu J. B., Weng S. L., Horng J. T., Huang H. D. (2013). An enhanced computational platform for investigating the roles of regulatory RNA and for identifying functional RNA motifs. *BMC Bioinformatics*.

[30] Vlachos I. S., Zagganas K., Paraskevopoulou M. D. (2015). DIANA-miRPath v3.0: deciphering microRNA function with experimental support. *Nucleic Acids Research*.

[31] García-Díaz D. F., Pizarro C., Camacho-Guillén P., Codner E., Soto N., Pérez-Bravo F. (2018). Expression of miR-155, miR-146a, and miR-326 in T1D patients from Chile: relationship with autoimmunity and inflammatory markers. *Archives of Endocrinology and Metabolism*.

[32] Assmann T. S., Recamonde-Mendoza M., Puñales M., Tschiedel B., Canani L. H., Crispim D. (2018). MicroRNA expression profile in plasma from type 1 diabetic patients: case-control study and bioinformatic analysis. *Diabetes Research and Clinical Practice*.

[33] Seok H. Y., Chen J., Kataoka M. (2014). Loss of microRNA-155 protects the heart from pathological cardiac hypertrophy. *Circulation Research*.

[34] Qiu X. K., Ma J. (2018). Alteration in microRNA-155 level correspond to severity of coronary heart disease. *Scandinavian Journal of Clinical and Laboratory Investigation*.

[35] Alivernini S., Gremese E., McSharry C. (2018). MicroRNA-155—at the critical interface of innate and adaptive immunity in arthritis. *Frontiers in Immunology*.

[36] Arnold N., Koppula P. R., Gul R., Luck C., Pulakat L. (2014). Regulation of cardiac expression of the diabetic marker microRNA miR-29. *PLoS One*.

[37] Roggli E., Gattesco S., Caille D. (2012). Changes in microRNA expression contribute to pancreatic *β*-cell dysfunction in prediabetic NOD mice. *Diabetes*.

[38] Ślusarz A., Pulakat L. (2015). The two faces of miR-29. *Journal of Cardiovascular Medicine (Hagerstown, Md.)*.

[39] Chavali V., Tyagi S. C., Mishra P. K. (2014). Differential expression of dicer, miRNAs, and inflammatory markers in diabetic Ins2^+/−^ Akita hearts. *Cell Biochemistry and Biophysics*.

[40] Kuwabara Y., Horie T., Baba O. (2015). MicroRNA-451 exacerbates lipotoxicity in cardiac myocytes and high-fat diet-induced cardiac hypertrophy in mice through suppression of the LKB1/AMPK pathway. *Circulation Research*.

[41] Liang Y. Z., Dong J., Zhang J., Wang S., He Y., Yan Y. X. (2018). Identification of neuroendocrine stress response-related circulating microRNAs as biomarkers for type 2 diabetes mellitus and insulin resistance. *Frontiers in Endocrinology*.

[42] Wang W., Yang C., Wang X. (2018). MicroRNA-129 and -335 promote diabetic wound healing by inhibiting Sp1-mediated MMP-9 expression. *Diabetes*.

[43] Shi Q., Gibson G. E. (2011). Up-regulation of the mitochondrial malate dehydrogenase by oxidative stress is mediated by miR-743a. *Journal of Neurochemistry*.

[44] Karagiannis G. S., Weile J., Bader G. D., Minta J. (2013). Integrative pathway dissection of molecular mechanisms of moxLDL-induced vascular smooth muscle phenotype transformation. *BMC Cardiovascular Disorders*.

[45] Pardo P. S., Hajira A., Boriek A. M., Mohamed J. S. (2017). MicroRNA-434-3p regulates age-related apoptosis through eIF5A1 in the skeletal muscle. *Aging*.

[46] Wang X. T., Wu X. D., Lu Y. X. (2018). Potential involvement of MiR-30e-3p in myocardial injury induced by coronary microembolization via autophagy activation. *Cellular Physiology and Biochemistry*.

[47] Borosch S., Dahmen E., Beckers C. (2017). Characterization of extracellular vesicles derived from cardiac cells in an *in vitro* model of preconditioning. *Journal of Extracellular Vesicles*.

[48] Cardin S., Guasch E., Luo X. (2012). Role for microRNA-21 in atrial profibrillatory fibrotic remodeling associated with experimental postinfarction heart failure. *Circulation: Arrhythmia and Electrophysiology*.

[49] Dong S., Ma W., Hao B. (2014). MicroRNA-21 promotes cardiac fibrosis and development of heart failure with preserved left ventricular ejection fraction by up-regulating Bcl-2. *International Journal of Clinical and Experimental Pathology*.

[50] Yuan J., Chen H., Ge D. (2017). Mir-21 promotes cardiac fibrosis after myocardial infarction via targeting Smad 7. *Cellular Physiology and Biochemistry*.

[51] Bernardo B. C., Gao X. M., Winbanks C. E. (2012). Therapeutic inhibition of the miR-34 family attenuates pathological cardiac remodeling and improves heart function. *Proceedings of the National Academy of Sciences of the United States of America*.

[52] Ooi J. Y. Y., Bernardo B. C., Singla S., Patterson N. L., Lin R. C. Y., McMullen J. R. (2016). Identification of miR-34 regulatory networks in settings of disease and antimiR-therapy: implications for treating cardiac pathology and other diseases. *RNA Biology*.

[53] Wang C., Song X., Li Y. (2013). Low-dose paclitaxel ameliorates pulmonary fibrosis by suppressing TGF-*β*1/Smad3 pathway via miR-140 upregulation. *PLoS One*.

[54] Wenqi Z., Hong C., Hexun Z., Lei Z. (2017). MiR-30e attenuates isoproterenol-induced cardiac fibrosis through suppressing Snai1/TGF-*β* signaling. *Journal of Cardiovascular Pharmacology*.

[55] Wei Y., Yan X., Yan L. (2017). Inhibition of microRNA-155 ameliorates cardiac fibrosis in the process of angiotensin II-induced cardiac remodeling. *Molecular Medicine Reports*.

[56] Zhang G., Shi H., Wang L. (2015). MicroRNA and transcription factor mediated regulatory network analysis reveals critical regulators and regulatory modules in myocardial infarction. *PLoS One*.

[57] Ruan C., Lu J., Wang H., Ge Z., Zhang C., Xu M. (2017). miR-26b-5p regulates hypoxia-induced phenotypic switching of vascular smooth muscle cells via the TGF-*β*/Smad4 signaling pathway. *Molecular Medicine Reports*.

[58] Tang C. M., Zhang M., Huang L. (2017). CircRNA_000203 enhances the expression of fibrosis-associated genes by derepressing targets of miR-26b-5p, Col1a2 and CTGF, in cardiac fibroblasts. *Scientific Reports*.

[59] Lakhter A. J., Pratt R. E., Moore R. E. (2018). Beta cell extracellular vesicle miR-21-5p cargo is increased in response to inflammatory cytokines and serves as a biomarker of type 1 diabetes. *Diabetologia*.

[60] Chen C., Lu C., Qian Y. (2017). Urinary miR-21 as a potential biomarker of hypertensive kidney injury and fibrosis. *Scientific Reports*.

[61] Hijmans J. G., Diehl K. J., Bammert T. D. (2018). Association between hypertension and circulating vascular-related microRNAs. *Journal of Human Hypertension*.

[62] Gu H., Liu Z., Li Y. (2018). Serum-derived extracellular vesicles protect against acute myocardial infarction by regulating miR-21/PDCD4 signaling pathway. *Frontiers in Physiology*.

[63] Luther K. M., Haar L., McGuinness M. (2018). Exosomal miR-21a-5p mediates cardioprotection by mesenchymal stem cells. *Journal of Molecular and Cellular Cardiology*.

[64] Gangwar R. S., Rajagopalan S., Natarajan R., Deiuliis J. A. (2018). Noncoding RNAs in cardiovascular disease: pathological relevance and emerging role as biomarkers and therapeutics. *American Journal of Hypertension*.

[65] Lam H. C., Liu H. J., Baglini C. V. (2017). Rapamycin-induced miR-21 promotes mitochondrial homeostasis and adaptation in mTORC1 activated cells. *Oncotarget*.

[66] Wan G., Xie W., Liu Z. (2014). Hypoxia-induced *MIR155* is a potent autophagy inducer by targeting multiple players in the MTOR pathway. *Autophagy*.

[67] Lee J. S., Yang D. K., Park J. H. (2017). MicroRNA-101b attenuates cardiomyocyte hypertrophy by inhibiting protein kinase C epsilon signaling. *FEBS Letters*.

[68] Totary-Jain H., Sanoudou D., Ben-Dov I. Z. (2013). Reprogramming of the microRNA transcriptome mediates resistance to rapamycin. *Journal of Biological Chemistry*.

[69] Leask A., Abraham D. J. (2004). TGF-beta signaling and the fibrotic response. *The FASEB Journal*.

[70] Yue Y., Meng K., Pu Y., Zhang X. (2017). Transforming growth factor beta (TGF-*β*) mediates cardiac fibrosis and induces diabetic cardiomyopathy. *Diabetes Research and Clinical Practice*.

[71] Ludwig N., Leidinger P., Becker K. (2016). Distribution of miRNA expression across human tissues. *Nucleic Acids Research*.

[72] Quach H., Barreiro L. B., Laval G. (2009). Signatures of purifying and local positive selection in human miRNAs. *American Journal of Human Genetics*.

[73] Reddy M. A., Jin W., Villeneuve L. (2012). Pro-inflammatory role of microrna-200 in vascular smooth muscle cells from diabetic mice. *Arteriosclerosis, Thrombosis, and Vascular Biology*.

[74] Pulakat L., Aroor A. R., Gul R., Sowers J. R. (2012). Cardiac insulin resistance and microRNA modulators. *Experimental Diabetes Research*.

[75] Yu Y., Bai F., Qin N. (2018). Non-proximal renal tubule-derived urinary exosomal miR-200b as a biomarker of renal fibrosis. *Nephron*.

[76] D’Agostino M., Martino F., Sileno S. (2017). Circulating *miR-200c* is up-regulated in paediatric patients with familial hypercholesterolaemia and correlates with *miR-33a/b* levels: implication of a ZEB1-dependent mechanism. *Clinical Science (London, England)*.

[77] Pan Z., Sun X., Shan H. (2012). MicroRNA-101 inhibited postinfarct cardiac fibrosis and improved left ventricular compliance via the FBJ osteosarcoma oncogene/transforming growth factor-*β*1 pathway. *Circulation*.

[78] Marketou M. E., Kontaraki J. E., Maragkoudakis S. (2018). MicroRNAs in peripheral mononuclear cells as potential biomarkers in hypertensive patients with heart failure with preserved ejection fraction. *American Journal of Hypertension*.

[79] Wang L., Zhang N., Pan H. P., Wang Z., Cao Z. Y. (2015). MiR-499-5p contributes to hepatic insulin resistance by suppressing PTEN. *Cellular Physiology and Biochemistry*.

[80] Corsten M. F., Dennert R., Jochems S. (2010). Circulating microRNA-208b and microRNA-499 reflect myocardial damage in cardiovascular disease. *Circulation: Cardiovascular Genetics*.

[81] da Silva A. M. G., de Araújo J. N. G., de Oliveira K. M. (2018). Circulating miRNAs in acute new-onset atrial fibrillation and their target mRNA network. *Journal of Cardiovascular Electrophysiology*.

[82] Khanaghaei M., Tourkianvalashani F., Hekmatimoghaddam S. (2016). Circulating miR-126 and miR-499 reflect progression of cardiovascular disease; correlations with uric acid and ejection fraction. *Heart International*.

[83] Escate R., Mata P., Cepeda J. M., Padró T., Badimon L. (2018). miR-505-3p controls chemokine receptor up-regulation in macrophages: role in familial hypercholesterolemia. *The FASEB Journal*.

[84] Heilmeier U., Hackl M., Skalicky S. (2016). Serum miRNA signatures are indicative of skeletal fractures in postmenopausal women with and without type 2 diabetes and influence osteogenic and adipogenic differentiation of adipose tissue-derived mesenchymal stem cells in vitro. *Journal of Bone and Mineral Research*.

[85] Fang Y., Xie T., Xue N. (2017). miR-382 contributes to renal tubulointerstitial fibrosis by downregulating HSPD1. *Oxidative Medicine and Cellular Longevity*.

[86] Hulsmans M., de Keyzer D., Holvoet P. (2011). MicroRNAs regulating oxidative stress and inflammation in relation to obesity and atherosclerosis. *The FASEB Journal*.

[87] Pereira B. L., Arruda F. C., Reis P. P. (2015). Tomato (Lycopersicon esculentum) supplementation induces changes in cardiac miRNA expression, reduces oxidative stress and left ventricular mass, and improves diastolic function. *Nutrients*.

[88] Shao Y., Ren H., Lv C., Ma X., Wu C., Wang Q. (2017). Changes of serum Mir-217 and the correlation with the severity in type 2 diabetes patients with different stages of diabetic kidney disease. *Endocrine*.

[89] Shao Y., Lv C., Wu C., Zhou Y., Wang Q. (2016). Mir-217 promotes inflammation and fibrosis in high glucose cultured rat glomerular mesangial cells via Sirt 1/HIF-1*α* signaling pathway. *Diabetes/Metabolism Research and Reviews*.

[90] Seeger T., Boon R. A. (2016). MicroRNAs in cardiovascular ageing. *The Journal of Physiology*.

[91] Long Y., Zhan Q., Yuan M. (2017). The expression of microRNA-223 and FAM5C in cerebral infarction patients with diabetes mellitus. *Cardiovascular Toxicology*.

[92] Liu X., Xu Y., Deng Y., Li H. (2018). MicroRNA-223 regulates cardiac fibrosis after myocardial infarction by targeting RASA1. *Cellular Physiology and Biochemistry*.

[93] Liu X., Zhang Y., Du W. (2016). MiR-223-3p as a novel microRNA regulator of expression of voltage-gated K^+^ channel Kv4.2 in acute myocardial infarction. *Cellular Physiology and Biochemistry*.

[94] Ai P., Shen B., Pan H., Chen K., Zheng J., Liu F. (2018). MiR-411 suppressed vein wall fibrosis by downregulating MMP-2 via targeting HIF-1*α*. *Journal of Thrombosis and Thrombolysis*.

[95] Stather P. W., Sylvius N., Sidloff D. A. (2015). Identification of microRNAs associated with abdominal aortic aneurysms and peripheral arterial disease. *British Journal of Surgery*.

[96] Panguluri S. K., Tur J., Chapalamadugu K. C., Katnik C., Cuevas J., Tipparaju S. M. (2013). MicroRNA-301a mediated regulation of Kv4.2 in diabetes: identification of key modulators. *PLoS One*.

[97] Welten S. M. J., Bastiaansen A. J. N. M., de Jong R. C. M. (2014). Inhibition of 14q32 microRNAs miR-329, miR-487b, miR-494, and miR-495 increases neovascularization and blood flow recovery after ischemia. *Circulation Research*.

[98] Ge Y., Pan S., Guan D. (2013). MicroRNA-350 induces pathological heart hypertrophy by repressing both p38 and JNK pathways. *Biochimica et Biophysica Acta*.

[99] Fejes Z., Póliska S., Czimmerer Z. (2017). Hyperglycaemia suppresses microRNA expression in platelets to increase P2RY12 and SELP levels in type 2 diabetes mellitus. *Thrombosis and Haemostasis*.

[100] Duru N., Zhang Y., Gernapudi R. (2016). Loss of miR-140 is a key risk factor for radiation-induced lung fibrosis through reprogramming fibroblasts and macrophages. *Scientific Reports*.

[101] Rothman A. M. K., Arnold N. D., Pickworth J. A. (2016). MicroRNA-140-5p and SMURF1 regulate pulmonary arterial hypertension. *Journal of Clinical Investigation*.

[102] Gu H., Yu J., Dong D., Zhou Q., Wang J. Y., Yang P. (2015). The miR-322-TRAF3 circuit mediates the pro-apoptotic effect of high glucose on neural stem cells. *Toxicological Sciences*.

[103] Marchand A., Atassi F., Mougenot N. (2016). miR-322 regulates insulin signaling pathway and protects against metabolic syndrome-induced cardiac dysfunction in mice. *Biochimica et Biophysica Acta*.

[104] Xie Y., Jia Y., Cuihua X., Hu F., Xue M., Xue Y. (2017). Urinary exosomal microRNA profiling in incipient type 2 diabetic kidney disease. *Journal of Diabetes Research*.

[105] Li M., Liu Q., Lei J., Wang X., Chen X., Ding Y. (2017). MiR-362-3p inhibits the proliferation and migration of vascular smooth muscle cells in atherosclerosis by targeting ADAMTS1. *Biochemical and Biophysical Research Communications*.

[106] Lee J. S., Song D. W., Park J. H., Kim J. O., Cho C., Kim D. H. (2017). miR-374 promotes myocardial hypertrophy by negatively regulating vascular endothelial growth factor receptor-1 signaling. *BMB Reports*.

[107] Licholai S., Blaż M., Kapelak B., Sanak M. (2016). Unbiased profile of microRNA expression in ascending aortic aneurysm tissue appoints molecular pathways contributing to the pathology. *The Annals of Thoracic Surgery*.

[108] Xu G., Chen J., Jing G., Shalev A. (2013). Thioredoxin-interacting protein regulates insulin transcription through microRNA-204. *Nature Medicine*.

[109] Kassan M., Vikram A., Kim Y. R. (2017). Sirtuin 1 protects endothelial Caveolin-1 expression and preserves endothelial function via suppressing miR-204 and endoplasmic reticulum stress. *Scientific Reports*.

[110] Cao J. L., Zhang L., Li J. (2016). Up-regulation of miR-98 and unraveling regulatory mechanisms in gestational diabetes mellitus. *Scientific Reports*.

[111] Cheng R., Dang R., Zhou Y., Ding M., Hua H. (2017I). MicroRNA-98 inhibits TGF-*β*1-induced differentiation and collagen production of cardiac fibroblasts by targeting TGFBR1. *Human Cell*.

[112] Zhang B. Y., Zhao Z., Jin Z. (2016). Expression of miR-98 in myocarditis and its influence on transcription of the *FAS*/*FASL* gene pair. *Genetics and Molecular Research*.

[113] Song J., Su W., Chen X. (2017). Micro RNA-98 suppresses interleukin-10 in peripheral B cells in patient post-cardio transplantation. *Oncotarget*.

[114] Greco S., Fasanaro P., Castelvecchio S. (2012). MicroRNA dysregulation in diabetic ischemic heart failure patients. *Diabetes*.

[115] Joglekar M. V., Parekh V. S., Hardikar A. A. (2007). New pancreas from old: microregulators of pancreas regeneration. *Trends in Endocrinology and Metabolism*.

[116] Liu F., Li N., Long B. (2014). Cardiac hypertrophy is negatively regulated by miR-541. *Cell Death & Disease*.

[117] Li K., Wang Y., Zhang A., Liu B., Jia L. (2017). miR-379 inhibits cell proliferation, invasion, and migration of vascular smooth muscle cells by targeting insulin-like factor-1. *Yonsei Medical Journal*.

[118] Zhang Y., Huang X. R., Wei L. H., Chung A. C. K., Yu C. M., Lan H. Y. (2014). miR-29b as a therapeutic agent for angiotensin II-induced cardiac fibrosis by targeting TGF-*β*/Smad3 signaling. *Molecular Therapy*.

[119] Witkowski M., Tabaraie T., Steffens D. (2018). MicroRNA-19a contributes to the epigenetic regulation of tissue factor in diabetes. *Cardiovascular Diabetology*.

[120] Zou M., Wang F., Gao R. (2016). Autophagy inhibition of hsa-miR-19a-3p/19b-3p by targeting TGF-*β* R II during TGF-*β*1-induced fibrogenesis in human cardiac fibroblasts. *Scientific Reports*.

[121] Miao Y., Chen H., Li M. (2015). MiR-19a overexpression contributes to heart failure through targeting ADRB1. *International Journal of Clinical and Experimental Medicine*.

[122] Chen H., Li X., Liu S., Gu L., Zhou X. (2017). MircroRNA-19a promotes vascular inflammation and foam cell formation by targeting *HBP*-1 in atherogenesis. *Scientific Reports*.

[123] Muthusamy S., DeMartino A. M., Watson L. J. (2014). MicroRNA-539 is up-regulated in failing heart, and suppresses *O*-GlcNAcase expression. *Journal of Biological Chemistry*.

[124] Wang K., Long B., Zhou L. Y. (2014). CARL lncRNA inhibits anoxia-induced mitochondrial fission and apoptosis in cardiomyocytes by impairing miR-539-dependent PHB2 downregulation. *Nature Communications*.

[125] Argyropoulos C., Wang K., Bernardo J. (2015). Urinary microRNA profiling predicts the development of microalbuminuria in patients with type 1 diabetes. *Journal of Clinical Medicine*.

[126] Meng X., Ji Y., Wan Z. (2017). Inhibition of miR-363 protects cardiomyocytes against hypoxia-induced apoptosis through regulation of Notch signaling. *Biomedicine & Pharmacotherapy*.

[127] Goren Y., Kushnir M., Zafrir B., Tabak S., Lewis B. S., Amir O. (2012). Serum levels of microRNAs in patients with heart failure. *European Journal of Heart Failure*.

[128] Fu J., Chen Y., Li F. (2018). Attenuation of microRNA-495 derepressed PTEN to effectively protect rat cardiomyocytes from hypertrophy. *Cardiology*.

[129] Clark A. L., Maruyama S., Sano S. (2016). miR-410 and miR-495 are dynamically regulated in diverse cardiomyopathies and their inhibition attenuates pathological hypertrophy. *PLoS One*.

[130] Latreille M., Hausser J., Stützer I. (2014). MicroRNA-7a regulates pancreatic *β* cell function. *Journal of Clinical Investigation*.

[131] Li R., Xiao J., Qing X. (2015). Sp1 mediates a therapeutic role of MiR-7a/b in angiotensin II-induced cardiac fibrosis via mechanism involving the TGF-*β* and MAPKs pathways in cardiac fibroblasts. *PLoS One*.

[132] Li B., Li R., Zhang C. (2014). MicroRNA-7a/b protects against cardiac myocyte injury in ischemia/reperfusion by targeting poly(ADP-ribose) polymerase. *PLoS One*.

[133] Yu T., Lu X. J., Li J. Y. (2016). Overexpression of miR-429 impairs intestinal barrier function in diabetic mice by down-regulating occludin expression. *Cell and Tissue Research*.

[134] Xu H., Jin L., Chen Y., Li J. (2016). Downregulation of microRNA-429 protects cardiomyocytes against hypoxia-induced apoptosis by increasing Notch1 expression. *International Journal of Molecular Medicine*.

[135] Wang E. W., Jia X. S., Ruan C. W., Ge Z. R. (2017). miR-487b mitigates chronic heart failure through inhibition of the IL-33/ST2 signaling pathway. *Oncotarget*.

[136] Wang X., Zhu H., Zhang X. (2012). Loss of the miR-144/451 cluster impairs ischaemic preconditioning-mediated cardioprotection by targeting Rac-1. *Cardiovascular Research*.

[137] Fakhry M., Skafi N., Fayyad-Kazan M. (2018). Characterization and assessment of potential microRNAs involved in phosphate-induced aortic calcification. *Journal of Cellular Physiology*.

