# Supplementary material for: Comparison of Cardiac miRNA Transcriptomes Induced by Diabetes and Rapamycin Treatment and Identification of a Rapamycin-Associated Cardiac MicroRNA Signature
Source: Oxid Med Cell Longev. 2018 Dec 17;2018:8364608. doi: 10.1155/2018/8364608 (PMC6311877; doi:10.1155/2018/8364608)
Supplement: Supplementary Materials — Supplemental Table 1: differentially expressed miRNAs that were significant (p < 0.05) in ZO-C vs. ZL-C hearts. Supplemental Table 2: differentially expressed miRNAs that were significant (p < 0.05) in ZL-Rap vs. ZL-C hearts. Supplemental Table 3: differentially expressed miRNAs that were significant (p < 0.05) in ZO-Rap vs. ZO-C hearts. Supplemental Table 4: differentially expressed miRNAs that were significant (p < 0.05) in ZO-Rap vs. ZO-C hearts. Supplemental Table 5: KEGG pathway enrichment analysis of pathways corresponding to differentially expressed miRNA∗. [file 8364608.f1.docx]

**Supplementary Data**

| **Supplemental Table 1. Differentially expressed miRNA that were significant (*p*<0.05) in ZO-C vs. ZL-C rat hearts.** | | | | | | | | | | | | | | | | | | |
| --- | --- | --- | --- | --- | --- | --- | --- | --- | --- | --- | --- | --- | --- | --- | --- | --- | --- | --- |
|  | ZL-C | ZL-C | ZL-C | ZL-C | ZL-C | AVG | STE | ZO-C | ZO-C | ZO-C | ZO-C | ZO-C | AVG | STE | p-value | ZO/ZL | Log Ratio | Fold Diff |
| rno-miR-434-3p | 1.954 | 1.021 | 1.197 | 2.565 | 3.260 | 2.000 | 0.419 | 5.097 | 4.930 | 6.290 | 5.815 | 5.341 | 5.495 | 0.249 | 0.000 | 2.748 | 2.748 | 7.551 |
| rno-miR-155-5p | 2.088 | 1.095 | 1.622 | 4.637 | 5.950 | 3.078 | 0.732 | 6.770 | 7.341 | 7.313 | 7.303 | 7.939 | 7.333 | 0.185 | 0.002 | 2.382 | 2.382 | 5.675 |
| rno-miR-31a-3p | 1.318 | 1.312 | 1.034 | 1.444 | 1.629 | 1.347 | 0.751 | 2.680 | 3.557 | 4.580 | 3.985 | 1.219 | 3.204 | 0.585 | 0.014 | 2.378 | 2.378 | 5.656 |
| rno-miR-200b-3p | 2.117 | 0.882 | 1.049 | 1.740 | 3.295 | 1.817 | 0.316 | 3.404 | 4.154 | 4.934 | 5.566 | 3.464 | 4.304 | 0.420 | 0.003 | 2.369 | 2.369 | 5.614 |
| rno-miR-21-5p | 1.051 | 1.012 | 0.984 | 5.808 | 7.072 | 3.185 | 0.995 | 7.116 | 7.545 | 7.439 | 6.907 | 6.162 | 7.034 | 0.246 | 0.023 | 2.208 | 2.208 | 4.876 |
| rno-miR-30e-3p | 1.407 | 1.001 | 1.099 | 7.542 | 8.062 | 3.822 | 1.415 | 8.480 | 8.066 | 8.532 | 8.493 | 7.843 | 8.283 | 0.139 | 0.026 | 2.167 | 2.167 | 4.695 |
| rno-miR-7a-1-3p | 1.144 | 1.156 | 0.976 | 2.555 | 3.566 | 1.879 | 1.226 | 4.607 | 4.095 | 5.210 | 4.580 | 1.779 | 4.055 | 0.596 | 0.024 | 2.157 | 2.157 | 4.654 |
| rno-miR-26b-5p | 1.382 | 1.204 | 1.169 | 5.953 | 6.958 | 3.333 | 0.983 | 7.141 | 7.154 | 7.144 | 7.454 | 6.834 | 7.145 | 0.098 | 0.018 | 2.144 | 2.144 | 4.596 |
| rno-miR-872-5p | 1.191 | 0.994 | 0.725 | 0.900 | 1.262 | 1.014 | 1.018 | 2.388 | 2.013 | 3.319 | 1.605 | 1.396 | 2.144 | 0.340 | 0.013 | 2.114 | 2.114 | 4.467 |
| rno-miR-200c-3p | 4.044 | 2.092 | 2.495 | 4.670 | 5.514 | 3.763 | 0.781 | 6.907 | 7.621 | 7.469 | 6.961 | 8.894 | 7.570 | 0.359 | 0.001 | 2.012 | 2.012 | 4.048 |
| rno-miR-411-5p | 1.102 | 0.898 | 1.293 | 0.895 | 1.003 | 1.038 | 0.775 | 2.602 | 1.908 | 2.747 | 1.925 | 0.974 | 2.031 | 0.315 | 0.015 | 1.956 | 1.956 | 3.826 |
| rno-miR-382-5p | 2.998 | 0.887 | 2.511 | 1.756 | 3.723 | 2.375 | 0.457 | 3.797 | 3.634 | 4.487 | 5.169 | 6.048 | 4.627 | 0.448 | 0.010 | 1.948 | 1.948 | 3.795 |
| rno-miR-301a-3p | 1.063 | 1.179 | 1.508 | 3.498 | 5.109 | 2.472 | 0.622 | 5.623 | 5.064 | 4.968 | 4.721 | 3.597 | 4.795 | 0.334 | 0.027 | 1.940 | 1.940 | 3.763 |
| rno-miR-138-1-3p | 1.752 | 1.045 | 1.380 | 3.928 | 2.043 | 2.030 | 0.635 | 2.794 | 4.437 | 4.093 | 5.188 | 3.171 | 3.937 | 0.432 | 0.021 | 1.940 | 1.940 | 3.762 |
| rno-miR-329-3p | 2.001 | 1.535 | 1.567 | 1.676 | 2.732 | 1.902 | 0.368 | 2.275 | 3.236 | 4.241 | 4.095 | 4.261 | 3.622 | 0.386 | 0.005 | 1.904 | 1.904 | 3.625 |
| rno-miR-350 | 2.754 | 1.279 | 1.640 | 6.224 | 6.787 | 3.737 | 0.898 | 7.186 | 7.194 | 6.957 | 7.430 | 6.422 | 7.038 | 0.171 | 0.023 | 1.883 | 1.883 | 3.547 |
| rno-miR-505-3p | 2.067 | 1.021 | 1.113 | 1.659 | 3.374 | 1.847 | 0.936 | 3.658 | 3.638 | 3.798 | 2.813 | 3.391 | 3.460 | 0.174 | 0.008 | 1.873 | 1.873 | 3.509 |
| rno-miR-191a-3p | 1.485 | 1.093 | 0.511 | 1.353 | 1.434 | 1.175 | 0.347 | 2.220 | 2.786 | 2.311 | 1.317 | 2.245 | 2.176 | 0.238 | 0.010 | 1.851 | 1.851 | 3.426 |
| rno-miR-1843-3p | 0.914 | 1.659 | 1.328 | 2.988 | 2.855 | 1.949 | 0.352 | 3.369 | 4.203 | 3.605 | 3.231 | 3.525 | 3.587 | 0.167 | 0.006 | 1.841 | 1.841 | 3.388 |
| rno-miR-140-5p | 1.676 | 2.120 | 1.412 | 6.337 | 7.245 | 3.758 | 0.977 | 7.642 | 7.047 | 7.663 | 7.421 | 4.806 | 6.916 | 0.539 | 0.049 | 1.840 | 1.840 | 3.387 |
| rno-miR-322-5p | 3.125 | 2.532 | 0.978 | 7.233 | 8.065 | 4.386 | 1.253 | 8.436 | 7.949 | 8.216 | 8.117 | 7.526 | 8.049 | 0.153 | 0.030 | 1.835 | 1.835 | 3.367 |
| rno-miR-362-3p | 1.029 | 1.003 | 1.057 | 1.014 | 1.871 | 1.195 | 1.196 | 1.728 | 2.899 | 2.529 | 1.894 | 1.752 | 2.160 | 0.235 | 0.010 | 1.808 | 1.808 | 3.269 |
| rno-miR-374-5p | 1.476 | 1.140 | 1.747 | 1.813 | 2.874 | 1.810 | 0.267 | 2.843 | 3.722 | 3.835 | 3.181 | 2.135 | 3.143 | 0.310 | 0.014 | 1.736 | 1.736 | 3.015 |
| rno-miR-223-3p | 1.700 | 1.900 | 1.436 | 2.564 | 2.988 | 2.117 | 0.282 | 3.225 | 4.879 | 3.571 | 3.006 | 3.471 | 3.631 | 0.327 | 0.008 | 1.715 | 1.715 | 2.940 |
| rno-miR-196c-5p | 1.140 | 0.783 | 1.238 | 1.551 | 1.079 | 1.158 | 0.307 | 2.389 | 1.788 | 2.294 | 2.274 | 1.097 | 1.968 | 0.242 | 0.018 | 1.700 | 1.700 | 2.889 |
| rno-miR-204-5p | 1.143 | 0.887 | 1.294 | 1.458 | 2.048 | 1.366 | 0.161 | 2.426 | 2.978 | 2.689 | 2.069 | 1.371 | 2.307 | 0.278 | 0.024 | 1.689 | 1.689 | 2.852 |
| rno-miR-98-5p | 4.829 | 2.088 | 1.445 | 3.645 | 4.981 | 3.398 | 0.687 | 5.172 | 5.464 | 5.966 | 5.794 | 6.133 | 5.706 | 0.173 | 0.014 | 1.679 | 1.679 | 2.820 |
| rno-miR-409b | 0.707 | 1.140 | 0.769 | 1.247 | 1.337 | 1.040 | 0.736 | 1.977 | 2.587 | 1.527 | 1.606 | 0.997 | 1.739 | 0.264 | 0.044 | 1.672 | 1.672 | 2.795 |
| rno-miR-34b-3p | 1.774 | 1.738 | 2.213 | 4.458 | 4.551 | 2.947 | 0.626 | 5.036 | 4.480 | 5.216 | 4.748 | 4.533 | 4.802 | 0.142 | 0.022 | 1.630 | 1.630 | 2.656 |
| rno-miR-361-3p | 2.424 | 2.181 | 1.002 | 2.492 | 4.624 | 2.544 | 0.587 | 3.673 | 4.935 | 4.593 | 4.408 | 3.083 | 4.138 | 0.335 | 0.046 | 1.627 | 1.627 | 2.646 |
| rno-miR-742-5p | 0.856 | 0.831 | 0.760 | 0.849 | 1.118 | 0.883 | 0.554 | 1.342 | 1.208 | 1.237 | 1.506 | 1.798 | 1.418 | 0.108 | 0.003 | 1.607 | 1.607 | 2.581 |
| rno-miR-217-5p | 1.640 | 1.522 | 1.188 | 1.749 | 1.393 | 1.498 | 0.164 | 2.334 | 2.579 | 3.185 | 2.393 | 1.345 | 2.367 | 0.297 | 0.024 | 1.580 | 1.580 | 2.497 |
| rno-miR-511-3p | 2.699 | 1.434 | 1.668 | 3.328 | 3.877 | 2.601 | 0.412 | 4.199 | 4.545 | 4.676 | 4.577 | 2.488 | 4.097 | 0.410 | 0.043 | 1.575 | 1.575 | 2.481 |
| rno-miR-541-5p | 4.870 | 2.577 | 4.922 | 1.468 | 2.345 | 3.236 | 0.583 | 4.147 | 4.680 | 6.009 | 5.192 | 5.307 | 5.067 | 0.313 | 0.044 | 1.566 | 1.566 | 2.452 |
| rno-miR-702-5p | 2.797 | 2.497 | 1.706 | 4.604 | 4.556 | 3.232 | 0.607 | 5.212 | 4.553 | 4.851 | 5.064 | 5.091 | 4.954 | 0.116 | 0.019 | 1.533 | 1.533 | 2.350 |
| rno-miR-6326 | 1.164 | 2.621 | 0.792 | 1.488 | 2.193 | 1.652 | 0.581 | 2.453 | 2.886 | 2.297 | 2.822 | 2.164 | 2.525 | 0.143 | 0.043 | 1.529 | 1.529 | 2.336 |
| rno-miR-664-1-5p | 4.177 | 3.871 | 2.893 | 2.093 | 4.589 | 3.525 | 0.580 | 4.751 | 5.449 | 5.216 | 4.976 | 6.379 | 5.354 | 0.281 | 0.009 | 1.519 | 1.519 | 2.308 |
| rno-miR-3102 | 1.850 | 1.115 | 1.441 | 1.484 | 1.289 | 1.436 | 0.584 | 2.224 | 2.764 | 1.621 | 2.608 | 1.684 | 2.180 | 0.233 | 0.022 | 1.519 | 1.519 | 2.306 |
| rno-miR-379-5p | 4.438 | 2.486 | 3.780 | 4.276 | 3.483 | 3.693 | 0.586 | 5.438 | 4.808 | 6.053 | 5.837 | 5.862 | 5.600 | 0.222 | 0.002 | 1.516 | 1.516 | 2.300 |
| rno-miR-10b-5p | 0.813 | 1.498 | 1.224 | 2.137 | 1.336 | 1.402 | 0.605 | 2.121 | 2.012 | 2.270 | 2.113 | 1.821 | 2.067 | 0.074 | 0.019 | 1.475 | 1.475 | 2.176 |
| rno-miR-154-5p | 1.363 | 0.788 | 1.264 | 1.003 | 1.184 | 1.121 | 0.172 | 1.408 | 2.071 | 1.872 | 1.586 | 1.240 | 1.635 | 0.151 | 0.022 | 1.460 | 1.460 | 2.130 |
| rno-miR-221-5p | 1.537 | 0.899 | 1.636 | 1.130 | 2.140 | 1.468 | 0.178 | 2.513 | 1.986 | 2.213 | 1.882 | 2.054 | 2.129 | 0.110 | 0.025 | 1.450 | 1.450 | 2.103 |
| rno-miR-678 | 2.948 | 2.400 | 4.194 | 5.553 | 5.890 | 4.197 | 0.803 | 5.924 | 5.828 | 4.983 | 6.432 | 7.240 | 6.081 | 0.371 | 0.043 | 1.449 | 1.449 | 2.100 |
| rno-miR-338-5p | 4.345 | 1.247 | 4.014 | 4.911 | 4.826 | 3.868 | 0.648 | 5.064 | 5.429 | 5.514 | 5.722 | 6.048 | 5.555 | 0.163 | 0.041 | 1.436 | 1.436 | 2.062 |
| rno-miR-298-3p | 1.097 | 0.875 | 1.130 | 1.182 | 1.304 | 1.117 | 0.791 | 1.414 | 1.426 | 1.322 | 1.810 | 2.035 | 1.601 | 0.137 | 0.014 | 1.433 | 1.433 | 2.053 |
| rno-miR-30a-3p | 6.594 | 5.522 | 4.082 | 9.850 | 10.144 | 7.238 | 1.649 | 10.344 | 10.032 | 10.249 | 10.252 | 10.498 | 10.275 | 0.076 | 0.035 | 1.420 | 1.420 | 2.015 |
| rno-miR-7a-5p | 1.467 | 0.994 | 0.978 | 0.829 | 1.178 | 1.089 | 1.656 | 1.515 | 1.752 | 1.880 | 1.434 | 1.147 | 1.545 | 0.128 | 0.027 | 1.419 | 1.419 | 2.013 |
| rno-miR-352 | 6.179 | 5.576 | 4.495 | 6.660 | 7.909 | 6.164 | 1.256 | 8.480 | 8.500 | 8.597 | 8.335 | 9.645 | 8.712 | 0.237 | 0.003 | 1.413 | 1.413 | 1.998 |
| rno-miR-30c-5p | 8.165 | 6.912 | 6.085 | 13.374 | 13.751 | 9.657 | 1.414 | 13.797 | 13.887 | 13.769 | 13.648 | 13.131 | 13.646 | 0.134 | 0.041 | 1.413 | 1.413 | 1.997 |
| rno-miR-3584-5p | 4.426 | 4.283 | 2.989 | 5.297 | 5.769 | 4.553 | 1.652 | 6.554 | 6.378 | 6.244 | 5.811 | 6.966 | 6.391 | 0.189 | 0.007 | 1.404 | 1.404 | 1.970 |
| rno-miR-494-3p | 3.121 | 2.629 | 3.830 | 4.884 | 4.948 | 3.882 | 0.471 | 5.334 | 5.659 | 5.334 | 5.764 | 4.331 | 5.284 | 0.253 | 0.029 | 1.361 | 1.361 | 1.853 |
| rno-mir-877 | 0.982 | 0.690 | 0.769 | 0.819 | 0.792 | 0.810 | 0.788 | 1.158 | 0.927 | 1.275 | 1.147 | 0.999 | 1.101 | 0.062 | 0.006 | 1.359 | 1.359 | 1.846 |
| rno-miR-1188-3p | 1.292 | 1.377 | 1.053 | 1.517 | 1.417 | 1.331 | 0.137 | 1.945 | 2.307 | 1.723 | 1.387 | 1.681 | 1.809 | 0.153 | 0.024 | 1.358 | 1.358 | 1.845 |
| rno-mir-92b | 1.382 | 0.753 | 1.546 | 1.250 | 1.504 | 1.287 | 0.109 | 1.836 | 1.800 | 1.276 | 1.710 | 2.091 | 1.742 | 0.133 | 0.048 | 1.354 | 1.354 | 1.833 |
| rno-mir-182 | 1.699 | 1.200 | 1.820 | 1.577 | 2.001 | 1.659 | 0.158 | 2.312 | 2.546 | 2.051 | 1.707 | 2.605 | 2.244 | 0.166 | 0.026 | 1.352 | 1.352 | 1.829 |
| rno-miR-182 | 2.222 | 2.833 | 1.758 | 1.918 | 2.675 | 2.281 | 0.221 | 2.908 | 3.021 | 3.018 | 3.572 | 2.822 | 3.068 | 0.131 | 0.013 | 1.345 | 1.345 | 1.809 |
| rno-miR-224-5p | 6.766 | 4.363 | 5.613 | 5.811 | 6.195 | 5.749 | 0.871 | 7.530 | 7.437 | 8.079 | 7.783 | 7.772 | 7.720 | 0.112 | 0.001 | 1.343 | 1.343 | 1.803 |
| rno-miR-495 | 0.715 | 1.079 | 1.103 | 0.854 | 1.243 | 0.999 | 1.153 | 1.525 | 1.287 | 1.303 | 1.440 | 1.112 | 1.333 | 0.071 | 0.022 | 1.335 | 1.335 | 1.783 |
| rno-mir-341 | 0.859 | 1.363 | 0.925 | 1.015 | 0.969 | 1.026 | 0.086 | 1.197 | 1.167 | 1.533 | 1.188 | 1.753 | 1.368 | 0.118 | 0.049 | 1.333 | 1.333 | 1.777 |
| rno-mir-135a | 0.821 | 0.792 | 1.158 | 1.129 | 1.335 | 1.047 | 0.091 | 1.460 | 1.329 | 1.261 | 1.623 | 1.285 | 1.391 | 0.067 | 0.024 | 1.329 | 1.329 | 1.766 |
| rno-miR-146b-5p | 5.596 | 4.544 | 6.691 | 7.842 | 8.001 | 6.535 | 1.368 | 8.536 | 8.927 | 8.583 | 8.628 | 8.628 | 8.660 | 0.069 | 0.013 | 1.325 | 1.325 | 1.756 |
| rno-mir-155 | 1.032 | 1.105 | 1.218 | 1.321 | 1.401 | 1.216 | 1.330 | 1.561 | 1.376 | 1.254 | 2.010 | 1.843 | 1.609 | 0.141 | 0.036 | 1.324 | 1.324 | 1.752 |
| rno-let-7f-5p | 9.418 | 7.339 | 6.681 | 10.266 | 11.152 | 8.971 | 1.915 | 11.490 | 11.612 | 11.747 | 11.506 | 12.950 | 11.861 | 0.276 | 0.012 | 1.322 | 1.322 | 1.748 |
| rno-let-7b-3p | 1.410 | 1.200 | 1.529 | 1.564 | 1.200 | 1.381 | 1.878 | 1.987 | 1.541 | 2.408 | 1.689 | 1.456 | 1.816 | 0.173 | 0.051 | 1.315 | 1.315 | 1.730 |
| rno-miR-10a-5p | 6.678 | 5.321 | 5.482 | 6.074 | 6.822 | 6.075 | 1.126 | 8.065 | 7.832 | 7.977 | 7.702 | 8.190 | 7.953 | 0.086 | 0.000 | 1.309 | 1.309 | 1.714 |
| rno-miR-19a-3p | 1.484 | 1.859 | 1.733 | 1.621 | 1.478 | 1.635 | 1.067 | 2.737 | 2.060 | 2.101 | 2.045 | 1.704 | 2.130 | 0.168 | 0.027 | 1.302 | 1.302 | 1.696 |
| rno-mir-496 | 0.945 | 0.844 | 1.061 | 1.003 | 1.112 | 0.993 | 0.162 | 1.319 | 1.090 | 1.273 | 1.262 | 1.517 | 1.292 | 0.068 | 0.007 | 1.301 | 1.301 | 1.693 |
| rno-miR-27b-5p | 4.757 | 4.801 | 3.854 | 4.928 | 5.969 | 4.862 | 0.940 | 6.154 | 6.104 | 6.292 | 6.052 | 6.861 | 6.293 | 0.148 | 0.005 | 1.294 | 1.294 | 1.675 |
| rno-mir-3085 | 1.262 | 1.214 | 1.242 | 0.968 | 1.465 | 1.230 | 0.886 | 1.471 | 1.567 | 1.972 | 1.330 | 1.585 | 1.585 | 0.107 | 0.029 | 1.288 | 1.288 | 1.659 |
| rno-mir-504 | 1.462 | 1.161 | 1.446 | 1.616 | 1.557 | 1.449 | 0.090 | 2.172 | 1.666 | 1.939 | 2.018 | 1.484 | 1.856 | 0.124 | 0.024 | 1.281 | 1.281 | 1.641 |
| rno-mir-26b | 1.439 | 1.688 | 1.587 | 1.486 | 2.030 | 1.646 | 0.099 | 1.934 | 1.928 | 2.369 | 2.461 | 1.832 | 2.105 | 0.129 | 0.025 | 1.279 | 1.279 | 1.635 |
| rno-mir-383 | 1.771 | 1.140 | 1.242 | 1.876 | 1.426 | 1.491 | 0.124 | 2.072 | 1.921 | 1.814 | 1.959 | 1.764 | 1.906 | 0.054 | 0.027 | 1.279 | 1.279 | 1.635 |
| rno-miR-25-3p | 7.382 | 6.477 | 5.227 | 8.088 | 8.842 | 7.203 | 1.414 | 9.141 | 8.914 | 9.385 | 9.403 | 8.996 | 9.168 | 0.099 | 0.015 | 1.273 | 1.273 | 1.620 |
| rno-mir-27b | 1.143 | 1.069 | 0.855 | 1.444 | 1.153 | 1.133 | 1.492 | 1.608 | 1.415 | 1.427 | 1.308 | 1.390 | 1.429 | 0.049 | 0.024 | 1.262 | 1.262 | 1.593 |
| rno-miR-6330 | 0.805 | 0.894 | 0.892 | 0.917 | 0.826 | 0.867 | 0.090 | 1.132 | 0.922 | 1.204 | 0.931 | 1.239 | 1.086 | 0.067 | 0.015 | 1.252 | 1.252 | 1.569 |
| rno-miR-449a-5p | 0.904 | 1.439 | 1.237 | 1.271 | 1.301 | 1.231 | 0.105 | 1.342 | 1.562 | 1.811 | 1.364 | 1.612 | 1.538 | 0.087 | 0.038 | 1.250 | 1.250 | 1.563 |
| rno-miR-146a-5p | 8.247 | 7.606 | 8.694 | 10.253 | 11.155 | 9.191 | 1.928 | 11.112 | 11.402 | 11.437 | 11.378 | 11.811 | 11.428 | 0.112 | 0.010 | 1.243 | 1.243 | 1.546 |
| rno-miR-489-3p | 1.029 | 0.776 | 1.059 | 0.894 | 1.047 | 0.961 | 1.989 | 1.342 | 1.140 | 1.015 | 1.363 | 1.105 | 1.193 | 0.068 | 0.029 | 1.242 | 1.242 | 1.542 |
| rno-mir-191b | 1.451 | 1.123 | 1.207 | 1.560 | 1.217 | 1.311 | 0.106 | 1.450 | 1.698 | 1.547 | 1.462 | 1.836 | 1.598 | 0.074 | 0.032 | 1.219 | 1.219 | 1.486 |
| rno-mir-300 | 0.891 | 0.892 | 0.956 | 0.894 | 1.143 | 0.955 | 0.106 | 1.217 | 1.265 | 1.066 | 1.240 | 1.009 | 1.159 | 0.051 | 0.020 | 1.214 | 1.214 | 1.473 |
| rno-mir-103-2 | 1.077 | 1.003 | 0.838 | 0.870 | 0.871 | 0.932 | 0.045 | 1.013 | 1.247 | 1.200 | 0.960 | 1.179 | 1.119 | 0.056 | 0.033 | 1.201 | 1.201 | 1.443 |
| rno-miR-199a-3p | 10.078 | 9.137 | 8.564 | 10.378 | 10.937 | 9.819 | 2.114 | 11.480 | 11.295 | 11.731 | 11.438 | 11.496 | 11.488 | 0.070 | 0.005 | 1.170 | 1.170 | 1.369 |
| rno-miR-20b-5p | 6.444 | 5.878 | 5.959 | 7.396 | 7.789 | 6.693 | 0.831 | 7.719 | 7.712 | 7.817 | 8.021 | 7.249 | 7.704 | 0.127 | 0.037 | 1.151 | 1.151 | 1.325 |
| rno-miR-6216 | 10.314 | 10.931 | 10.589 | 12.680 | 12.661 | 11.435 | 1.197 | 12.944 | 13.178 | 12.861 | 12.701 | 13.679 | 13.073 | 0.170 | 0.016 | 1.143 | 1.143 | 1.307 |
| rno-miR-1-3p | 11.523 | 10.778 | 11.276 | 12.309 | 12.925 | 11.762 | 0.434 | 13.197 | 13.198 | 13.414 | 13.257 | 13.915 | 13.396 | 0.136 | 0.004 | 1.139 | 1.139 | 1.297 |
| rno-miR-28-3p | 7.826 | 7.163 | 6.773 | 8.400 | 8.556 | 7.744 | 1.007 | 8.847 | 8.649 | 8.898 | 8.828 | 8.806 | 8.806 | 0.042 | 0.016 | 1.137 | 1.137 | 1.293 |
| rno-miR-15b-5p | 9.555 | 8.723 | 9.040 | 8.977 | 9.185 | 9.096 | 0.403 | 10.063 | 9.708 | 10.382 | 10.455 | 10.442 | 10.210 | 0.144 | 0.001 | 1.122 | 1.122 | 1.260 |
| rno-miR-27b-3p | 10.915 | 10.166 | 10.089 | 12.259 | 12.475 | 11.181 | 0.603 | 12.634 | 12.559 | 12.738 | 12.620 | 12.047 | 12.519 | 0.122 | 0.033 | 1.120 | 1.120 | 1.254 |
| rno-miR-128-3p | 8.170 | 7.083 | 7.521 | 6.889 | 7.569 | 7.446 | 0.954 | 8.051 | 7.913 | 8.455 | 8.533 | 7.747 | 8.140 | 0.153 | 0.033 | 1.093 | 1.093 | 1.195 |
| rno-miR-221-3p | 9.598 | 8.920 | 8.811 | 9.262 | 9.444 | 9.207 | 0.452 | 9.984 | 10.096 | 10.188 | 10.156 | 9.889 | 10.063 | 0.056 | 0.001 | 1.093 | 1.093 | 1.195 |
| rno-miR-674-3p | 7.020 | 6.229 | 7.194 | 6.345 | 7.055 | 6.769 | 0.598 | 7.010 | 7.471 | 7.334 | 7.328 | 7.775 | 7.384 | 0.124 | 0.031 | 1.091 | 1.091 | 1.190 |
| rno-let-7i-5p | 12.580 | 11.339 | 10.957 | 11.859 | 12.355 | 11.818 | 1.215 | 12.693 | 12.716 | 12.962 | 12.645 | 13.375 | 12.878 | 0.136 | 0.013 | 1.090 | 1.090 | 1.187 |
| rno-miR-150-5p | 10.532 | 10.696 | 11.102 | 11.991 | 12.292 | 11.322 | 0.330 | 12.085 | 12.085 | 12.102 | 12.266 | 12.572 | 12.222 | 0.094 | 0.038 | 1.079 | 1.079 | 1.165 |
| rno-miR-26a-5p | 13.567 | 13.204 | 12.866 | 14.632 | 14.854 | 13.825 | 0.686 | 14.840 | 14.905 | 14.870 | 14.901 | 14.593 | 14.822 | 0.058 | 0.036 | 1.072 | 1.072 | 1.149 |
| rno-miR-92a-3p | 10.319 | 10.171 | 9.719 | 10.565 | 11.241 | 10.403 | 0.864 | 11.044 | 11.046 | 11.228 | 11.030 | 11.261 | 11.122 | 0.050 | 0.023 | 1.069 | 1.069 | 1.143 |
| rno-miR-29b-2-5p | 7.137 | 6.958 | 6.339 | 6.899 | 7.141 | 6.895 | 0.849 | 7.092 | 7.327 | 7.323 | 7.404 | 7.706 | 7.371 | 0.099 | 0.028 | 1.069 | 1.069 | 1.143 |
| rno-miR-195-5p | 11.880 | 11.448 | 10.765 | 12.192 | 12.113 | 11.679 | 1.145 | 12.522 | 12.239 | 12.481 | 12.598 | 12.411 | 12.450 | 0.061 | 0.021 | 1.066 | 1.066 | 1.136 |
| rno-miR-126a-3p | 12.450 | 12.348 | 12.219 | 13.624 | 13.684 | 12.865 | 0.394 | 13.797 | 13.836 | 13.785 | 13.718 | 13.400 | 13.707 | 0.079 | 0.036 | 1.065 | 1.065 | 1.135 |
| rno-miR-222-3p | 9.382 | 9.204 | 9.572 | 9.559 | 9.284 | 9.400 | 0.846 | 9.919 | 9.876 | 10.054 | 10.134 | 9.689 | 9.934 | 0.077 | 0.001 | 1.057 | 1.057 | 1.117 |
| rno-let-7a-5p | 13.802 | 13.596 | 13.649 | 13.540 | 13.900 | 13.698 | 1.015 | 14.013 | 14.274 | 14.306 | 14.220 | 14.701 | 14.303 | 0.112 | 0.002 | 1.044 | 1.044 | 1.090 |
| rno-mir-361 | 8.636 | 9.136 | 8.823 | 8.886 | 8.903 | 8.877 | 1.138 | 9.160 | 9.112 | 9.181 | 9.292 | 9.531 | 9.255 | 0.075 | 0.009 | 1.043 | 1.043 | 1.087 |
| rno-miR-125a-5p | 12.838 | 12.818 | 12.815 | 12.900 | 13.108 | 12.896 | 0.950 | 13.248 | 13.182 | 13.355 | 13.446 | 13.566 | 13.359 | 0.068 | 0.001 | 1.036 | 1.036 | 1.073 |
| rno-miR-125b-5p | 13.785 | 13.593 | 13.824 | 13.599 | 13.938 | 13.748 | 0.209 | 13.880 | 14.007 | 14.000 | 14.000 | 14.380 | 14.054 | 0.085 | 0.022 | 1.022 | 1.022 | 1.045 |
| rno-let-7c-5p | 14.868 | 14.747 | 14.802 | 14.818 | 14.947 | 14.837 | 0.261 | 14.920 | 15.359 | 15.136 | 15.099 | 15.155 | 15.134 | 0.070 | 0.005 | 1.020 | 1.020 | 1.040 |
| AFFX-r2-P1-c3-cre-5 | 15.113 | 15.077 | 15.086 | 15.223 | 15.229 | 15.146 | 0.079 | 15.295 | 15.172 | 15.230 | 15.288 | 15.393 | 15.276 | 0.037 | 0.031 | 1.009 | 1.009 | 1.017 |
| rno-miR-24-3p | 14.994 | 14.860 | 14.713 | 14.818 | 14.780 | 14.833 | 0.083 | 14.583 | 14.656 | 14.588 | 14.578 | 14.233 | 14.528 | 0.075 | 0.009 | 0.979 | -1.021 | -1.042 |
| rno-miR-191a-5p | 14.138 | 14.250 | 14.325 | 13.777 | 13.578 | 14.014 | 0.218 | 13.630 | 13.737 | 13.661 | 13.647 | 13.550 | 13.645 | 0.030 | 0.036 | 0.974 | -1.027 | -1.055 |
| rno-miR-139-5p | 11.014 | 10.975 | 10.734 | 10.645 | 10.523 | 10.778 | 0.771 | 10.443 | 10.362 | 10.202 | 10.464 | 10.220 | 10.338 | 0.055 | 0.004 | 0.959 | -1.043 | -1.087 |
| rno-miR-181a-5p | 12.417 | 12.425 | 12.091 | 12.264 | 12.220 | 12.283 | 0.363 | 11.852 | 11.832 | 11.824 | 11.913 | 11.426 | 11.769 | 0.087 | 0.001 | 0.958 | -1.044 | -1.089 |
| rno-miR-378a-3p | 14.198 | 14.297 | 14.467 | 13.700 | 13.539 | 14.040 | 0.433 | 13.395 | 13.321 | 13.453 | 13.454 | 13.456 | 13.416 | 0.026 | 0.009 | 0.956 | -1.047 | -1.095 |
| rno-miR-107-3p | 13.744 | 13.780 | 13.700 | 12.981 | 12.907 | 13.422 | 0.229 | 12.880 | 12.777 | 12.891 | 12.901 | 12.591 | 12.808 | 0.059 | 0.017 | 0.954 | -1.048 | -1.098 |
| rno-miR-151-3p | 10.603 | 10.688 | 10.731 | 10.003 | 9.829 | 10.371 | 0.742 | 9.943 | 9.599 | 9.713 | 9.783 | 10.209 | 9.849 | 0.106 | 0.043 | 0.950 | -1.053 | -1.109 |
| rno-miR-103-3p | 13.965 | 14.050 | 13.941 | 13.436 | 13.169 | 13.712 | 0.806 | 13.073 | 12.970 | 13.025 | 13.148 | 12.866 | 13.016 | 0.048 | 0.005 | 0.949 | -1.053 | -1.110 |
| rno-miR-24-2-5p | 10.996 | 10.958 | 10.614 | 10.302 | 10.413 | 10.657 | 0.735 | 10.277 | 10.003 | 9.990 | 10.062 | 10.213 | 10.109 | 0.058 | 0.007 | 0.949 | -1.054 | -1.111 |
| rno-mir-139 | 7.401 | 7.725 | 7.440 | 7.119 | 7.258 | 7.388 | 0.779 | 6.986 | 7.029 | 6.973 | 7.049 | 6.920 | 6.991 | 0.023 | 0.005 | 0.946 | -1.057 | -1.117 |
| rno-miR-99b-5p | 13.106 | 13.185 | 13.041 | 12.410 | 11.877 | 12.724 | 1.271 | 12.033 | 11.979 | 11.926 | 12.181 | 11.555 | 11.935 | 0.104 | 0.020 | 0.938 | -1.066 | -1.137 |
| rno-miR-17-5p | 11.551 | 11.829 | 11.609 | 11.081 | 10.904 | 11.395 | 0.374 | 10.759 | 10.690 | 10.795 | 10.885 | 10.064 | 10.639 | 0.147 | 0.010 | 0.934 | -1.071 | -1.147 |
| rno-miR-652-3p | 11.852 | 12.309 | 11.937 | 10.920 | 10.696 | 11.543 | 0.240 | 10.750 | 10.661 | 10.620 | 10.557 | 11.085 | 10.735 | 0.093 | 0.038 | 0.930 | -1.075 | -1.156 |
| rno-miR-351-5p | 10.937 | 11.077 | 11.082 | 10.052 | 9.806 | 10.591 | 0.356 | 9.899 | 9.760 | 9.805 | 9.945 | 9.169 | 9.716 | 0.141 | 0.022 | 0.917 | -1.090 | -1.188 |
| rno-miR-532-5p | 9.268 | 8.911 | 8.754 | 8.350 | 8.204 | 8.697 | 0.499 | 8.106 | 7.903 | 8.185 | 7.999 | 7.576 | 7.954 | 0.106 | 0.010 | 0.915 | -1.093 | -1.196 |
| rno-mir-351 | 6.992 | 6.121 | 6.612 | 5.959 | 5.871 | 6.311 | 0.594 | 5.952 | 5.752 | 5.635 | 5.976 | 5.514 | 5.766 | 0.089 | 0.046 | 0.914 | -1.095 | -1.198 |
| rno-miR-497-5p | 10.018 | 9.933 | 9.547 | 9.627 | 9.140 | 9.653 | 0.807 | 9.007 | 8.561 | 8.902 | 9.009 | 8.247 | 8.745 | 0.149 | 0.003 | 0.906 | -1.104 | -1.218 |
| rno-miR-140-3p | 12.118 | 12.455 | 12.374 | 11.556 | 11.160 | 11.933 | 0.572 | 11.012 | 10.409 | 10.998 | 11.196 | 10.360 | 10.795 | 0.171 | 0.006 | 0.905 | -1.105 | -1.222 |
| rno-mir-378a | 3.467 | 3.142 | 3.136 | 2.830 | 2.882 | 3.091 | 2.092 | 2.822 | 2.664 | 2.886 | 2.816 | 2.772 | 2.792 | 0.037 | 0.036 | 0.903 | -1.107 | -1.226 |
| rno-miR-378a-5p | 10.903 | 11.031 | 11.260 | 10.282 | 10.450 | 10.785 | 1.819 | 9.950 | 9.908 | 9.789 | 9.799 | 8.992 | 9.688 | 0.177 | 0.003 | 0.898 | -1.113 | -1.239 |
| rno-miR-214-3p | 11.723 | 12.161 | 12.185 | 10.722 | 10.296 | 11.417 | 0.321 | 10.198 | 9.751 | 10.070 | 10.127 | 11.046 | 10.238 | 0.216 | 0.029 | 0.897 | -1.115 | -1.244 |
| rno-miR-93-5p | 11.365 | 11.540 | 11.180 | 10.539 | 10.285 | 10.982 | 0.321 | 10.083 | 9.737 | 9.832 | 10.122 | 9.374 | 9.829 | 0.135 | 0.003 | 0.895 | -1.117 | -1.248 |
| rno-miR-324-5p | 9.417 | 9.446 | 9.177 | 8.520 | 8.192 | 8.950 | 0.533 | 7.962 | 7.994 | 7.903 | 8.526 | 7.633 | 8.004 | 0.145 | 0.012 | 0.894 | -1.118 | -1.250 |
| rno-miR-125b-2-3p | 10.018 | 9.909 | 9.600 | 9.053 | 8.837 | 9.484 | 0.261 | 8.662 | 8.391 | 8.498 | 8.598 | 8.096 | 8.449 | 0.100 | 0.004 | 0.891 | -1.122 | -1.260 |
| rno-miR-500-3p | 9.605 | 9.899 | 9.808 | 8.735 | 8.323 | 9.274 | 0.266 | 8.243 | 8.150 | 8.285 | 8.325 | 8.266 | 8.254 | 0.029 | 0.012 | 0.890 | -1.124 | -1.263 |
| rno-miR-652-5p | 7.086 | 6.433 | 6.986 | 5.919 | 5.852 | 6.455 | 0.718 | 5.714 | 6.122 | 5.392 | 5.655 | 5.819 | 5.740 | 0.119 | 0.036 | 0.889 | -1.125 | -1.265 |
| rno-mir-10a | 1.188 | 1.120 | 0.979 | 1.093 | 0.993 | 1.075 | 1.280 | 1.021 | 0.934 | 0.869 | 0.926 | 1.027 | 0.955 | 0.030 | 0.043 | 0.889 | -1.125 | -1.265 |
| rno-mir-423 | 7.294 | 7.660 | 7.082 | 6.471 | 6.317 | 6.965 | 1.399 | 6.233 | 6.371 | 6.252 | 6.280 | 5.670 | 6.161 | 0.125 | 0.021 | 0.885 | -1.130 | -1.278 |
| rno-miR-542-5p | 7.876 | 7.565 | 7.548 | 6.809 | 6.666 | 7.293 | 0.242 | 6.674 | 6.594 | 6.437 | 6.148 | 6.036 | 6.378 | 0.124 | 0.009 | 0.875 | -1.144 | -1.308 |
| rno-miR-106b-3p | 8.936 | 8.807 | 8.980 | 7.705 | 7.446 | 8.375 | 0.372 | 7.433 | 7.360 | 7.410 | 7.644 | 6.688 | 7.307 | 0.162 | 0.020 | 0.872 | -1.146 | -1.314 |
| rno-miR-423-3p | 10.050 | 10.210 | 10.029 | 8.927 | 8.751 | 9.594 | 0.417 | 8.562 | 8.584 | 8.558 | 8.240 | 7.756 | 8.340 | 0.159 | 0.007 | 0.869 | -1.150 | -1.323 |
| rno-miR-503-5p | 9.711 | 9.715 | 9.450 | 8.805 | 8.473 | 9.231 | 0.280 | 8.311 | 7.944 | 8.124 | 7.872 | 7.594 | 7.969 | 0.121 | 0.002 | 0.863 | -1.158 | -1.342 |
| rno-miR-345-3p | 9.004 | 8.945 | 8.449 | 7.590 | 7.780 | 8.354 | 0.330 | 7.187 | 6.923 | 7.235 | 7.280 | 7.250 | 7.175 | 0.065 | 0.004 | 0.859 | -1.164 | -1.355 |
| rno-miR-339-5p | 9.448 | 9.445 | 9.252 | 8.743 | 8.523 | 9.082 | 0.288 | 7.932 | 8.262 | 7.772 | 7.847 | 6.931 | 7.749 | 0.221 | 0.002 | 0.853 | -1.172 | -1.374 |
| rno-miR-324-3p | 8.638 | 9.147 | 8.937 | 7.593 | 7.558 | 8.375 | 0.307 | 7.274 | 7.332 | 7.266 | 7.456 | 6.304 | 7.127 | 0.208 | 0.013 | 0.851 | -1.175 | -1.381 |
| rno-miR-345-5p | 9.219 | 9.044 | 8.505 | 8.225 | 8.604 | 8.719 | 0.267 | 7.689 | 7.846 | 7.319 | 7.469 | 6.688 | 7.402 | 0.200 | 0.001 | 0.849 | -1.178 | -1.388 |
| rno-miR-339-3p | 9.577 | 9.732 | 9.624 | 8.482 | 8.209 | 9.125 | 0.264 | 7.849 | 7.713 | 7.680 | 7.850 | 7.630 | 7.744 | 0.045 | 0.003 | 0.849 | -1.178 | -1.388 |
| rno-miR-6324 | 6.761 | 6.318 | 6.440 | 5.648 | 5.157 | 6.065 | 0.777 | 5.104 | 5.366 | 4.730 | 5.320 | 5.214 | 5.147 | 0.114 | 0.019 | 0.849 | -1.178 | -1.389 |
| rno-miR-25-5p | 8.857 | 8.814 | 9.322 | 7.190 | 6.847 | 8.206 | 0.634 | 6.755 | 6.810 | 6.976 | 6.832 | 7.440 | 6.963 | 0.125 | 0.041 | 0.848 | -1.179 | -1.389 |
| rno-miR-139-3p | 7.984 | 8.102 | 7.371 | 6.749 | 6.623 | 7.366 | 0.436 | 6.162 | 6.375 | 6.055 | 6.083 | 6.319 | 6.199 | 0.064 | 0.006 | 0.842 | -1.188 | -1.412 |
| rno-mir-1298 | 1.029 | 1.035 | 1.299 | 1.092 | 1.162 | 1.123 | 1.486 | 0.918 | 0.985 | 0.837 | 1.123 | 0.843 | 0.941 | 0.053 | 0.037 | 0.838 | -1.194 | -1.425 |
| rno-miR-210-3p | 11.358 | 11.764 | 10.880 | 10.550 | 9.984 | 10.907 | 2.316 | 9.340 | 9.187 | 9.108 | 9.215 | 8.598 | 9.090 | 0.128 | 0.001 | 0.833 | -1.200 | -1.440 |
| rno-miR-346 | 8.280 | 8.235 | 7.837 | 8.017 | 7.147 | 7.903 | 0.750 | 6.861 | 6.298 | 6.722 | 7.160 | 5.827 | 6.574 | 0.232 | 0.003 | 0.832 | -1.202 | -1.446 |
| rno-miR-336-3p | 1.049 | 0.942 | 0.891 | 1.007 | 1.208 | 1.019 | 1.629 | 0.931 | 0.850 | 0.785 | 0.707 | 0.942 | 0.843 | 0.045 | 0.036 | 0.827 | -1.209 | -1.462 |
| rno-let-7f-2 | 1.060 | 1.026 | 0.972 | 0.894 | 1.159 | 1.022 | 0.047 | 0.781 | 0.701 | 0.845 | 0.924 | 0.971 | 0.844 | 0.048 | 0.027 | 0.826 | -1.211 | -1.465 |
| rno-miR-421-3p | 1.143 | 1.438 | 1.314 | 1.071 | 1.120 | 1.217 | 0.071 | 1.024 | 0.927 | 0.934 | 0.907 | 1.224 | 1.003 | 0.059 | 0.046 | 0.824 | -1.214 | -1.473 |
| rno-mir-433 | 1.816 | 1.614 | 1.335 | 1.533 | 1.647 | 1.589 | 0.112 | 1.316 | 1.386 | 1.114 | 1.547 | 1.175 | 1.308 | 0.077 | 0.034 | 0.823 | -1.215 | -1.476 |
| rno-miR-874-3p | 6.855 | 6.675 | 6.545 | 6.007 | 5.406 | 6.298 | 1.125 | 5.502 | 5.310 | 5.484 | 5.655 | 3.748 | 5.140 | 0.352 | 0.030 | 0.816 | -1.225 | -1.501 |
| rno-miR-143-5p | 7.534 | 7.108 | 5.810 | 6.451 | 6.394 | 6.660 | 0.280 | 6.012 | 5.671 | 5.187 | 5.850 | 4.449 | 5.434 | 0.282 | 0.018 | 0.816 | -1.226 | -1.502 |
| rno-miR-210-5p | 6.881 | 6.706 | 6.535 | 5.735 | 5.678 | 6.307 | 0.274 | 5.458 | 5.766 | 5.525 | 5.452 | 3.464 | 5.133 | 0.421 | 0.044 | 0.814 | -1.229 | -1.510 |
| rno-miR-490-3p | 8.861 | 8.715 | 7.736 | 7.521 | 7.191 | 8.005 | 0.487 | 6.655 | 6.815 | 6.650 | 6.751 | 5.500 | 6.475 | 0.245 | 0.006 | 0.809 | -1.236 | -1.529 |
| rno-miR-17-1-3p | 8.006 | 8.002 | 7.470 | 6.799 | 6.487 | 7.353 | 0.339 | 5.881 | 6.115 | 6.241 | 6.343 | 4.942 | 5.904 | 0.253 | 0.007 | 0.803 | -1.245 | -1.551 |
| rno-miR-370-5p | 1.002 | 1.137 | 0.976 | 0.961 | 1.003 | 1.016 | 1.508 | 0.775 | 0.811 | 0.717 | 1.019 | 0.756 | 0.816 | 0.053 | 0.012 | 0.803 | -1.245 | -1.551 |
| rno-mir-101a | 1.354 | 1.204 | 1.043 | 1.381 | 1.592 | 1.315 | 0.096 | 1.021 | 1.147 | 0.934 | 0.924 | 1.229 | 1.051 | 0.060 | 0.043 | 0.800 | -1.251 | -1.564 |
| rno-mir-490 | 3.600 | 3.401 | 2.700 | 2.725 | 2.776 | 3.040 | 0.430 | 2.584 | 2.717 | 2.554 | 2.159 | 2.050 | 2.413 | 0.130 | 0.026 | 0.794 | -1.260 | -1.588 |
| rno-mir-880 | 1.394 | 1.231 | 1.531 | 1.461 | 0.896 | 1.303 | 0.435 | 1.005 | 1.033 | 0.974 | 1.010 | 1.139 | 1.032 | 0.028 | 0.049 | 0.792 | -1.262 | -1.593 |
| rno-miR-138-5p | 8.030 | 8.128 | 9.550 | 6.661 | 6.614 | 7.797 | 1.575 | 5.909 | 6.212 | 6.469 | 6.507 | 5.740 | 6.167 | 0.151 | 0.020 | 0.791 | -1.264 | -1.598 |
| rno-mir-409a | 1.493 | 1.414 | 1.654 | 1.654 | 1.391 | 1.521 | 1.523 | 1.260 | 0.941 | 1.124 | 1.339 | 1.353 | 1.203 | 0.077 | 0.011 | 0.791 | -1.265 | -1.599 |
| rno-miR-330-3p | 6.781 | 6.890 | 6.605 | 4.743 | 5.116 | 6.027 | 1.105 | 4.715 | 4.830 | 3.717 | 5.013 | 5.298 | 4.715 | 0.268 | 0.038 | 0.782 | -1.278 | -1.634 |
| rno-mir-3579 | 1.287 | 1.003 | 1.245 | 1.244 | 0.880 | 1.132 | 1.194 | 1.047 | 0.925 | 0.689 | 0.975 | 0.743 | 0.876 | 0.069 | 0.042 | 0.774 | -1.293 | -1.671 |
| rno-mir-6333 | 1.282 | 1.434 | 1.646 | 1.102 | 1.179 | 1.328 | 0.096 | 0.979 | 1.024 | 1.229 | 0.707 | 1.108 | 1.009 | 0.087 | 0.040 | 0.760 | -1.316 | -1.732 |
| rno-mir-125b-2 | 2.490 | 3.164 | 2.292 | 1.941 | 1.942 | 2.366 | 0.294 | 1.877 | 1.797 | 1.589 | 1.860 | 1.634 | 1.751 | 0.059 | 0.030 | 0.740 | -1.351 | -1.825 |
| rno-miR-107-5p | 1.511 | 1.149 | 1.676 | 1.390 | 1.211 | 1.388 | 0.283 | 1.055 | 1.005 | 1.159 | 1.181 | 0.715 | 1.023 | 0.083 | 0.021 | 0.737 | -1.356 | -1.840 |
| rno-mir-196b | 1.195 | 1.279 | 1.884 | 1.044 | 1.266 | 1.334 | 0.116 | 0.918 | 0.981 | 0.801 | 1.139 | 0.988 | 0.965 | 0.055 | 0.044 | 0.724 | -1.381 | -1.908 |
| rno-mir-489 | 1.030 | 1.373 | 1.380 | 1.221 | 1.225 | 1.246 | 0.107 | 0.901 | 0.752 | 0.749 | 0.897 | 1.179 | 0.896 | 0.078 | 0.008 | 0.719 | -1.391 | -1.935 |
| rno-mir-673 | 1.646 | 1.216 | 1.547 | 1.070 | 1.512 | 1.398 | 0.092 | 0.824 | 1.115 | 1.147 | 0.882 | 1.018 | 0.997 | 0.063 | 0.013 | 0.713 | -1.402 | -1.966 |
| rno-mir-181b-1 | 1.044 | 1.369 | 1.793 | 1.664 | 1.035 | 1.381 | 0.127 | 1.004 | 1.148 | 0.882 | 1.059 | 0.752 | 0.969 | 0.069 | 0.042 | 0.702 | -1.425 | -2.032 |
| rno-miR-764-3p | 1.528 | 1.132 | 1.555 | 1.551 | 1.018 | 1.357 | 0.130 | 0.781 | 1.135 | 0.939 | 0.784 | 1.036 | 0.935 | 0.070 | 0.015 | 0.689 | -1.451 | -2.106 |
| rno-miR-463-3p | 0.948 | 1.380 | 1.535 | 1.369 | 1.003 | 1.247 | 0.112 | 0.919 | 0.768 | 0.760 | 0.871 | 0.918 | 0.847 | 0.035 | 0.010 | 0.679 | -1.472 | -2.167 |
| rno-miR-667-3p | 3.234 | 2.585 | 2.524 | 2.457 | 3.667 | 2.893 | 0.426 | 1.521 | 1.937 | 1.652 | 2.917 | 1.764 | 1.958 | 0.249 | 0.027 | 0.677 | -1.478 | -2.183 |
| rno-miR-344i | 1.297 | 1.434 | 1.474 | 1.132 | 0.900 | 1.247 | 0.425 | 0.664 | 1.254 | 0.938 | 0.629 | 0.711 | 0.839 | 0.117 | 0.032 | 0.673 | -1.486 | -2.209 |
| rno-miR-181c-3p | 7.032 | 6.950 | 5.988 | 6.212 | 5.822 | 6.401 | 1.228 | 4.612 | 5.105 | 4.534 | 4.641 | 1.738 | 4.126 | 0.605 | 0.008 | 0.645 | -1.551 | -2.406 |

| **Supplemental Table 2. Differentially expressed miRNA that were significant (*p*<0.05) in ZL-Rap vs. ZL-C rat hearts**. | | | | | | | | | | | | | | | | | |
| --- | --- | --- | --- | --- | --- | --- | --- | --- | --- | --- | --- | --- | --- | --- | --- | --- | --- |
|  | ZL-C | ZL-C | ZL-C | ZL-C | ZL-C | AVG | STE | ZL-Rap | ZL-Rap | ZL-Rap | ZL-Rap | AVG | STE | p-val | ZL-Rap/ ZL-C | Log Ratio | Fold Diff |
| rno-miR-31a-3p | 1.318 | 1.312 | 1.034 | 1.444 | 1.629 | 1.347 | 0.097 | 5.058 | 5.860 | 4.156 | 2.747 | 4.455 | 0.667 | 0.001 | 3.307 | 3.307 | 10.934 |
| rno-miR-434-3p | 1.954 | 1.021 | 1.197 | 2.565 | 3.260 | 2.000 | 0.419 | 7.017 | 7.095 | 5.120 | 6.528 | 6.440 | 0.458 | 0.000 | 3.221 | 3.221 | 10.373 |
| rno-miR-411-5p | 1.102 | 0.898 | 1.293 | 0.895 | 1.003 | 1.038 | 0.074 | 3.107 | 3.978 | 2.685 | 2.017 | 2.947 | 0.411 | 0.001 | 2.838 | 2.838 | 8.053 |
| rno-miR-204-5p | 1.143 | 0.887 | 1.294 | 1.458 | 2.048 | 1.366 | 0.195 | 4.316 | 4.356 | 3.521 | 3.182 | 3.844 | 0.293 | 0.000 | 2.814 | 2.814 | 7.920 |
| rno-miR-7a-1-3p | 1.144 | 1.156 | 0.976 | 2.555 | 3.566 | 1.879 | 0.509 | 5.432 | 5.956 | 4.600 | 4.873 | 5.215 | 0.302 | 0.001 | 2.775 | 2.775 | 7.700 |
| rno-miR-450a-5p | 0.911 | 1.176 | 1.624 | 1.522 | 1.405 | 1.327 | 0.128 | 3.934 | 3.560 | 4.463 | 2.250 | 3.552 | 0.472 | 0.001 | 2.676 | 2.676 | 7.158 |
| rno-miR-362-3p | 1.029 | 1.003 | 1.057 | 1.014 | 1.871 | 1.195 | 0.169 | 3.743 | 4.083 | 2.795 | 2.088 | 3.177 | 0.454 | 0.003 | 2.659 | 2.659 | 7.071 |
| rno-miR-455-5p | 1.063 | 1.257 | 0.921 | 1.188 | 1.694 | 1.224 | 0.131 | 4.148 | 3.995 | 3.414 | 1.454 | 3.253 | 0.620 | 0.009 | 2.656 | 2.656 | 7.057 |
| rno-miR-374-5p | 1.476 | 1.140 | 1.747 | 1.813 | 2.874 | 1.810 | 0.291 | 5.016 | 4.726 | 4.418 | 4.472 | 4.658 | 0.137 | 0.000 | 2.573 | 2.573 | 6.622 |
| rno-miR-329-3p | 2.001 | 1.535 | 1.567 | 1.676 | 2.732 | 1.902 | 0.223 | 5.470 | 5.827 | 3.180 | 4.281 | 4.689 | 0.602 | 0.002 | 2.465 | 2.465 | 6.077 |
| rno-miR-872-5p | 1.191 | 0.994 | 0.725 | 0.900 | 1.262 | 1.014 | 0.097 | 3.092 | 2.493 | 2.739 | 1.677 | 2.500 | 0.301 | 0.001 | 2.465 | 2.465 | 6.074 |
| rno-miR-21-5p | 1.051 | 1.012 | 0.984 | 5.808 | 7.072 | 3.185 | 1.344 | 7.600 | 7.668 | 7.664 | 7.742 | 7.669 | 0.029 | 0.022 | 2.407 | 2.407 | 5.796 |
| rno-miR-26b-5p | 1.382 | 1.204 | 1.169 | 5.953 | 6.958 | 3.333 | 1.285 | 7.721 | 7.923 | 7.897 | 7.975 | 7.879 | 0.055 | 0.017 | 2.364 | 2.364 | 5.588 |
| rno-miR-200b-3p | 2.117 | 0.882 | 1.049 | 1.740 | 3.295 | 1.817 | 0.433 | 4.506 | 4.814 | 2.562 | 5.001 | 4.221 | 0.562 | 0.011 | 2.323 | 2.323 | 5.398 |
| rno-miR-505-3p | 2.067 | 1.021 | 1.113 | 1.659 | 3.374 | 1.847 | 0.426 | 4.825 | 4.554 | 4.053 | 3.588 | 4.255 | 0.274 | 0.003 | 2.304 | 2.304 | 5.308 |
| rno-miR-30e-3p | 1.407 | 1.001 | 1.099 | 7.542 | 8.062 | 3.822 | 1.628 | 8.780 | 8.832 | 8.237 | 8.788 | 8.659 | 0.141 | 0.035 | 2.265 | 2.265 | 5.132 |
| rno-miR-451-5p | 1.294 | 1.696 | 1.119 | 4.189 | 4.864 | 2.632 | 0.786 | 6.174 | 6.849 | 5.973 | 4.803 | 5.950 | 0.426 | 0.011 | 2.260 | 2.260 | 5.109 |
| rno-miR-101b-3p | 1.622 | 1.304 | 1.164 | 2.758 | 3.691 | 2.108 | 0.485 | 4.975 | 5.339 | 5.079 | 3.611 | 4.751 | 0.388 | 0.005 | 2.254 | 2.254 | 5.080 |
| rno-miR-665 | 1.079 | 0.961 | 1.463 | 1.022 | 1.185 | 1.142 | 0.088 | 3.402 | 3.236 | 1.526 | 2.122 | 2.571 | 0.450 | 0.010 | 2.252 | 2.252 | 5.071 |
| rno-miR-409a-5p | 1.559 | 0.761 | 1.667 | 1.124 | 1.008 | 1.224 | 0.170 | 3.699 | 3.015 | 1.693 | 2.517 | 2.731 | 0.422 | 0.009 | 2.232 | 2.232 | 4.980 |
| rno-miR-301a-3p | 1.063 | 1.179 | 1.508 | 3.498 | 5.109 | 2.472 | 0.793 | 5.859 | 6.437 | 5.310 | 4.230 | 5.459 | 0.470 | 0.019 | 2.209 | 2.209 | 4.879 |
| rno-miR-382-5p | 2.998 | 0.887 | 2.511 | 1.756 | 3.723 | 2.375 | 0.491 | 5.628 | 5.543 | 4.101 | 5.540 | 5.203 | 0.368 | 0.003 | 2.191 | 2.191 | 4.799 |
| rno-miR-155-5p | 2.088 | 1.095 | 1.622 | 4.637 | 5.950 | 3.078 | 0.941 | 6.779 | 6.624 | 6.543 | 6.973 | 6.730 | 0.095 | 0.011 | 2.186 | 2.186 | 4.779 |
| rno-miR-9a-3p | 1.448 | 1.313 | 0.995 | 0.993 | 1.647 | 1.279 | 0.128 | 2.362 | 4.343 | 2.032 | 2.432 | 2.792 | 0.524 | 0.017 | 2.182 | 2.182 | 4.762 |
| rno-miR-29b-3p | 1.238 | 1.198 | 1.017 | 6.931 | 7.394 | 3.556 | 1.475 | 8.130 | 8.171 | 8.049 | 6.672 | 7.755 | 0.362 | 0.043 | 2.181 | 2.181 | 4.757 |
| rno-miR-19a-3p | 1.484 | 1.859 | 1.733 | 1.621 | 1.478 | 1.635 | 0.073 | 3.616 | 4.353 | 3.692 | 2.557 | 3.555 | 0.372 | 0.001 | 2.174 | 2.174 | 4.726 |
| rno-miR-328b-3p | 1.591 | 1.202 | 1.227 | 1.548 | 3.430 | 1.800 | 0.415 | 4.262 | 3.672 | 4.034 | 3.608 | 3.894 | 0.154 | 0.004 | 2.164 | 2.164 | 4.682 |
| rno-miR-191a-3p | 1.485 | 1.093 | 0.511 | 1.353 | 1.434 | 1.175 | 0.179 | 2.999 | 2.692 | 2.627 | 1.547 | 2.466 | 0.317 | 0.007 | 2.098 | 2.098 | 4.404 |
| rno-miR-138-1-3p | 1.752 | 1.045 | 1.380 | 3.928 | 2.043 | 2.030 | 0.504 | 3.252 | 5.200 | 3.754 | 4.709 | 4.229 | 0.443 | 0.015 | 2.084 | 2.084 | 4.341 |
| rno-miR-1843-3p | 0.914 | 1.659 | 1.328 | 2.988 | 2.855 | 1.949 | 0.415 | 4.485 | 4.383 | 3.543 | 3.632 | 4.011 | 0.246 | 0.005 | 2.058 | 2.058 | 4.236 |
| rno-miR-140-5p | 1.676 | 2.120 | 1.412 | 6.337 | 7.245 | 3.758 | 1.252 | 7.824 | 7.623 | 7.442 | 7.131 | 7.505 | 0.147 | 0.034 | 1.997 | 1.997 | 3.989 |
| rno-miR-499-5p | 4.989 | 1.279 | 1.242 | 7.231 | 8.350 | 4.618 | 1.474 | 9.187 | 8.960 | 9.040 | 8.829 | 9.004 | 0.075 | 0.034 | 1.950 | 1.950 | 3.801 |
| rno-miR-29c-3p | 2.765 | 1.413 | 1.963 | 7.574 | 8.470 | 4.437 | 1.486 | 9.084 | 8.873 | 8.874 | 7.693 | 8.631 | 0.317 | 0.044 | 1.945 | 1.945 | 3.784 |
| rno-miR-539-5p | 1.629 | 1.272 | 1.404 | 1.395 | 1.304 | 1.401 | 0.062 | 2.835 | 3.774 | 2.068 | 2.172 | 2.712 | 0.393 | 0.007 | 1.936 | 1.936 | 3.748 |
| rno-miR-322-5p | 3.125 | 2.532 | 0.978 | 7.233 | 8.065 | 4.386 | 1.384 | 8.651 | 8.907 | 8.170 | 8.235 | 8.491 | 0.175 | 0.035 | 1.936 | 1.936 | 3.747 |
| rno-miR-363-3p | 1.111 | 1.710 | 0.676 | 1.003 | 1.107 | 1.121 | 0.167 | 2.777 | 2.731 | 1.903 | 1.249 | 2.165 | 0.365 | 0.027 | 1.930 | 1.930 | 3.727 |
| rno-miR-218a-5p | 1.006 | 0.796 | 0.838 | 0.853 | 1.063 | 0.911 | 0.052 | 1.523 | 1.671 | 1.974 | 1.860 | 1.757 | 0.100 | 0.000 | 1.928 | 1.928 | 3.716 |
| rno-miR-7578 | 1.023 | 0.998 | 0.964 | 2.853 | 3.948 | 1.957 | 0.614 | 4.077 | 3.645 | 3.362 | 3.826 | 3.728 | 0.151 | 0.041 | 1.905 | 1.905 | 3.628 |
| rno-miR-1839-3p | 1.029 | 0.881 | 1.490 | 1.505 | 1.752 | 1.331 | 0.162 | 2.547 | 3.442 | 2.261 | 1.892 | 2.536 | 0.331 | 0.010 | 1.905 | 1.905 | 3.627 |
| rno-miR-350 | 2.754 | 1.279 | 1.640 | 6.224 | 6.787 | 3.737 | 1.160 | 7.194 | 7.563 | 6.278 | 6.655 | 6.922 | 0.285 | 0.049 | 1.853 | 1.853 | 3.432 |
| rno-miR-369-5p | 0.905 | 1.019 | 1.032 | 0.667 | 0.713 | 0.867 | 0.076 | 1.973 | 2.206 | 0.842 | 1.394 | 1.604 | 0.306 | 0.035 | 1.850 | 1.850 | 3.421 |
| rno-miR-223-3p | 1.700 | 1.900 | 1.436 | 2.564 | 2.988 | 2.117 | 0.287 | 4.365 | 3.678 | 4.293 | 3.200 | 3.884 | 0.275 | 0.003 | 1.834 | 1.834 | 3.364 |
| rno-miR-335 | 0.918 | 1.499 | 1.281 | 1.235 | 1.183 | 1.223 | 0.093 | 2.839 | 2.258 | 1.854 | 1.912 | 2.216 | 0.226 | 0.003 | 1.812 | 1.812 | 3.282 |
| rno-miR-511-3p | 2.699 | 1.434 | 1.668 | 3.328 | 3.877 | 2.601 | 0.469 | 5.115 | 5.252 | 4.240 | 3.924 | 4.633 | 0.326 | 0.012 | 1.781 | 1.781 | 3.172 |
| rno-miR-98-5p | 4.829 | 2.088 | 1.445 | 3.645 | 4.981 | 3.398 | 0.712 | 6.048 | 5.289 | 6.842 | 5.975 | 6.038 | 0.318 | 0.018 | 1.777 | 1.777 | 3.159 |
| rno-mir-92b | 1.382 | 0.753 | 1.546 | 1.250 | 1.504 | 1.287 | 0.143 | 2.209 | 2.181 | 2.316 | 2.399 | 2.276 | 0.050 | 0.001 | 1.769 | 1.769 | 3.128 |
| rno-miR-495 | 0.715 | 1.079 | 1.103 | 0.854 | 1.243 | 0.999 | 0.095 | 1.957 | 1.372 | 1.435 | 2.248 | 1.753 | 0.211 | 0.010 | 1.756 | 1.756 | 3.082 |
| rno-miR-34b-3p | 1.774 | 1.738 | 2.213 | 4.458 | 4.551 | 2.947 | 0.642 | 5.080 | 5.605 | 4.554 | 5.013 | 5.063 | 0.215 | 0.026 | 1.718 | 1.718 | 2.953 |
| rno-miR-201-5p | 1.029 | 1.180 | 1.278 | 1.071 | 1.434 | 1.198 | 0.073 | 2.570 | 1.863 | 1.729 | 1.943 | 2.026 | 0.187 | 0.003 | 1.691 | 1.691 | 2.860 |
| rno-miR-7a-5p | 1.467 | 0.994 | 0.978 | 0.829 | 1.178 | 1.089 | 0.110 | 2.675 | 1.382 | 1.657 | 1.641 | 1.839 | 0.286 | 0.032 | 1.688 | 1.688 | 2.849 |
| rno-miR-872-3p | 2.566 | 1.592 | 2.460 | 4.511 | 5.480 | 3.322 | 0.720 | 5.965 | 6.364 | 5.847 | 4.061 | 5.559 | 0.511 | 0.047 | 1.674 | 1.674 | 2.801 |
| rno-miR-541-5p | 4.870 | 2.577 | 4.922 | 1.468 | 2.345 | 3.236 | 0.702 | 6.150 | 5.898 | 4.078 | 5.524 | 5.412 | 0.463 | 0.045 | 1.672 | 1.672 | 2.797 |
| rno-miR-3068-3p | 3.686 | 1.544 | 1.808 | 4.722 | 5.713 | 3.495 | 0.810 | 6.232 | 6.428 | 5.827 | 4.825 | 5.828 | 0.357 | 0.047 | 1.668 | 1.668 | 2.781 |
| rno-miR-92b-3p | 2.029 | 1.875 | 1.854 | 3.377 | 4.104 | 2.648 | 0.462 | 4.623 | 4.221 | 4.363 | 4.221 | 4.357 | 0.095 | 0.015 | 1.646 | 1.646 | 2.708 |
| rno-miR-26b-3p | 1.403 | 1.196 | 1.242 | 1.154 | 1.324 | 1.264 | 0.045 | 2.093 | 3.027 | 1.886 | 1.265 | 2.068 | 0.365 | 0.042 | 1.636 | 1.636 | 2.678 |
| rno-miR-429 | 1.864 | 1.488 | 1.348 | 1.228 | 1.857 | 1.557 | 0.131 | 3.021 | 2.330 | 1.889 | 2.766 | 2.501 | 0.249 | 0.009 | 1.607 | 1.607 | 2.581 |
| rno-miR-384-5p | 1.614 | 1.389 | 1.231 | 1.265 | 1.706 | 1.441 | 0.094 | 2.534 | 2.774 | 2.280 | 1.546 | 2.283 | 0.266 | 0.013 | 1.585 | 1.585 | 2.511 |
| rno-miR-200b-5p | 1.021 | 1.486 | 1.483 | 1.266 | 1.391 | 1.329 | 0.087 | 2.694 | 2.017 | 1.811 | 1.901 | 2.106 | 0.200 | 0.006 | 1.584 | 1.584 | 2.510 |
| rno-miR-598-3p | 0.988 | 1.175 | 1.351 | 1.246 | 1.639 | 1.280 | 0.107 | 2.751 | 2.178 | 1.257 | 1.922 | 2.027 | 0.310 | 0.041 | 1.584 | 1.584 | 2.509 |
| rno-miR-217-5p | 1.640 | 1.522 | 1.188 | 1.749 | 1.393 | 1.498 | 0.098 | 2.520 | 2.593 | 1.755 | 2.602 | 2.367 | 0.205 | 0.005 | 1.580 | 1.580 | 2.497 |
| rno-miR-200c-3p | 4.044 | 2.092 | 2.495 | 4.670 | 5.514 | 3.763 | 0.647 | 5.124 | 6.549 | 6.160 | 5.885 | 5.929 | 0.301 | 0.027 | 1.576 | 1.576 | 2.483 |
| rno-miR-338-5p | 4.345 | 1.247 | 4.014 | 4.911 | 4.826 | 3.868 | 0.675 | 6.131 | 6.367 | 5.221 | 6.388 | 6.027 | 0.275 | 0.031 | 1.558 | 1.558 | 2.427 |
| rno-miR-128-1-5p | 1.014 | 0.843 | 0.861 | 0.957 | 1.269 | 0.989 | 0.077 | 1.092 | 1.618 | 1.735 | 1.675 | 1.530 | 0.148 | 0.011 | 1.547 | 1.547 | 2.394 |
| rno-miR-379-5p | 4.438 | 2.486 | 3.780 | 4.276 | 3.483 | 3.693 | 0.347 | 6.470 | 6.202 | 4.382 | 5.778 | 5.708 | 0.464 | 0.009 | 1.546 | 1.546 | 2.390 |
| rno-miR-487b-3p | 4.903 | 2.661 | 4.527 | 3.449 | 3.036 | 3.715 | 0.431 | 6.528 | 6.435 | 4.002 | 5.877 | 5.710 | 0.587 | 0.026 | 1.537 | 1.537 | 2.363 |
| rno-miR-133c | 1.726 | 1.149 | 0.891 | 1.415 | 1.363 | 1.309 | 0.139 | 2.182 | 2.277 | 1.810 | 1.415 | 1.921 | 0.197 | 0.035 | 1.468 | 1.468 | 2.154 |
| rno-miR-219b | 1.400 | 1.140 | 1.744 | 1.810 | 1.422 | 1.503 | 0.123 | 1.987 | 2.135 | 2.420 | 2.268 | 2.202 | 0.092 | 0.003 | 1.465 | 1.465 | 2.146 |
| rno-mir-434 | 0.806 | 1.083 | 0.666 | 1.064 | 0.898 | 0.904 | 0.079 | 1.609 | 1.382 | 0.996 | 1.302 | 1.322 | 0.127 | 0.022 | 1.463 | 1.463 | 2.141 |
| rno-miR-483-5p | 4.671 | 3.464 | 2.154 | 3.557 | 2.722 | 3.314 | 0.425 | 4.412 | 4.533 | 4.905 | 5.216 | 4.766 | 0.183 | 0.024 | 1.438 | 1.438 | 2.069 |
| rno-miR-664-1-5p | 4.177 | 3.871 | 2.893 | 2.093 | 4.589 | 3.525 | 0.454 | 5.128 | 5.105 | 4.885 | 5.093 | 5.053 | 0.056 | 0.021 | 1.434 | 1.434 | 2.055 |
| rno-mir-224 | 1.439 | 1.082 | 1.380 | 0.979 | 0.948 | 1.166 | 0.102 | 1.902 | 1.715 | 1.204 | 1.856 | 1.669 | 0.160 | 0.028 | 1.432 | 1.432 | 2.051 |
| rno-miR-352 | 6.179 | 5.576 | 4.495 | 6.660 | 7.909 | 6.164 | 0.567 | 8.746 | 8.594 | 9.043 | 8.829 | 8.803 | 0.094 | 0.005 | 1.428 | 1.428 | 2.040 |
| rno-mir-487b | 0.947 | 1.011 | 1.111 | 0.560 | 0.937 | 0.914 | 0.094 | 1.541 | 1.406 | 0.960 | 1.287 | 1.298 | 0.124 | 0.039 | 1.421 | 1.421 | 2.020 |
| rno-miR-224-5p | 6.766 | 4.363 | 5.613 | 5.811 | 6.195 | 5.749 | 0.399 | 8.363 | 9.012 | 7.176 | 8.087 | 8.159 | 0.381 | 0.004 | 1.419 | 1.419 | 2.014 |
| rno-miR-500-5p | 1.030 | 1.098 | 1.290 | 1.003 | 1.114 | 1.107 | 0.050 | 1.700 | 1.932 | 1.488 | 1.163 | 1.571 | 0.163 | 0.020 | 1.419 | 1.419 | 2.013 |
| rno-miR-144-5p | 0.944 | 1.194 | 0.994 | 0.871 | 1.118 | 1.024 | 0.059 | 1.751 | 1.303 | 1.489 | 1.224 | 1.442 | 0.117 | 0.011 | 1.408 | 1.408 | 1.982 |
| rno-miR-494-3p | 3.121 | 2.629 | 3.830 | 4.884 | 4.948 | 3.882 | 0.463 | 5.693 | 5.579 | 5.208 | 5.226 | 5.427 | 0.123 | 0.024 | 1.398 | 1.398 | 1.954 |
| rno-mir-335 | 1.251 | 1.092 | 1.483 | 1.137 | 1.277 | 1.248 | 0.068 | 2.293 | 1.751 | 1.512 | 1.334 | 1.722 | 0.209 | 0.048 | 1.380 | 1.380 | 1.904 |
| rno-miR-3584-5p | 4.426 | 4.283 | 2.989 | 5.297 | 5.769 | 4.553 | 0.478 | 5.974 | 6.149 | 6.503 | 6.460 | 6.272 | 0.127 | 0.017 | 1.378 | 1.378 | 1.898 |
| rno-miR-9a-5p | 1.153 | 0.922 | 1.104 | 1.122 | 0.898 | 1.040 | 0.054 | 1.410 | 1.275 | 1.871 | 1.100 | 1.414 | 0.165 | 0.049 | 1.360 | 1.360 | 1.848 |
| rno-miR-381-5p | 0.855 | 1.024 | 0.973 | 0.905 | 1.086 | 0.969 | 0.041 | 1.476 | 1.416 | 1.174 | 1.176 | 1.310 | 0.079 | 0.005 | 1.353 | 1.353 | 1.830 |
| rno-miR-3589 | 0.808 | 0.862 | 0.627 | 0.685 | 0.905 | 0.778 | 0.053 | 0.910 | 0.963 | 1.002 | 1.289 | 1.041 | 0.085 | 0.028 | 1.339 | 1.339 | 1.792 |
| rno-miR-3586-3p | 1.019 | 0.882 | 0.866 | 0.784 | 0.921 | 0.894 | 0.038 | 1.127 | 1.106 | 1.141 | 1.416 | 1.197 | 0.073 | 0.006 | 1.339 | 1.339 | 1.792 |
| rno-mir-1306 | 1.297 | 1.017 | 0.825 | 0.929 | 1.160 | 1.046 | 0.083 | 1.600 | 1.167 | 1.597 | 1.230 | 1.398 | 0.116 | 0.039 | 1.337 | 1.337 | 1.788 |
| rno-miR-494-5p | 1.149 | 0.895 | 0.671 | 0.924 | 1.054 | 0.939 | 0.081 | 1.021 | 1.495 | 1.247 | 1.245 | 1.252 | 0.097 | 0.041 | 1.334 | 1.334 | 1.780 |
| rno-miR-10a-5p | 6.678 | 5.321 | 5.482 | 6.074 | 6.822 | 6.075 | 0.303 | 8.269 | 8.194 | 7.302 | 8.628 | 8.098 | 0.282 | 0.002 | 1.333 | 1.333 | 1.777 |
| rno-miR-224-3p | 3.757 | 3.614 | 3.131 | 3.204 | 3.518 | 3.445 | 0.120 | 5.113 | 5.627 | 4.095 | 3.480 | 4.579 | 0.485 | 0.039 | 1.329 | 1.329 | 1.767 |
| rno-miR-25-3p | 7.382 | 6.477 | 5.227 | 8.088 | 8.842 | 7.203 | 0.630 | 9.754 | 9.902 | 8.931 | 9.559 | 9.536 | 0.214 | 0.016 | 1.324 | 1.324 | 1.753 |
| rno-let-7c-1-3p | 0.808 | 0.903 | 0.835 | 0.894 | 1.071 | 0.902 | 0.046 | 0.988 | 1.319 | 1.447 | 1.005 | 1.190 | 0.115 | 0.039 | 1.319 | 1.319 | 1.739 |
| rno-miR-742-5p | 0.856 | 0.831 | 0.760 | 0.849 | 1.118 | 0.883 | 0.061 | 1.260 | 1.155 | 1.066 | 1.147 | 1.157 | 0.040 | 0.010 | 1.311 | 1.311 | 1.718 |
| rno-miR-3561-3p | 0.923 | 0.686 | 1.042 | 0.846 | 0.605 | 0.820 | 0.079 | 1.033 | 1.073 | 1.129 | 1.047 | 1.070 | 0.021 | 0.029 | 1.305 | 1.305 | 1.703 |
| rno-let-7f-5p | 9.418 | 7.339 | 6.681 | 10.266 | 11.152 | 8.971 | 0.853 | 11.647 | 11.564 | 11.883 | 11.658 | 11.688 | 0.068 | 0.026 | 1.303 | 1.303 | 1.697 |
| rno-miR-146b-5p | 5.596 | 4.544 | 6.691 | 7.842 | 8.001 | 6.535 | 0.661 | 8.497 | 8.624 | 8.007 | 8.597 | 8.431 | 0.144 | 0.041 | 1.290 | 1.290 | 1.665 |
| rno-mir-665 | 2.224 | 1.775 | 1.718 | 2.780 | 2.417 | 2.183 | 0.199 | 2.962 | 2.904 | 2.975 | 2.415 | 2.814 | 0.134 | 0.042 | 1.289 | 1.289 | 1.662 |
| rno-mir-145 | 0.966 | 1.279 | 1.242 | 1.517 | 1.315 | 1.264 | 0.088 | 1.546 | 1.584 | 1.740 | 1.579 | 1.612 | 0.043 | 0.014 | 1.276 | 1.276 | 1.627 |
| rno-miR-100-3p | 0.625 | 0.597 | 0.623 | 0.776 | 0.709 | 0.666 | 0.033 | 0.767 | 0.823 | 0.815 | 0.976 | 0.845 | 0.045 | 0.014 | 1.269 | 1.269 | 1.611 |
| rno-miR-323-3p | 0.751 | 1.137 | 0.973 | 0.878 | 1.072 | 0.962 | 0.069 | 1.167 | 1.145 | 1.143 | 1.289 | 1.186 | 0.035 | 0.032 | 1.233 | 1.233 | 1.520 |
| rno-mir-494 | 1.340 | 1.788 | 2.100 | 1.761 | 1.976 | 1.793 | 0.129 | 2.323 | 2.402 | 1.928 | 2.184 | 2.209 | 0.104 | 0.047 | 1.232 | 1.232 | 1.519 |
| rno-miR-449a-3p | 0.860 | 0.830 | 1.121 | 0.934 | 0.835 | 0.916 | 0.055 | 1.171 | 1.223 | 1.126 | 0.960 | 1.120 | 0.057 | 0.037 | 1.223 | 1.223 | 1.495 |
| rno-miR-3561-5p | 0.812 | 1.045 | 1.103 | 0.886 | 1.111 | 0.991 | 0.060 | 1.214 | 1.344 | 1.161 | 1.112 | 1.208 | 0.050 | 0.032 | 1.219 | 1.219 | 1.485 |
| rno-mir-344g | 1.024 | 1.302 | 1.333 | 1.304 | 1.354 | 1.263 | 0.061 | 1.344 | 1.542 | 1.640 | 1.625 | 1.538 | 0.068 | 0.020 | 1.217 | 1.217 | 1.481 |
| rno-miR-27b-5p | 4.757 | 4.801 | 3.854 | 4.928 | 5.969 | 4.862 | 0.336 | 6.001 | 6.266 | 5.345 | 6.005 | 5.904 | 0.197 | 0.042 | 1.214 | 1.214 | 1.475 |
| rno-mir-298 | 1.120 | 1.559 | 1.681 | 1.391 | 1.491 | 1.448 | 0.095 | 1.670 | 1.762 | 1.785 | 1.802 | 1.755 | 0.029 | 0.028 | 1.211 | 1.211 | 1.468 |
| rno-mir-496 | 0.945 | 0.844 | 1.061 | 1.003 | 1.112 | 0.993 | 0.047 | 1.321 | 1.202 | 1.110 | 1.156 | 1.197 | 0.045 | 0.018 | 1.205 | 1.205 | 1.453 |
| rno-mir-29b-2 | 1.345 | 1.298 | 1.456 | 1.298 | 1.198 | 1.319 | 0.042 | 1.410 | 1.726 | 1.378 | 1.822 | 1.584 | 0.112 | 0.045 | 1.201 | 1.201 | 1.442 |
| rno-miR-20b-5p | 6.444 | 5.878 | 5.959 | 7.396 | 7.789 | 6.693 | 0.385 | 8.221 | 8.346 | 7.678 | 7.882 | 8.032 | 0.153 | 0.022 | 1.200 | 1.200 | 1.440 |
| rno-miR-146a-5p | 8.247 | 7.606 | 8.694 | 10.253 | 11.155 | 9.191 | 0.657 | 11.193 | 11.032 | 10.715 | 11.095 | 11.009 | 0.103 | 0.046 | 1.198 | 1.198 | 1.435 |
| rno-mir-376b | 1.080 | 1.333 | 1.156 | 1.214 | 1.070 | 1.171 | 0.048 | 1.433 | 1.452 | 1.342 | 1.381 | 1.402 | 0.025 | 0.006 | 1.198 | 1.198 | 1.434 |
| rno-miR-1193-3p | 1.032 | 1.013 | 0.973 | 1.147 | 1.196 | 1.072 | 0.042 | 1.122 | 1.207 | 1.379 | 1.413 | 1.280 | 0.069 | 0.031 | 1.194 | 1.194 | 1.427 |
| rno-miR-199a-3p | 10.078 | 9.137 | 8.564 | 10.378 | 10.937 | 9.819 | 0.428 | 11.770 | 12.054 | 11.174 | 11.540 | 11.635 | 0.186 | 0.009 | 1.185 | 1.185 | 1.404 |
| rno-mir-495 | 1.243 | 1.197 | 0.948 | 1.219 | 1.098 | 1.141 | 0.054 | 1.398 | 1.478 | 1.202 | 1.262 | 1.335 | 0.063 | 0.051 | 1.170 | 1.170 | 1.369 |
| rno-mir-155 | 1.032 | 1.105 | 1.218 | 1.321 | 1.401 | 1.216 | 0.068 | 1.344 | 1.522 | 1.337 | 1.483 | 1.422 | 0.047 | 0.050 | 1.169 | 1.169 | 1.367 |
| rno-mir-451 | 1.193 | 1.155 | 1.242 | 1.098 | 1.190 | 1.176 | 0.024 | 1.240 | 1.264 | 1.603 | 1.361 | 1.367 | 0.083 | 0.043 | 1.163 | 1.163 | 1.352 |
| rno-miR-29a-3p | 11.322 | 10.109 | 10.103 | 12.294 | 12.640 | 11.293 | 0.531 | 13.158 | 13.166 | 13.027 | 12.454 | 12.951 | 0.169 | 0.032 | 1.147 | 1.147 | 1.315 |
| rno-miR-27b-3p | 10.915 | 10.166 | 10.089 | 12.259 | 12.475 | 11.181 | 0.507 | 12.942 | 12.921 | 12.772 | 12.638 | 12.818 | 0.071 | 0.025 | 1.146 | 1.146 | 1.314 |
| rno-miR-1-3p | 11.523 | 10.778 | 11.276 | 12.309 | 12.925 | 11.762 | 0.382 | 13.305 | 13.262 | 13.632 | 13.456 | 13.414 | 0.084 | 0.007 | 1.140 | 1.140 | 1.301 |
| rno-mir-26a | 2.703 | 2.209 | 2.676 | 2.457 | 2.856 | 2.580 | 0.113 | 2.887 | 2.742 | 2.973 | 3.079 | 2.920 | 0.071 | 0.048 | 1.132 | 1.132 | 1.281 |
| rno-let-7c-2 | 1.152 | 1.259 | 1.045 | 1.328 | 1.194 | 1.196 | 0.048 | 1.349 | 1.349 | 1.380 | 1.309 | 1.347 | 0.014 | 0.030 | 1.126 | 1.126 | 1.269 |
| rno-miR-15b-5p | 9.555 | 8.723 | 9.040 | 8.977 | 9.185 | 9.096 | 0.137 | 10.333 | 10.434 | 9.427 | 10.140 | 10.084 | 0.227 | 0.006 | 1.109 | 1.109 | 1.229 |
| rno-let-7i-5p | 12.580 | 11.339 | 10.957 | 11.859 | 12.355 | 11.818 | 0.303 | 12.811 | 12.859 | 12.769 | 12.923 | 12.841 | 0.033 | 0.021 | 1.087 | 1.087 | 1.181 |
| rno-miR-26a-5p | 13.567 | 13.204 | 12.866 | 14.632 | 14.854 | 13.825 | 0.393 | 14.901 | 15.098 | 14.886 | 14.922 | 14.952 | 0.049 | 0.040 | 1.082 | 1.082 | 1.170 |
| rno-miR-92a-3p | 10.319 | 10.171 | 9.719 | 10.565 | 11.241 | 10.403 | 0.251 | 11.329 | 11.267 | 10.793 | 11.425 | 11.203 | 0.141 | 0.036 | 1.077 | 1.077 | 1.160 |
| rno-miR-195-5p | 11.880 | 11.448 | 10.765 | 12.192 | 12.113 | 11.679 | 0.263 | 12.609 | 12.703 | 12.311 | 12.674 | 12.574 | 0.090 | 0.023 | 1.077 | 1.077 | 1.159 |
| rno-miR-126a-3p | 12.450 | 12.348 | 12.219 | 13.624 | 13.684 | 12.865 | 0.324 | 13.963 | 13.907 | 13.904 | 13.594 | 13.842 | 0.084 | 0.035 | 1.076 | 1.076 | 1.158 |
| rno-miR-221-3p | 9.598 | 8.920 | 8.811 | 9.262 | 9.444 | 9.207 | 0.150 | 9.987 | 9.941 | 9.472 | 10.179 | 9.895 | 0.150 | 0.015 | 1.075 | 1.075 | 1.155 |
| spike_in-control-29 | 13.803 | 13.823 | 13.965 | 15.034 | 15.171 | 14.359 | 0.306 | 15.296 | 15.319 | 15.132 | 15.057 | 15.201 | 0.063 | 0.047 | 1.059 | 1.059 | 1.121 |
| rno-let-7a-5p | 13.802 | 13.596 | 13.649 | 13.540 | 13.900 | 13.698 | 0.067 | 14.076 | 14.021 | 14.416 | 14.256 | 14.192 | 0.090 | 0.003 | 1.036 | 1.036 | 1.074 |
| rno-miR-125b-5p | 13.785 | 13.593 | 13.824 | 13.599 | 13.938 | 13.748 | 0.067 | 14.181 | 14.214 | 13.973 | 14.012 | 14.095 | 0.060 | 0.007 | 1.025 | 1.025 | 1.051 |
| rno-miR-125a-5p | 12.838 | 12.818 | 12.815 | 12.900 | 13.108 | 12.896 | 0.055 | 13.224 | 13.310 | 12.899 | 13.346 | 13.195 | 0.102 | 0.029 | 1.023 | 1.023 | 1.047 |
| rno-miR-23a-3p | 14.562 | 14.394 | 14.442 | 14.455 | 14.624 | 14.496 | 0.042 | 14.819 | 14.887 | 14.766 | 14.754 | 14.807 | 0.030 | 0.001 | 1.021 | 1.021 | 1.043 |
| rno-let-7c-5p | 14.868 | 14.747 | 14.802 | 14.818 | 14.947 | 14.837 | 0.034 | 15.081 | 15.030 | 15.240 | 15.154 | 15.126 | 0.046 | 0.001 | 1.020 | 1.020 | 1.039 |
| rno-miR-23b-3p | 14.568 | 14.537 | 14.475 | 14.520 | 14.531 | 14.526 | 0.015 | 14.697 | 14.696 | 14.581 | 14.700 | 14.669 | 0.029 | 0.002 | 1.010 | 1.010 | 1.020 |
| AFFX-r2-P1-c2-cre-5 | 15.160 | 15.302 | 15.296 | 15.273 | 15.338 | 15.274 | 0.030 | 15.319 | 15.359 | 15.450 | 15.374 | 15.376 | 0.028 | 0.045 | 1.007 | 1.007 | 1.013 |
| AFFX-r2-P1-c2-cre-3 | 15.229 | 15.270 | 15.230 | 15.329 | 15.337 | 15.279 | 0.023 | 15.356 | 15.398 | 15.324 | 15.356 | 15.359 | 0.015 | 0.031 | 1.005 | 1.005 | 1.010 |
| AFFX-r2-P1-c1-cre-3 | 15.412 | 15.321 | 15.368 | 15.357 | 15.339 | 15.359 | 0.016 | 15.401 | 15.426 | 15.413 | 15.395 | 15.409 | 0.007 | 0.033 | 1.003 | 1.003 | 1.006 |
| rno-miR-24-3p | 14.994 | 14.860 | 14.713 | 14.818 | 14.780 | 14.833 | 0.047 | 14.622 | 14.610 | 14.617 | 14.580 | 14.607 | 0.009 | 0.004 | 0.985 | -1.015 | -1.031 |
| rno-miR-191a-5p | 14.138 | 14.250 | 14.325 | 13.777 | 13.578 | 14.014 | 0.144 | 13.511 | 13.574 | 13.386 | 13.828 | 13.575 | 0.093 | 0.047 | 0.969 | -1.032 | -1.066 |
| rno-miR-181a-5p | 12.417 | 12.425 | 12.091 | 12.264 | 12.220 | 12.283 | 0.063 | 11.906 | 11.981 | 11.719 | 11.931 | 11.884 | 0.057 | 0.003 | 0.968 | -1.034 | -1.068 |
| rno-miR-378b | 13.355 | 13.377 | 13.677 | 12.858 | 12.874 | 13.228 | 0.158 | 12.472 | 12.555 | 12.600 | 12.922 | 12.637 | 0.098 | 0.021 | 0.955 | -1.047 | -1.096 |
| rno-miR-103-3p | 13.965 | 14.050 | 13.941 | 13.436 | 13.169 | 13.712 | 0.173 | 12.982 | 13.007 | 12.902 | 13.349 | 13.060 | 0.099 | 0.019 | 0.952 | -1.050 | -1.102 |
| rno-miR-24-2-5p | 10.996 | 10.958 | 10.614 | 10.302 | 10.413 | 10.657 | 0.140 | 10.195 | 10.258 | 9.843 | 10.225 | 10.130 | 0.097 | 0.022 | 0.951 | -1.052 | -1.107 |
| rno-miR-378a-3p | 14.198 | 14.297 | 14.467 | 13.700 | 13.539 | 14.040 | 0.179 | 13.147 | 13.097 | 13.233 | 13.588 | 13.266 | 0.111 | 0.011 | 0.945 | -1.058 | -1.120 |
| rno-miR-151-5p | 12.902 | 12.967 | 12.888 | 12.055 | 11.883 | 12.539 | 0.235 | 11.854 | 11.796 | 11.511 | 12.134 | 11.824 | 0.128 | 0.042 | 0.943 | -1.060 | -1.125 |
| rno-miR-17-5p | 11.551 | 11.829 | 11.609 | 11.081 | 10.904 | 11.395 | 0.173 | 10.820 | 10.913 | 10.351 | 10.793 | 10.719 | 0.125 | 0.020 | 0.941 | -1.063 | -1.130 |
| AFFX-r2-Ec-c3-bioC-5 | 13.395 | 13.787 | 13.827 | 13.072 | 12.644 | 13.345 | 0.223 | 12.401 | 12.369 | 12.184 | 12.999 | 12.489 | 0.177 | 0.023 | 0.936 | -1.069 | -1.142 |
| AFFX-r2-Ec-c2-bioC-5 | 13.332 | 13.747 | 13.862 | 13.014 | 12.593 | 13.309 | 0.234 | 12.378 | 12.351 | 12.101 | 12.983 | 12.453 | 0.187 | 0.029 | 0.936 | -1.069 | -1.142 |
| AFFX-r2-Ec-c3-bioC-3 | 13.226 | 13.711 | 13.671 | 12.767 | 12.456 | 13.166 | 0.247 | 12.224 | 12.268 | 11.856 | 12.868 | 12.304 | 0.210 | 0.037 | 0.935 | -1.070 | -1.145 |
| AFFX-r2-Ec-c2-bioC-3 | 13.191 | 13.665 | 13.642 | 12.681 | 12.364 | 13.109 | 0.259 | 12.147 | 12.207 | 11.829 | 12.814 | 12.249 | 0.206 | 0.041 | 0.934 | -1.070 | -1.145 |
| AFFX-r2-Ec-c1-bioC-5 | 13.267 | 13.818 | 13.928 | 13.065 | 12.597 | 13.335 | 0.246 | 12.385 | 12.327 | 12.107 | 12.981 | 12.450 | 0.187 | 0.029 | 0.934 | -1.071 | -1.147 |
| AFFX-r2-Ec-c1-bioC-3 | 13.264 | 13.689 | 13.786 | 12.755 | 12.448 | 13.188 | 0.260 | 12.223 | 12.247 | 11.950 | 12.797 | 12.304 | 0.178 | 0.033 | 0.933 | -1.072 | -1.149 |
| rno-miR-185-5p | 13.362 | 13.503 | 13.237 | 12.406 | 12.318 | 12.965 | 0.250 | 12.053 | 11.995 | 11.824 | 12.455 | 12.082 | 0.133 | 0.024 | 0.932 | -1.073 | -1.152 |
| rno-miR-99b-5p | 13.106 | 13.185 | 13.041 | 12.410 | 11.877 | 12.724 | 0.253 | 11.854 | 11.962 | 11.401 | 12.149 | 11.841 | 0.159 | 0.028 | 0.931 | -1.075 | -1.155 |
| rno-miR-151-3p | 10.603 | 10.688 | 10.731 | 10.003 | 9.829 | 10.371 | 0.189 | 9.689 | 9.618 | 9.190 | 10.036 | 9.633 | 0.174 | 0.026 | 0.929 | -1.077 | -1.159 |
| AFFX-r2-Ec-c3-bioB-3 | 10.722 | 11.217 | 10.869 | 10.229 | 10.008 | 10.609 | 0.219 | 9.803 | 9.760 | 9.333 | 10.360 | 9.814 | 0.211 | 0.037 | 0.925 | -1.081 | -1.169 |
| rno-miR-1224 | 7.264 | 7.707 | 7.856 | 6.989 | 7.070 | 7.377 | 0.173 | 6.778 | 6.864 | 6.491 | 7.132 | 6.816 | 0.132 | 0.043 | 0.924 | -1.082 | -1.171 |
| AFFX-r2-Ec-c2-bioB-3 | 10.799 | 11.300 | 11.289 | 10.300 | 9.976 | 10.733 | 0.264 | 9.895 | 9.874 | 9.443 | 10.394 | 9.901 | 0.194 | 0.047 | 0.923 | -1.084 | -1.175 |
| rno-miR-532-5p | 9.268 | 8.911 | 8.754 | 8.350 | 8.204 | 8.697 | 0.192 | 8.047 | 8.306 | 7.622 | 7.995 | 7.993 | 0.141 | 0.026 | 0.919 | -1.088 | -1.184 |
| AFFX-r2-Ec-c3-bioB-M | 11.957 | 12.559 | 12.490 | 11.466 | 11.161 | 11.927 | 0.276 | 10.958 | 10.870 | 10.468 | 11.527 | 10.956 | 0.218 | 0.033 | 0.919 | -1.089 | -1.185 |
| AFFX-r2-Ec-c2-bioB-M | 11.962 | 12.470 | 12.492 | 11.448 | 11.044 | 11.883 | 0.284 | 10.897 | 10.853 | 10.435 | 11.478 | 10.915 | 0.214 | 0.036 | 0.919 | -1.089 | -1.185 |
| AFFX-r2-Ec-c1-bioB-M | 11.843 | 12.628 | 12.704 | 11.554 | 11.146 | 11.975 | 0.303 | 10.950 | 10.937 | 10.608 | 11.428 | 10.981 | 0.169 | 0.033 | 0.917 | -1.091 | -1.189 |
| AFFX-r2-Ec-c2-bioB-5 | 11.207 | 11.798 | 11.783 | 10.728 | 10.254 | 11.154 | 0.300 | 10.191 | 10.121 | 9.662 | 10.711 | 10.171 | 0.215 | 0.040 | 0.912 | -1.097 | -1.203 |
| AFFX-r2-Ec-c3-bioB-5 | 11.099 | 11.654 | 11.582 | 10.632 | 10.209 | 11.035 | 0.277 | 10.026 | 10.050 | 9.544 | 10.582 | 10.051 | 0.212 | 0.031 | 0.911 | -1.098 | -1.206 |
| rno-miR-140-3p | 12.118 | 12.455 | 12.374 | 11.556 | 11.160 | 11.933 | 0.249 | 10.854 | 10.801 | 10.590 | 11.210 | 10.864 | 0.129 | 0.010 | 0.910 | -1.098 | -1.206 |
| AFFX-r2-Ec-c1-bioB-5 | 11.016 | 11.792 | 11.913 | 10.730 | 10.257 | 11.142 | 0.315 | 10.134 | 10.068 | 9.691 | 10.665 | 10.139 | 0.200 | 0.040 | 0.910 | -1.099 | -1.207 |
| rno-miR-532-3p | 9.155 | 9.294 | 9.449 | 8.256 | 8.244 | 8.879 | 0.261 | 8.222 | 8.439 | 7.458 | 8.101 | 8.055 | 0.211 | 0.050 | 0.907 | -1.102 | -1.215 |
| rno-miR-93-5p | 11.365 | 11.540 | 11.180 | 10.539 | 10.285 | 10.982 | 0.243 | 9.995 | 10.125 | 9.642 | 9.922 | 9.921 | 0.102 | 0.008 | 0.903 | -1.107 | -1.225 |
| rno-mir-351 | 6.992 | 6.121 | 6.612 | 5.959 | 5.871 | 6.311 | 0.213 | 5.877 | 5.551 | 5.554 | 5.808 | 5.698 | 0.085 | 0.046 | 0.903 | -1.108 | -1.227 |
| rno-miR-378a-5p | 10.903 | 11.031 | 11.260 | 10.282 | 10.450 | 10.785 | 0.182 | 9.851 | 9.753 | 9.608 | 9.505 | 9.679 | 0.077 | 0.001 | 0.897 | -1.114 | -1.242 |
| rno-miR-30c-2-3p | 10.126 | 10.476 | 10.265 | 9.144 | 9.001 | 9.802 | 0.304 | 8.895 | 8.797 | 8.249 | 9.211 | 8.788 | 0.200 | 0.034 | 0.897 | -1.115 | -1.244 |
| rno-miR-351-5p | 10.937 | 11.077 | 11.082 | 10.052 | 9.806 | 10.591 | 0.274 | 9.617 | 9.597 | 8.967 | 9.715 | 9.474 | 0.171 | 0.014 | 0.895 | -1.118 | -1.250 |
| rno-miR-455-3p | 10.152 | 10.622 | 10.719 | 9.498 | 8.937 | 9.985 | 0.340 | 8.896 | 9.041 | 8.284 | 9.500 | 8.930 | 0.251 | 0.049 | 0.894 | -1.118 | -1.250 |
| rno-miR-652-3p | 11.852 | 12.309 | 11.937 | 10.920 | 10.696 | 11.543 | 0.312 | 10.337 | 10.371 | 9.829 | 10.739 | 10.319 | 0.187 | 0.016 | 0.894 | -1.119 | -1.251 |
| rno-miR-324-5p | 9.417 | 9.446 | 9.177 | 8.520 | 8.192 | 8.950 | 0.253 | 8.083 | 8.327 | 7.543 | 8.038 | 7.998 | 0.164 | 0.021 | 0.894 | -1.119 | -1.252 |
| rno-miR-125b-2-3p | 10.018 | 9.909 | 9.600 | 9.053 | 8.837 | 9.484 | 0.233 | 8.592 | 8.665 | 7.908 | 8.631 | 8.449 | 0.181 | 0.012 | 0.891 | -1.122 | -1.260 |
| rno-miR-320-3p | 13.019 | 13.274 | 13.299 | 12.163 | 11.724 | 12.696 | 0.319 | 11.176 | 11.127 | 10.975 | 11.856 | 11.283 | 0.196 | 0.010 | 0.889 | -1.125 | -1.266 |
| rno-mir-378a | 3.467 | 3.142 | 3.136 | 2.830 | 2.882 | 3.091 | 0.113 | 2.620 | 2.712 | 2.725 | 2.890 | 2.737 | 0.056 | 0.037 | 0.885 | -1.130 | -1.276 |
| rno-miR-345-3p | 9.004 | 8.945 | 8.449 | 7.590 | 7.780 | 8.354 | 0.291 | 7.405 | 7.677 | 6.741 | 7.725 | 7.387 | 0.227 | 0.041 | 0.884 | -1.131 | -1.279 |
| rno-miR-345-5p | 9.219 | 9.044 | 8.505 | 8.225 | 8.604 | 8.719 | 0.181 | 7.922 | 8.189 | 7.374 | 7.022 | 7.626 | 0.263 | 0.010 | 0.875 | -1.143 | -1.307 |
| rno-miR-125a-3p | 7.985 | 7.811 | 7.687 | 7.003 | 6.881 | 7.473 | 0.223 | 6.325 | 6.730 | 6.348 | 6.743 | 6.536 | 0.116 | 0.011 | 0.875 | -1.143 | -1.307 |
| rno-miR-542-5p | 7.876 | 7.565 | 7.548 | 6.809 | 6.666 | 7.293 | 0.235 | 6.309 | 6.629 | 6.052 | 6.497 | 6.372 | 0.125 | 0.015 | 0.874 | -1.145 | -1.310 |
| rno-miR-423-3p | 10.050 | 10.210 | 10.029 | 8.927 | 8.751 | 9.594 | 0.311 | 8.531 | 8.413 | 7.910 | 8.643 | 8.374 | 0.162 | 0.015 | 0.873 | -1.146 | -1.312 |
| rno-miR-106b-3p | 8.936 | 8.807 | 8.980 | 7.705 | 7.446 | 8.375 | 0.330 | 7.503 | 7.396 | 6.809 | 7.470 | 7.295 | 0.163 | 0.031 | 0.871 | -1.148 | -1.318 |
| rno-miR-674-5p | 11.048 | 11.562 | 11.601 | 9.938 | 9.674 | 10.764 | 0.406 | 9.344 | 9.244 | 8.921 | 9.884 | 9.348 | 0.200 | 0.024 | 0.868 | -1.151 | -1.326 |
| rno-miR-503-5p | 9.711 | 9.715 | 9.450 | 8.805 | 8.473 | 9.231 | 0.252 | 8.035 | 8.073 | 7.847 | 8.027 | 7.995 | 0.050 | 0.004 | 0.866 | -1.155 | -1.333 |
| rno-mir-423 | 7.294 | 7.660 | 7.082 | 6.471 | 6.317 | 6.965 | 0.252 | 6.140 | 6.230 | 5.691 | 6.063 | 6.031 | 0.118 | 0.018 | 0.866 | -1.155 | -1.334 |
| rno-miR-500-3p | 9.605 | 9.899 | 9.808 | 8.735 | 8.323 | 9.274 | 0.315 | 8.026 | 8.099 | 7.600 | 8.288 | 8.003 | 0.145 | 0.012 | 0.863 | -1.159 | -1.343 |
| rno-miR-664-2-5p | 7.580 | 7.678 | 7.738 | 6.166 | 6.381 | 7.109 | 0.344 | 6.121 | 6.125 | 5.831 | 6.329 | 6.102 | 0.103 | 0.040 | 0.858 | -1.165 | -1.357 |
| rno-miR-490-3p | 8.861 | 8.715 | 7.736 | 7.521 | 7.191 | 8.005 | 0.332 | 7.031 | 7.162 | 6.415 | 6.831 | 6.859 | 0.163 | 0.025 | 0.857 | -1.167 | -1.362 |
| rno-miR-30c-1-3p | 8.863 | 9.060 | 8.631 | 7.498 | 7.357 | 8.282 | 0.356 | 7.082 | 7.120 | 6.746 | 7.431 | 7.095 | 0.140 | 0.026 | 0.857 | -1.167 | -1.363 |
| rno-miR-17-1-3p | 8.006 | 8.002 | 7.470 | 6.799 | 6.487 | 7.353 | 0.310 | 6.336 | 6.598 | 5.922 | 6.261 | 6.279 | 0.139 | 0.023 | 0.854 | -1.171 | -1.371 |
| rno-miR-339-5p | 9.448 | 9.445 | 9.252 | 8.743 | 8.523 | 9.082 | 0.190 | 7.896 | 8.001 | 7.545 | 7.469 | 7.728 | 0.130 | 0.001 | 0.851 | -1.175 | -1.381 |
| rno-miR-339-3p | 9.577 | 9.732 | 9.624 | 8.482 | 8.209 | 9.125 | 0.322 | 7.788 | 7.946 | 7.176 | 8.112 | 7.755 | 0.204 | 0.012 | 0.850 | -1.177 | -1.384 |
| rno-miR-214-3p | 11.723 | 12.161 | 12.185 | 10.722 | 10.296 | 11.417 | 0.386 | 9.437 | 9.752 | 9.318 | 10.238 | 9.686 | 0.206 | 0.008 | 0.848 | -1.179 | -1.389 |
| rno-miR-346 | 8.280 | 8.235 | 7.837 | 8.017 | 7.147 | 7.903 | 0.205 | 6.805 | 6.939 | 6.221 | 6.782 | 6.687 | 0.159 | 0.003 | 0.846 | -1.182 | -1.397 |
| rno-miR-210-3p | 11.358 | 11.764 | 10.880 | 10.550 | 9.984 | 10.907 | 0.310 | 9.266 | 9.427 | 8.932 | 9.264 | 9.222 | 0.104 | 0.002 | 0.846 | -1.183 | -1.399 |
| rno-miR-490-5p | 8.035 | 7.711 | 7.448 | 6.655 | 6.277 | 7.225 | 0.329 | 6.412 | 6.802 | 5.481 | 5.651 | 6.087 | 0.313 | 0.044 | 0.842 | -1.187 | -1.409 |
| rno-miR-210-5p | 6.881 | 6.706 | 6.535 | 5.735 | 5.678 | 6.307 | 0.251 | 5.195 | 5.630 | 4.883 | 5.357 | 5.266 | 0.156 | 0.013 | 0.835 | -1.198 | -1.434 |
| rno-miR-759 | 1.253 | 0.994 | 1.100 | 0.970 | 1.078 | 1.079 | 0.050 | 0.984 | 0.892 | 0.789 | 0.936 | 0.900 | 0.042 | 0.033 | 0.834 | -1.199 | -1.437 |
| rno-miR-181c-3p | 7.032 | 6.950 | 5.988 | 6.212 | 5.822 | 6.401 | 0.249 | 5.608 | 5.827 | 5.137 | 4.778 | 5.337 | 0.236 | 0.019 | 0.834 | -1.199 | -1.438 |
| rno-miR-143-5p | 7.534 | 7.108 | 5.810 | 6.451 | 6.394 | 6.660 | 0.300 | 5.911 | 5.937 | 5.482 | 4.877 | 5.552 | 0.248 | 0.029 | 0.834 | -1.200 | -1.439 |
| rno-miR-421-3p | 1.143 | 1.438 | 1.314 | 1.071 | 1.120 | 1.217 | 0.069 | 0.920 | 1.084 | 1.072 | 0.981 | 1.014 | 0.039 | 0.049 | 0.833 | -1.200 | -1.440 |
| rno-miR-99b-3p | 7.269 | 7.476 | 7.091 | 6.413 | 5.956 | 6.841 | 0.284 | 5.863 | 6.146 | 5.332 | 5.458 | 5.700 | 0.187 | 0.016 | 0.833 | -1.200 | -1.440 |
| rno-miR-3579 | 1.105 | 0.851 | 0.973 | 0.872 | 1.113 | 0.983 | 0.055 | 0.744 | 0.878 | 0.863 | 0.790 | 0.819 | 0.031 | 0.048 | 0.833 | -1.201 | -1.441 |
| rno-miR-25-5p | 8.857 | 8.814 | 9.322 | 7.190 | 6.847 | 8.206 | 0.496 | 6.756 | 6.809 | 6.448 | 7.288 | 6.825 | 0.173 | 0.050 | 0.832 | -1.202 | -1.446 |
| rno-miR-874-3p | 6.855 | 6.675 | 6.545 | 6.007 | 5.406 | 6.298 | 0.264 | 5.414 | 5.564 | 4.642 | 5.327 | 5.236 | 0.204 | 0.019 | 0.832 | -1.203 | -1.446 |
| rno-miR-324-3p | 8.638 | 9.147 | 8.937 | 7.593 | 7.558 | 8.375 | 0.336 | 7.150 | 7.110 | 6.427 | 7.063 | 6.938 | 0.171 | 0.010 | 0.828 | -1.207 | -1.457 |
| rno-miR-139-3p | 7.984 | 8.102 | 7.371 | 6.749 | 6.623 | 7.366 | 0.305 | 6.142 | 6.290 | 5.634 | 6.171 | 6.059 | 0.145 | 0.009 | 0.823 | -1.216 | -1.478 |
| rno-mir-6332 | 1.640 | 2.042 | 1.547 | 1.658 | 2.044 | 1.786 | 0.107 | 1.330 | 1.434 | 1.526 | 1.556 | 1.462 | 0.051 | 0.040 | 0.818 | -1.222 | -1.493 |
| rno-miR-423-5p | 9.818 | 10.209 | 10.224 | 8.681 | 8.429 | 9.472 | 0.384 | 7.595 | 7.593 | 7.450 | 8.253 | 7.723 | 0.180 | 0.007 | 0.815 | -1.227 | -1.504 |
| rno-mir-292 | 1.312 | 1.140 | 1.230 | 1.327 | 1.148 | 1.232 | 0.039 | 1.029 | 1.034 | 0.943 | 1.006 | 1.003 | 0.021 | 0.002 | 0.814 | -1.228 | -1.508 |
| rno-miR-652-5p | 7.086 | 6.433 | 6.986 | 5.919 | 5.852 | 6.455 | 0.258 | 4.879 | 5.486 | 5.139 | 5.515 | 5.255 | 0.152 | 0.007 | 0.814 | -1.228 | -1.509 |
| rno-miR-880-5p | 0.926 | 0.792 | 1.116 | 0.939 | 1.085 | 0.972 | 0.059 | 0.848 | 0.839 | 0.682 | 0.782 | 0.788 | 0.038 | 0.043 | 0.811 | -1.233 | -1.521 |
| rno-miR-146b-3p | 1.077 | 1.327 | 1.106 | 1.178 | 1.096 | 1.157 | 0.046 | 1.007 | 0.992 | 0.888 | 0.863 | 0.937 | 0.036 | 0.009 | 0.810 | -1.234 | -1.523 |
| rno-mir-3551 | 1.245 | 0.962 | 1.048 | 1.257 | 0.997 | 1.102 | 0.062 | 0.861 | 0.907 | 0.939 | 0.861 | 0.892 | 0.019 | 0.023 | 0.810 | -1.235 | -1.525 |
| rno-mir-3590 | 1.143 | 1.434 | 1.250 | 0.997 | 1.282 | 1.221 | 0.073 | 0.814 | 1.119 | 0.938 | 1.047 | 0.979 | 0.067 | 0.048 | 0.802 | -1.247 | -1.555 |
| rno-miR-935 | 1.121 | 1.184 | 1.150 | 1.326 | 0.976 | 1.151 | 0.056 | 0.977 | 1.014 | 0.843 | 0.858 | 0.923 | 0.043 | 0.018 | 0.802 | -1.247 | -1.556 |
| rno-miR-3559-5p | 1.263 | 1.260 | 1.561 | 1.123 | 1.047 | 1.251 | 0.088 | 0.963 | 0.943 | 1.007 | 1.085 | 0.999 | 0.031 | 0.045 | 0.799 | -1.252 | -1.567 |
| rno-mir-293 | 1.146 | 1.274 | 0.975 | 1.006 | 1.006 | 1.081 | 0.056 | 0.670 | 0.823 | 0.968 | 0.975 | 0.859 | 0.072 | 0.043 | 0.794 | -1.259 | -1.584 |
| rno-miR-27a-5p | 5.191 | 4.511 | 4.810 | 3.985 | 4.863 | 4.672 | 0.203 | 4.042 | 4.556 | 3.215 | 2.749 | 3.641 | 0.406 | 0.045 | 0.779 | -1.283 | -1.647 |
| rno-mir-136 | 1.098 | 1.221 | 1.053 | 1.135 | 1.335 | 1.168 | 0.050 | 1.074 | 0.756 | 0.867 | 0.938 | 0.909 | 0.067 | 0.015 | 0.778 | -1.286 | -1.653 |
| rno-mir-34a | 1.617 | 1.318 | 1.624 | 1.391 | 1.434 | 1.477 | 0.062 | 1.131 | 0.999 | 1.474 | 0.958 | 1.141 | 0.117 | 0.030 | 0.772 | -1.295 | -1.677 |
| rno-mir-217 | 1.148 | 1.159 | 1.217 | 1.232 | 1.003 | 1.152 | 0.040 | 0.737 | 1.003 | 0.824 | 0.988 | 0.888 | 0.065 | 0.009 | 0.771 | -1.297 | -1.682 |
| rno-mir-678 | 1.299 | 1.434 | 1.082 | 1.274 | 1.498 | 1.317 | 0.072 | 1.220 | 0.992 | 0.915 | 0.877 | 1.001 | 0.077 | 0.020 | 0.760 | -1.316 | -1.732 |
| rno-miR-23a-5p | 7.277 | 7.511 | 7.379 | 5.837 | 5.549 | 6.710 | 0.420 | 5.135 | 5.076 | 4.900 | 5.275 | 5.097 | 0.078 | 0.012 | 0.760 | -1.317 | -1.733 |
| rno-miR-363-5p | 1.031 | 1.552 | 1.045 | 1.041 | 1.131 | 1.160 | 0.100 | 0.823 | 0.778 | 0.874 | 0.989 | 0.866 | 0.045 | 0.044 | 0.746 | -1.340 | -1.795 |
| rno-miR-802-5p | 1.057 | 1.281 | 1.185 | 1.027 | 0.939 | 1.098 | 0.060 | 0.797 | 0.670 | 0.923 | 0.864 | 0.813 | 0.054 | 0.011 | 0.741 | -1.350 | -1.821 |
| rno-miR-671 | 4.361 | 3.936 | 4.845 | 3.827 | 3.060 | 4.006 | 0.297 | 3.723 | 3.281 | 2.525 | 2.344 | 2.968 | 0.323 | 0.051 | 0.741 | -1.350 | -1.821 |
| rno-miR-344i | 1.297 | 1.434 | 1.474 | 1.132 | 0.900 | 1.247 | 0.105 | 0.922 | 0.907 | 0.877 | 0.965 | 0.918 | 0.018 | 0.029 | 0.736 | -1.359 | -1.847 |
| rno-miR-103-2-5p | 1.029 | 1.331 | 0.956 | 0.956 | 0.957 | 1.046 | 0.073 | 0.756 | 0.649 | 0.953 | 0.710 | 0.767 | 0.066 | 0.028 | 0.734 | -1.363 | -1.857 |
| rno-mir-344a-2 | 1.136 | 1.408 | 1.349 | 0.886 | 1.321 | 1.220 | 0.095 | 0.884 | 1.017 | 0.827 | 0.850 | 0.894 | 0.042 | 0.025 | 0.733 | -1.364 | -1.860 |
| rno-mir-664-1 | 1.739 | 1.279 | 1.547 | 1.291 | 1.356 | 1.442 | 0.088 | 0.906 | 1.099 | 0.960 | 1.201 | 1.041 | 0.067 | 0.011 | 0.722 | -1.385 | -1.918 |
| rno-mir-409a | 1.493 | 1.414 | 1.654 | 1.654 | 1.391 | 1.521 | 0.057 | 1.062 | 1.373 | 0.920 | 0.999 | 1.088 | 0.099 | 0.005 | 0.715 | -1.398 | -1.954 |
| rno-mir-802 | 1.121 | 1.371 | 1.185 | 1.497 | 0.927 | 1.220 | 0.099 | 0.972 | 0.822 | 0.642 | 1.051 | 0.872 | 0.090 | 0.039 | 0.714 | -1.400 | -1.959 |
| rno-miR-153-5p | 1.403 | 1.561 | 0.981 | 0.953 | 1.018 | 1.183 | 0.125 | 0.790 | 0.953 | 0.745 | 0.860 | 0.837 | 0.045 | 0.051 | 0.707 | -1.413 | -1.998 |
| rno-miR-489-5p | 1.143 | 1.640 | 1.257 | 1.137 | 1.066 | 1.248 | 0.102 | 0.749 | 1.016 | 0.816 | 0.924 | 0.876 | 0.059 | 0.022 | 0.702 | -1.425 | -2.030 |
| rno-miR-551b-3p | 0.986 | 1.003 | 1.236 | 1.272 | 1.079 | 1.115 | 0.059 | 0.686 | 0.830 | 0.731 | 0.852 | 0.775 | 0.040 | 0.003 | 0.694 | -1.440 | -2.073 |
| rno-miR-144-3p | 1.640 | 1.315 | 1.408 | 0.989 | 1.197 | 1.310 | 0.108 | 0.793 | 0.855 | 1.017 | 0.797 | 0.865 | 0.052 | 0.012 | 0.661 | -1.513 | -2.290 |
| rno-miR-2985 | 1.046 | 1.041 | 1.418 | 1.019 | 1.155 | 1.136 | 0.074 | 0.859 | 0.873 | 0.683 | 0.582 | 0.749 | 0.071 | 0.008 | 0.660 | -1.516 | -2.298 |
| rno-mir-542 | 1.219 | 1.354 | 1.349 | 0.894 | 1.114 | 1.186 | 0.085 | 0.777 | 0.855 | 0.777 | 0.694 | 0.776 | 0.033 | 0.005 | 0.654 | -1.529 | -2.337 |

| **Supplemental Table 3. Differentially expressed miRNA that were significant (*p*<0.05) in ZO-Rap vs. ZO-C rat hearts.** | | | | | | | | | | | | | | | | | | |
| --- | --- | --- | --- | --- | --- | --- | --- | --- | --- | --- | --- | --- | --- | --- | --- | --- | --- | --- |
|  | ZO-C | ZO-C | ZO-C | ZO-C | ZO-C | AVG | STE | ZO-Rap | ZO-Rap | ZO-Rap | ZO-Rap | ZO-Rap | AVG | STE | p-val | ZO Rap/ ZO-C | Log Ratio | Fold Diff |
| rno-miR-743a-5p | 0.994 | 1.140 | 1.112 | 1.253 | 0.893 | 1.078 | 0.062 | 1.445 | 2.196 | 1.677 | 1.509 | 1.267 | 1.619 | 0.159 | 0.013 | 1.501 | 1.501 | 2.254 |
| rno-miR-219a-1-3p | 0.818 | 1.047 | 1.060 | 1.157 | 1.203 | 1.057 | 0.067 | 1.966 | 1.342 | 1.556 | 1.076 | 1.691 | 1.526 | 0.151 | 0.022 | 1.444 | 1.444 | 2.086 |
| rno-mir-6324 | 0.912 | 1.273 | 1.015 | 1.108 | 0.620 | 0.986 | 0.109 | 1.570 | 1.324 | 1.284 | 1.535 | 1.339 | 1.411 | 0.059 | 0.009 | 1.431 | 1.431 | 2.048 |
| rno-mir-299b | 1.273 | 0.933 | 1.170 | 0.865 | 0.976 | 1.043 | 0.077 | 1.453 | 1.089 | 1.477 | 1.820 | 1.431 | 1.454 | 0.116 | 0.018 | 1.393 | 1.393 | 1.942 |
| rno-mir-299b | 1.342 | 0.951 | 1.199 | 1.043 | 1.063 | 1.120 | 0.068 | 1.192 | 1.596 | 1.581 | 1.866 | 1.477 | 1.542 | 0.109 | 0.011 | 1.378 | 1.378 | 1.898 |
| rno-miR-6318 | 1.641 | 1.328 | 1.638 | 1.193 | 1.330 | 1.426 | 0.091 | 2.360 | 1.658 | 1.606 | 2.344 | 1.773 | 1.948 | 0.167 | 0.025 | 1.366 | 1.366 | 1.867 |
| rno-mir-30b | 0.703 | 0.711 | 1.017 | 0.876 | 1.047 | 0.871 | 0.073 | 1.427 | 1.036 | 1.066 | 1.069 | 1.146 | 1.149 | 0.072 | 0.026 | 1.320 | 1.320 | 1.741 |
| rno-mir-666 | 1.233 | 0.996 | 0.974 | 1.232 | 0.883 | 1.063 | 0.071 | 1.234 | 1.182 | 1.604 | 1.669 | 1.257 | 1.389 | 0.102 | 0.031 | 1.306 | 1.306 | 1.706 |
| rno-miR-292-5p | 0.943 | 0.781 | 0.966 | 0.924 | 0.776 | 0.878 | 0.041 | 1.247 | 1.145 | 0.880 | 1.131 | 1.225 | 1.126 | 0.065 | 0.012 | 1.282 | 1.282 | 1.643 |
| rno-miR-370-5p | 0.775 | 0.811 | 0.717 | 1.019 | 0.756 | 0.816 | 0.053 | 1.209 | 0.900 | 1.024 | 0.898 | 1.144 | 1.035 | 0.063 | 0.029 | 1.269 | 1.269 | 1.610 |
| rno-miR-448-3p | 1.233 | 0.841 | 0.963 | 0.995 | 0.959 | 0.998 | 0.064 | 1.458 | 1.176 | 1.179 | 1.469 | 1.018 | 1.260 | 0.088 | 0.043 | 1.262 | 1.262 | 1.593 |
| rno-mir-9a-1 | 1.225 | 0.704 | 1.136 | 1.078 | 1.036 | 1.036 | 0.089 | 1.283 | 1.471 | 1.352 | 1.146 | 1.215 | 1.293 | 0.056 | 0.040 | 1.249 | 1.249 | 1.560 |
| rno-mir-3577 | 1.021 | 1.093 | 1.098 | 1.256 | 0.779 | 1.050 | 0.078 | 1.234 | 1.238 | 1.135 | 1.445 | 1.474 | 1.305 | 0.066 | 0.036 | 1.244 | 1.244 | 1.547 |
| rno-mir-3579 | 1.047 | 0.925 | 0.689 | 0.975 | 0.743 | 0.876 | 0.069 | 1.003 | 1.062 | 1.062 | 1.262 | 1.019 | 1.082 | 0.046 | 0.038 | 1.235 | 1.235 | 1.525 |
| rno-mir-93 | 1.902 | 1.677 | 1.851 | 1.961 | 1.215 | 1.721 | 0.135 | 1.983 | 2.027 | 2.230 | 2.434 | 1.898 | 2.114 | 0.097 | 0.046 | 1.228 | 1.228 | 1.509 |
| rno-mir-20b | 1.609 | 1.677 | 1.473 | 1.908 | 1.345 | 1.602 | 0.095 | 2.043 | 1.650 | 1.915 | 2.097 | 2.134 | 1.968 | 0.088 | 0.022 | 1.228 | 1.228 | 1.508 |
| rno-miR-298-5p | 4.243 | 4.861 | 5.413 | 5.290 | 5.768 | 5.115 | 0.262 | 6.551 | 6.472 | 6.119 | 5.628 | 6.579 | 6.270 | 0.180 | 0.007 | 1.226 | 1.226 | 1.502 |
| rno-mir-500 | 1.673 | 1.403 | 1.372 | 1.493 | 1.495 | 1.487 | 0.052 | 2.082 | 1.669 | 1.811 | 1.664 | 1.885 | 1.822 | 0.077 | 0.007 | 1.225 | 1.225 | 1.502 |
| rno-miR-330-3p | 4.715 | 4.830 | 3.717 | 5.013 | 5.298 | 4.715 | 0.268 | 6.347 | 5.735 | 5.438 | 5.158 | 5.941 | 5.724 | 0.205 | 0.017 | 1.214 | 1.214 | 1.474 |
| rno-mir-410 | 0.876 | 1.073 | 0.783 | 0.990 | 0.872 | 0.919 | 0.051 | 1.165 | 0.993 | 1.282 | 1.165 | 0.963 | 1.113 | 0.060 | 0.038 | 1.212 | 1.212 | 1.468 |
| rno-mir-3585 | 1.659 | 1.329 | 1.553 | 1.363 | 1.141 | 1.409 | 0.090 | 1.530 | 1.726 | 1.874 | 1.697 | 1.688 | 1.703 | 0.055 | 0.024 | 1.209 | 1.209 | 1.461 |
| rno-miR-340-5p | 1.132 | 1.114 | 0.956 | 1.141 | 1.212 | 1.111 | 0.042 | 1.361 | 1.264 | 1.302 | 1.116 | 1.660 | 1.341 | 0.090 | 0.049 | 1.207 | 1.207 | 1.456 |
| rno-mir-181c | 1.422 | 1.337 | 1.345 | 1.549 | 1.467 | 1.424 | 0.040 | 1.929 | 1.664 | 1.572 | 1.965 | 1.446 | 1.715 | 0.101 | 0.028 | 1.205 | 1.205 | 1.451 |
| rno-miR-3552 | 0.949 | 1.179 | 1.037 | 0.997 | 1.201 | 1.073 | 0.050 | 1.411 | 1.211 | 1.461 | 1.222 | 1.139 | 1.289 | 0.062 | 0.027 | 1.202 | 1.202 | 1.444 |
| rno-mir-186 | 0.991 | 1.078 | 1.096 | 1.066 | 0.944 | 1.035 | 0.029 | 1.096 | 1.181 | 1.212 | 1.430 | 1.257 | 1.235 | 0.055 | 0.012 | 1.193 | 1.193 | 1.424 |
| rno-miR-17-1-3p | 5.881 | 6.115 | 6.241 | 6.343 | 4.942 | 5.904 | 0.253 | 7.536 | 6.284 | 7.482 | 7.039 | 6.847 | 7.038 | 0.229 | 0.011 | 1.192 | 1.192 | 1.421 |
| rno-mir-383 | 2.072 | 1.921 | 1.814 | 1.959 | 1.764 | 1.906 | 0.054 | 2.480 | 2.439 | 1.985 | 2.196 | 2.214 | 2.263 | 0.090 | 0.009 | 1.187 | 1.187 | 1.410 |
| rno-let-7f-2 | 0.781 | 0.701 | 0.845 | 0.924 | 0.971 | 0.844 | 0.048 | 0.995 | 0.898 | 1.009 | 1.132 | 0.972 | 1.001 | 0.038 | 0.034 | 1.186 | 1.186 | 1.407 |
| rno-mir-216a | 1.021 | 1.023 | 1.275 | 0.977 | 0.929 | 1.045 | 0.060 | 1.300 | 1.284 | 1.184 | 1.120 | 1.257 | 1.229 | 0.034 | 0.028 | 1.176 | 1.176 | 1.383 |
| rno-miR-490-3p | 6.655 | 6.815 | 6.650 | 6.751 | 5.500 | 6.475 | 0.245 | 8.181 | 6.720 | 8.048 | 7.716 | 7.363 | 7.606 | 0.263 | 0.014 | 1.175 | 1.175 | 1.380 |
| rno-miR-504 | 6.765 | 6.994 | 6.788 | 6.755 | 5.381 | 6.537 | 0.292 | 7.791 | 7.371 | 7.724 | 7.406 | 7.729 | 7.604 | 0.089 | 0.008 | 1.163 | 1.163 | 1.353 |
| rno-mir-384 | 0.757 | 0.882 | 0.940 | 0.882 | 0.999 | 0.892 | 0.040 | 0.958 | 1.125 | 1.156 | 0.977 | 0.968 | 1.037 | 0.043 | 0.039 | 1.162 | 1.162 | 1.351 |
| rno-miR-325-3p | 1.113 | 1.098 | 1.029 | 1.102 | 1.105 | 1.089 | 0.015 | 1.216 | 1.443 | 1.298 | 1.003 | 1.364 | 1.265 | 0.075 | 0.052 | 1.161 | 1.161 | 1.349 |
| rno-mir-532 | 1.580 | 1.390 | 1.572 | 1.555 | 1.269 | 1.473 | 0.062 | 1.743 | 1.579 | 1.545 | 1.765 | 1.912 | 1.709 | 0.067 | 0.032 | 1.160 | 1.160 | 1.345 |
| rno-miR-96-3p | 1.682 | 1.785 | 1.621 | 1.706 | 1.704 | 1.699 | 0.026 | 1.797 | 1.943 | 1.877 | 2.085 | 2.128 | 1.966 | 0.062 | 0.004 | 1.157 | 1.157 | 1.338 |
| rno-miR-3557-5p | 0.942 | 0.837 | 1.025 | 0.816 | 1.089 | 0.942 | 0.053 | 1.093 | 1.132 | 0.968 | 1.125 | 1.121 | 1.088 | 0.031 | 0.044 | 1.155 | 1.155 | 1.333 |
| rno-miR-346 | 6.861 | 6.298 | 6.722 | 7.160 | 5.827 | 6.574 | 0.232 | 7.589 | 7.285 | 7.850 | 7.759 | 7.324 | 7.561 | 0.113 | 0.005 | 1.150 | 1.150 | 1.323 |
| rno-mir-758 | 0.971 | 1.029 | 1.033 | 0.973 | 0.996 | 1.000 | 0.013 | 1.060 | 1.146 | 1.297 | 1.202 | 1.011 | 1.143 | 0.051 | 0.026 | 1.143 | 1.143 | 1.306 |
| rno-miR-25-5p | 6.755 | 6.810 | 6.976 | 6.832 | 7.440 | 6.963 | 0.125 | 8.008 | 8.052 | 7.875 | 7.505 | 8.126 | 7.913 | 0.110 | 0.000 | 1.137 | 1.137 | 1.292 |
| rno-miR-1306-3p | 4.505 | 4.256 | 4.192 | 4.657 | 4.514 | 4.425 | 0.087 | 4.713 | 5.610 | 5.468 | 4.461 | 4.878 | 5.026 | 0.221 | 0.035 | 1.136 | 1.136 | 1.290 |
| rno-miR-138-5p | 5.909 | 6.212 | 6.469 | 6.507 | 5.740 | 6.167 | 0.151 | 7.686 | 6.355 | 7.043 | 6.719 | 7.185 | 6.998 | 0.224 | 0.015 | 1.135 | 1.135 | 1.287 |
| rno-miR-99b-3p | 6.174 | 6.182 | 6.526 | 6.210 | 6.007 | 6.220 | 0.084 | 7.301 | 6.946 | 7.112 | 6.658 | 6.875 | 6.979 | 0.109 | 0.001 | 1.122 | 1.122 | 1.259 |
| rno-miR-874-5p | 2.052 | 2.252 | 2.170 | 1.950 | 1.820 | 2.049 | 0.077 | 2.492 | 2.219 | 2.156 | 2.154 | 2.470 | 2.298 | 0.076 | 0.050 | 1.122 | 1.122 | 1.258 |
| rno-miR-423-3p | 8.562 | 8.584 | 8.558 | 8.240 | 7.756 | 8.340 | 0.159 | 9.615 | 9.079 | 9.482 | 9.174 | 9.325 | 9.335 | 0.098 | 0.001 | 1.119 | 1.119 | 1.253 |
| rno-miR-423-5p | 8.126 | 8.150 | 7.823 | 7.958 | 9.602 | 8.332 | 0.323 | 9.397 | 9.676 | 9.295 | 8.873 | 9.283 | 9.305 | 0.129 | 0.023 | 1.117 | 1.117 | 1.247 |
| rno-miR-324-3p | 7.274 | 7.332 | 7.266 | 7.456 | 6.304 | 7.127 | 0.208 | 7.997 | 7.439 | 8.179 | 7.735 | 8.050 | 7.880 | 0.132 | 0.016 | 1.106 | 1.106 | 1.223 |
| rno-miR-500-3p | 8.243 | 8.150 | 8.285 | 8.325 | 8.266 | 8.254 | 0.029 | 9.135 | 9.154 | 9.305 | 8.995 | 8.977 | 9.113 | 0.060 | 0.000 | 1.104 | 1.104 | 1.219 |
| rno-mir-423 | 6.233 | 6.371 | 6.252 | 6.280 | 5.670 | 6.161 | 0.125 | 6.977 | 6.323 | 6.967 | 6.824 | 6.684 | 6.755 | 0.121 | 0.009 | 1.096 | 1.096 | 1.202 |
| rno-miR-210-3p | 9.340 | 9.187 | 9.108 | 9.215 | 8.598 | 9.090 | 0.128 | 10.488 | 9.354 | 10.412 | 10.080 | 9.486 | 9.964 | 0.233 | 0.011 | 1.096 | 1.096 | 1.202 |
| rno-miR-139-3p | 6.162 | 6.375 | 6.055 | 6.083 | 6.319 | 6.199 | 0.064 | 6.931 | 7.070 | 6.621 | 6.270 | 7.044 | 6.787 | 0.152 | 0.007 | 1.095 | 1.095 | 1.199 |
| rno-miR-532-5p | 8.106 | 7.903 | 8.185 | 7.999 | 7.576 | 7.954 | 0.106 | 8.821 | 8.381 | 8.997 | 8.645 | 8.590 | 8.687 | 0.105 | 0.001 | 1.092 | 1.092 | 1.193 |
| rno-miR-106b-3p | 7.433 | 7.360 | 7.410 | 7.644 | 6.688 | 7.307 | 0.162 | 8.177 | 7.584 | 8.211 | 7.741 | 8.181 | 7.979 | 0.132 | 0.012 | 1.092 | 1.092 | 1.192 |
| rno-miR-339-3p | 7.849 | 7.713 | 7.680 | 7.850 | 7.630 | 7.744 | 0.045 | 8.697 | 8.372 | 8.614 | 8.286 | 8.171 | 8.428 | 0.099 | 0.000 | 1.088 | 1.088 | 1.184 |
| rno-miR-542-5p | 6.674 | 6.594 | 6.437 | 6.148 | 6.036 | 6.378 | 0.124 | 7.430 | 6.636 | 6.470 | 7.060 | 7.087 | 6.937 | 0.172 | 0.030 | 1.088 | 1.088 | 1.183 |
| rno-miR-125b-2-3p | 8.662 | 8.391 | 8.498 | 8.598 | 8.096 | 8.449 | 0.100 | 9.339 | 8.813 | 9.460 | 9.096 | 9.119 | 9.165 | 0.111 | 0.001 | 1.085 | 1.085 | 1.177 |
| rno-miR-34c-3p | 5.163 | 4.803 | 5.070 | 5.195 | 5.535 | 5.153 | 0.118 | 5.690 | 5.807 | 5.251 | 5.243 | 5.832 | 5.564 | 0.132 | 0.048 | 1.080 | 1.080 | 1.166 |
| rno-mir-351 | 5.952 | 5.752 | 5.635 | 5.976 | 5.514 | 5.766 | 0.089 | 6.177 | 6.250 | 6.274 | 6.237 | 6.178 | 6.223 | 0.020 | 0.001 | 1.079 | 1.079 | 1.165 |
| rno-miR-532-3p | 8.251 | 8.189 | 8.342 | 8.616 | 8.199 | 8.319 | 0.079 | 8.649 | 8.884 | 9.328 | 8.796 | 9.132 | 8.958 | 0.121 | 0.002 | 1.077 | 1.077 | 1.159 |
| rno-miR-652-5p | 5.714 | 6.122 | 5.392 | 5.655 | 5.819 | 5.740 | 0.119 | 6.464 | 6.437 | 5.938 | 5.911 | 6.132 | 6.177 | 0.118 | 0.031 | 1.076 | 1.076 | 1.158 |
| rno-miR-93-5p | 10.083 | 9.737 | 9.832 | 10.122 | 9.374 | 9.829 | 0.135 | 10.773 | 9.963 | 10.795 | 10.647 | 10.469 | 10.529 | 0.153 | 0.009 | 1.071 | 1.071 | 1.147 |
| rno-miR-503-5p | 8.311 | 7.944 | 8.124 | 7.872 | 7.594 | 7.969 | 0.121 | 8.945 | 7.930 | 8.865 | 8.340 | 8.568 | 8.530 | 0.185 | 0.035 | 1.070 | 1.070 | 1.146 |
| rno-miR-214-3p | 10.198 | 9.751 | 10.070 | 10.127 | 11.046 | 10.238 | 0.216 | 10.954 | 11.151 | 10.992 | 10.630 | 11.031 | 10.952 | 0.087 | 0.015 | 1.070 | 1.070 | 1.144 |
| rno-miR-34a-5p | 9.381 | 9.617 | 9.604 | 9.511 | 9.791 | 9.581 | 0.067 | 10.714 | 9.932 | 10.276 | 10.319 | 9.942 | 10.237 | 0.144 | 0.003 | 1.068 | 1.068 | 1.142 |
| rno-miR-378a-5p | 9.950 | 9.908 | 9.789 | 9.799 | 8.992 | 9.688 | 0.177 | 10.691 | 9.746 | 10.780 | 10.422 | 10.103 | 10.348 | 0.191 | 0.035 | 1.068 | 1.068 | 1.141 |
| rno-miR-503-3p | 7.473 | 7.205 | 7.210 | 7.158 | 6.406 | 7.090 | 0.180 | 7.676 | 7.450 | 7.671 | 7.348 | 7.705 | 7.570 | 0.072 | 0.038 | 1.068 | 1.068 | 1.140 |
| rno-miR-328a-3p | 9.908 | 9.828 | 9.884 | 9.509 | 9.655 | 9.757 | 0.076 | 10.232 | 10.656 | 10.453 | 10.099 | 10.438 | 10.376 | 0.096 | 0.001 | 1.063 | 1.063 | 1.131 |
| rno-miR-351-5p | 9.899 | 9.760 | 9.805 | 9.945 | 9.169 | 9.716 | 0.141 | 10.362 | 10.270 | 10.374 | 10.299 | 10.277 | 10.316 | 0.022 | 0.003 | 1.062 | 1.062 | 1.127 |
| rno-miR-99b-5p | 12.033 | 11.979 | 11.926 | 12.181 | 11.555 | 11.935 | 0.104 | 12.526 | 12.478 | 12.684 | 12.568 | 12.649 | 12.581 | 0.038 | 0.000 | 1.054 | 1.054 | 1.111 |
| rno-miR-140-3p | 11.012 | 10.409 | 10.998 | 11.196 | 10.360 | 10.795 | 0.171 | 11.272 | 11.146 | 12.014 | 11.451 | 10.962 | 11.369 | 0.180 | 0.050 | 1.053 | 1.053 | 1.109 |
| rno-miR-17-5p | 10.759 | 10.690 | 10.795 | 10.885 | 10.064 | 10.639 | 0.147 | 11.506 | 10.723 | 11.378 | 11.242 | 10.988 | 11.167 | 0.140 | 0.032 | 1.050 | 1.050 | 1.102 |
| rno-miR-181b-5p | 10.677 | 10.443 | 10.670 | 10.750 | 10.750 | 10.658 | 0.056 | 11.084 | 11.411 | 11.287 | 11.050 | 11.010 | 11.168 | 0.077 | 0.001 | 1.048 | 1.048 | 1.098 |
| rno-mir-652 | 5.267 | 5.438 | 5.234 | 5.177 | 5.483 | 5.320 | 0.060 | 5.620 | 5.500 | 5.722 | 5.503 | 5.523 | 5.574 | 0.043 | 0.009 | 1.048 | 1.048 | 1.098 |
| rno-miR-30c-2-3p | 8.980 | 8.886 | 8.966 | 8.866 | 9.595 | 9.059 | 0.136 | 9.500 | 9.722 | 9.558 | 9.175 | 9.482 | 9.488 | 0.089 | 0.030 | 1.047 | 1.047 | 1.097 |
| rno-miR-652-3p | 10.750 | 10.661 | 10.620 | 10.557 | 11.085 | 10.735 | 0.093 | 11.286 | 11.374 | 11.207 | 10.981 | 11.308 | 11.232 | 0.068 | 0.003 | 1.046 | 1.046 | 1.095 |
| rno-miR-361-5p | 11.198 | 11.100 | 11.249 | 11.332 | 11.743 | 11.324 | 0.111 | 11.797 | 11.955 | 11.695 | 11.563 | 11.914 | 11.785 | 0.072 | 0.008 | 1.041 | 1.041 | 1.083 |
| rno-miR-20a-5p | 10.560 | 10.569 | 10.589 | 10.625 | 10.109 | 10.491 | 0.096 | 11.142 | 10.499 | 11.122 | 10.897 | 10.807 | 10.893 | 0.118 | 0.029 | 1.038 | 1.038 | 1.078 |
| rno-miR-103-3p | 13.073 | 12.970 | 13.025 | 13.148 | 12.866 | 13.016 | 0.048 | 13.597 | 13.360 | 13.571 | 13.554 | 13.255 | 13.467 | 0.068 | 0.001 | 1.035 | 1.035 | 1.070 |
| rno-miR-181a-5p | 11.852 | 11.832 | 11.824 | 11.913 | 11.426 | 11.769 | 0.087 | 12.271 | 11.848 | 12.266 | 12.225 | 12.113 | 12.145 | 0.079 | 0.013 | 1.032 | 1.032 | 1.065 |
| rno-miR-378a-3p | 13.395 | 13.321 | 13.453 | 13.454 | 13.456 | 13.416 | 0.026 | 13.729 | 14.089 | 13.839 | 13.727 | 13.619 | 13.801 | 0.080 | 0.002 | 1.029 | 1.029 | 1.058 |
| rno-miR-107-3p | 12.880 | 12.777 | 12.891 | 12.901 | 12.591 | 12.808 | 0.059 | 13.320 | 12.986 | 13.218 | 13.302 | 12.952 | 13.156 | 0.078 | 0.007 | 1.027 | 1.027 | 1.055 |
| rno-miR-28-3p | 8.847 | 8.649 | 8.898 | 8.828 | 8.806 | 8.806 | 0.042 | 8.948 | 9.283 | 9.081 | 8.793 | 9.114 | 9.044 | 0.082 | 0.033 | 1.027 | 1.027 | 1.055 |
| rno-miR-92a-3p | 11.044 | 11.046 | 11.228 | 11.030 | 11.261 | 11.122 | 0.050 | 11.366 | 11.526 | 11.309 | 11.071 | 11.730 | 11.400 | 0.110 | 0.050 | 1.025 | 1.025 | 1.051 |
| rno-miR-191a-5p | 13.630 | 13.737 | 13.661 | 13.647 | 13.550 | 13.645 | 0.030 | 13.935 | 14.054 | 13.910 | 13.864 | 14.000 | 13.952 | 0.034 | 0.000 | 1.023 | 1.023 | 1.046 |
| rno-miR-24-3p | 14.583 | 14.656 | 14.588 | 14.578 | 14.233 | 14.528 | 0.075 | 14.963 | 14.658 | 14.879 | 14.818 | 14.814 | 14.826 | 0.050 | 0.011 | 1.021 | 1.021 | 1.042 |
| AFFX-r2-P1-c1-cre-3 | 15.391 | 15.377 | 15.387 | 15.454 | 15.532 | 15.428 | 0.029 | 15.258 | 15.345 | 15.353 | 15.381 | 15.255 | 15.319 | 0.026 | 0.023 | 0.993 | -1.007 | -1.014 |
| rno-miR-221-3p | 9.984 | 10.096 | 10.188 | 10.156 | 9.889 | 10.063 | 0.056 | 9.835 | 9.664 | 10.010 | 9.648 | 9.925 | 9.817 | 0.071 | 0.026 | 0.976 | -1.025 | -1.051 |
| rno-miR-195-5p | 12.522 | 12.239 | 12.481 | 12.598 | 12.411 | 12.450 | 0.061 | 12.127 | 11.974 | 11.998 | 11.942 | 12.221 | 12.052 | 0.053 | 0.001 | 0.968 | -1.033 | -1.067 |
| rno-let-7a-5p | 14.013 | 14.274 | 14.306 | 14.220 | 14.701 | 14.303 | 0.112 | 13.571 | 14.173 | 13.451 | 13.523 | 14.149 | 13.773 | 0.159 | 0.026 | 0.963 | -1.038 | -1.078 |
| rno-miR-146b-5p | 8.536 | 8.927 | 8.583 | 8.628 | 8.628 | 8.660 | 0.069 | 8.283 | 8.081 | 8.262 | 8.429 | 8.446 | 8.300 | 0.066 | 0.006 | 0.958 | -1.043 | -1.089 |
| rno-let-7i-5p | 12.693 | 12.716 | 12.962 | 12.645 | 13.375 | 12.878 | 0.136 | 12.169 | 12.571 | 12.036 | 11.947 | 12.664 | 12.278 | 0.144 | 0.016 | 0.953 | -1.049 | -1.100 |
| rno-mir-1839 | 3.780 | 3.912 | 3.921 | 3.977 | 4.071 | 3.932 | 0.047 | 3.859 | 3.565 | 3.695 | 3.678 | 3.893 | 3.738 | 0.061 | 0.036 | 0.951 | -1.052 | -1.106 |
| rno-miR-1-3p | 13.197 | 13.198 | 13.414 | 13.257 | 13.915 | 13.396 | 0.136 | 12.460 | 13.126 | 12.348 | 12.563 | 13.017 | 12.703 | 0.155 | 0.010 | 0.948 | -1.055 | -1.112 |
| rno-miR-30e-3p | 8.480 | 8.066 | 8.532 | 8.493 | 7.843 | 8.283 | 0.139 | 7.494 | 7.832 | 7.871 | 7.388 | 8.056 | 7.728 | 0.124 | 0.018 | 0.933 | -1.072 | -1.149 |
| rno-miR-199a-3p | 11.480 | 11.295 | 11.731 | 11.438 | 11.496 | 11.488 | 0.070 | 10.562 | 10.386 | 10.598 | 10.480 | 11.356 | 10.676 | 0.174 | 0.003 | 0.929 | -1.076 | -1.158 |
| rno-miR-224-5p | 7.530 | 7.437 | 8.079 | 7.783 | 7.772 | 7.720 | 0.112 | 7.205 | 7.046 | 6.911 | 7.092 | 7.580 | 7.167 | 0.114 | 0.008 | 0.928 | -1.077 | -1.160 |
| rno-let-7f-5p | 11.490 | 11.612 | 11.747 | 11.506 | 12.950 | 11.861 | 0.276 | 10.597 | 11.626 | 10.295 | 10.733 | 11.577 | 10.965 | 0.269 | 0.049 | 0.924 | -1.082 | -1.170 |
| rno-miR-15b-5p | 10.063 | 9.708 | 10.382 | 10.455 | 10.442 | 10.210 | 0.144 | 9.256 | 9.434 | 9.290 | 9.159 | 9.919 | 9.412 | 0.134 | 0.004 | 0.922 | -1.085 | -1.177 |
| rno-miR-3473 | 9.828 | 10.974 | 9.276 | 9.569 | 9.285 | 9.786 | 0.314 | 9.122 | 8.491 | 8.152 | 8.756 | 9.426 | 8.789 | 0.225 | 0.033 | 0.898 | -1.113 | -1.240 |
| rno-miR-350 | 7.186 | 7.194 | 6.957 | 7.430 | 6.422 | 7.038 | 0.171 | 6.736 | 5.650 | 6.397 | 5.996 | 6.726 | 6.301 | 0.212 | 0.027 | 0.895 | -1.117 | -1.248 |
| rno-miR-146a-5p | 11.112 | 11.402 | 11.437 | 11.378 | 11.811 | 11.428 | 0.112 | 9.946 | 10.323 | 10.145 | 10.132 | 10.540 | 10.217 | 0.100 | 0.000 | 0.894 | -1.119 | -1.251 |
| rno-miR-10a-5p | 8.065 | 7.832 | 7.977 | 7.702 | 8.190 | 7.953 | 0.086 | 6.812 | 7.151 | 7.304 | 6.866 | 7.239 | 7.074 | 0.099 | 0.000 | 0.890 | -1.124 | -1.264 |
| rno-miR-3584-5p | 6.554 | 6.378 | 6.244 | 5.811 | 6.966 | 6.391 | 0.189 | 5.824 | 5.797 | 5.139 | 5.436 | 6.123 | 5.664 | 0.170 | 0.021 | 0.886 | -1.128 | -1.273 |
| rno-mir-219a-2 | 1.075 | 0.933 | 1.079 | 1.044 | 0.892 | 1.005 | 0.039 | 0.960 | 0.874 | 0.934 | 0.873 | 0.740 | 0.876 | 0.038 | 0.045 | 0.872 | -1.147 | -1.315 |
| rno-miR-6215 | 9.513 | 10.732 | 8.739 | 8.977 | 9.603 | 9.513 | 0.345 | 8.085 | 8.217 | 7.545 | 8.110 | 9.025 | 8.197 | 0.238 | 0.014 | 0.862 | -1.161 | -1.347 |
| rno-miR-155-5p | 6.770 | 7.341 | 7.313 | 7.303 | 7.939 | 7.333 | 0.185 | 6.156 | 6.556 | 5.538 | 5.811 | 7.408 | 6.294 | 0.327 | 0.024 | 0.858 | -1.165 | -1.358 |
| rno-mir-125a | 2.024 | 1.734 | 1.668 | 1.697 | 1.915 | 1.807 | 0.069 | 1.272 | 1.468 | 1.556 | 1.512 | 1.836 | 1.529 | 0.091 | 0.040 | 0.846 | -1.182 | -1.397 |
| rno-mir-10b | 1.001 | 1.193 | 0.970 | 1.167 | 1.033 | 1.073 | 0.045 | 0.812 | 0.898 | 0.968 | 0.830 | 1.025 | 0.906 | 0.040 | 0.025 | 0.845 | -1.183 | -1.401 |
| rno-mir-202 | 1.086 | 0.940 | 0.991 | 1.177 | 1.268 | 1.092 | 0.060 | 0.825 | 0.816 | 0.973 | 0.898 | 1.029 | 0.908 | 0.041 | 0.035 | 0.832 | -1.203 | -1.446 |
| rno-miR-10b-5p | 2.121 | 2.012 | 2.270 | 2.113 | 1.821 | 2.067 | 0.074 | 1.581 | 1.732 | 1.601 | 1.529 | 2.094 | 1.707 | 0.102 | 0.022 | 0.826 | -1.211 | -1.466 |
| rno-miR-672-3p | 1.250 | 1.436 | 1.138 | 1.015 | 1.144 | 1.196 | 0.071 | 1.179 | 0.823 | 0.871 | 1.030 | 0.942 | 0.969 | 0.063 | 0.043 | 0.810 | -1.235 | -1.525 |
| rno-miR-3574 | 1.288 | 1.600 | 1.708 | 1.435 | 1.401 | 1.486 | 0.074 | 1.337 | 0.892 | 1.255 | 1.374 | 1.143 | 1.200 | 0.087 | 0.037 | 0.808 | -1.238 | -1.534 |
| rno-miR-3549 | 1.021 | 1.427 | 1.147 | 1.091 | 1.175 | 1.172 | 0.069 | 0.895 | 1.104 | 0.886 | 0.989 | 0.857 | 0.946 | 0.045 | 0.025 | 0.807 | -1.239 | -1.535 |
| rno-miR-292-3p | 1.040 | 0.929 | 1.159 | 1.065 | 1.105 | 1.059 | 0.038 | 0.711 | 0.944 | 0.695 | 0.960 | 0.958 | 0.853 | 0.062 | 0.022 | 0.806 | -1.241 | -1.541 |
| rno-mir-382 | 1.081 | 1.292 | 1.288 | 1.310 | 1.366 | 1.267 | 0.049 | 1.127 | 1.062 | 0.897 | 1.162 | 0.841 | 1.018 | 0.063 | 0.014 | 0.803 | -1.245 | -1.551 |
| rno-mir-802 | 1.010 | 1.341 | 1.140 | 1.424 | 1.515 | 1.286 | 0.093 | 0.999 | 1.039 | 1.043 | 0.974 | 1.103 | 1.032 | 0.022 | 0.028 | 0.802 | -1.247 | -1.554 |
| rno-mir-216b | 1.252 | 1.408 | 1.100 | 1.529 | 1.304 | 1.319 | 0.072 | 0.922 | 0.978 | 1.097 | 1.159 | 1.109 | 1.053 | 0.044 | 0.014 | 0.799 | -1.252 | -1.568 |
| rno-miR-221-5p | 2.513 | 1.986 | 2.213 | 1.882 | 2.054 | 2.129 | 0.110 | 2.224 | 1.503 | 1.602 | 1.434 | 1.691 | 1.691 | 0.140 | 0.039 | 0.794 | -1.259 | -1.586 |
| rno-miR-7a-5p | 1.515 | 1.752 | 1.880 | 1.434 | 1.147 | 1.545 | 0.128 | 1.132 | 1.281 | 1.271 | 1.350 | 1.088 | 1.224 | 0.049 | 0.047 | 0.792 | -1.262 | -1.593 |
| rno-mir-191b | 1.450 | 1.698 | 1.547 | 1.462 | 1.836 | 1.598 | 0.074 | 1.584 | 1.069 | 1.308 | 1.303 | 1.024 | 1.258 | 0.100 | 0.026 | 0.787 | -1.271 | -1.616 |
| rno-miR-301a-5p | 1.065 | 1.069 | 0.918 | 0.807 | 1.194 | 1.010 | 0.067 | 0.707 | 0.743 | 0.718 | 0.777 | 1.029 | 0.795 | 0.060 | 0.043 | 0.787 | -1.271 | -1.617 |
| rno-miR-3559-5p | 0.982 | 1.442 | 1.064 | 1.143 | 1.305 | 1.187 | 0.083 | 1.082 | 0.743 | 0.905 | 0.859 | 1.064 | 0.931 | 0.064 | 0.040 | 0.784 | -1.276 | -1.628 |
| rno-miR-129-2-3p | 1.637 | 1.905 | 1.495 | 1.545 | 1.630 | 1.642 | 0.071 | 0.901 | 1.257 | 1.247 | 1.197 | 1.740 | 1.269 | 0.135 | 0.040 | 0.772 | -1.295 | -1.676 |
| rno-miR-434-3p | 5.097 | 4.930 | 6.290 | 5.815 | 5.341 | 5.495 | 0.249 | 4.170 | 4.070 | 3.658 | 3.440 | 5.852 | 4.238 | 0.425 | 0.034 | 0.771 | -1.297 | -1.681 |
| rno-miR-770-3p | 1.214 | 1.702 | 1.297 | 1.226 | 1.352 | 1.358 | 0.090 | 1.129 | 1.181 | 0.717 | 0.981 | 1.078 | 1.017 | 0.082 | 0.023 | 0.749 | -1.335 | -1.782 |
| rno-miR-6317 | 1.415 | 1.314 | 1.616 | 1.257 | 1.295 | 1.379 | 0.065 | 0.997 | 0.789 | 1.234 | 1.189 | 0.924 | 1.026 | 0.083 | 0.010 | 0.744 | -1.344 | -1.806 |
| rno-miR-1843-3p | 3.369 | 4.203 | 3.605 | 3.231 | 3.525 | 3.587 | 0.167 | 1.413 | 1.787 | 2.222 | 1.774 | 4.106 | 2.260 | 0.479 | 0.031 | 0.630 | -1.587 | -2.518 |
| rno-miR-511-3p | 4.199 | 4.545 | 4.676 | 4.577 | 2.488 | 4.097 | 0.410 | 2.916 | 2.505 | 1.699 | 2.546 | 2.185 | 2.370 | 0.204 | 0.005 | 0.579 | -1.729 | -2.988 |
| rno-miR-409b | 1.977 | 2.587 | 1.527 | 1.606 | 0.997 | 1.739 | 0.264 | 0.764 | 1.198 | 0.801 | 1.051 | 0.853 | 0.933 | 0.083 | 0.019 | 0.537 | -1.863 | -3.471 |

**Supplemental Table 4. Differentially expressed miRNA that were significant (*p*<0.05) in ZO-Rap vs. ZO-C rat hearts.**

| \|  \| ZL-C \| ZL-C \| ZL-C \| ZL-C \| ZL-C \| Avg \| STE \| ZO-Rap \| ZO-Rap \| ZO-Rap \| ZO-Rap \| ZO-Rap \| Avg \| STE \| P-value \| ZO-Rap/ZL-C \| Log Ratio \| Fold Diff \| \| --- \| --- \| --- \| --- \| --- \| --- \| --- \| --- \| --- \| --- \| --- \| --- \| --- \| --- \| --- \| --- \| --- \| --- \| --- \| \| rno-miR-200c-3p \| 4.044 \| 2.092 \| 2.495 \| 4.670 \| 5.514 \| 3.763 \| 0.647 \| 8.668 \| 8.901 \| 8.271 \| 7.941 \| 8.307 \| 8.418 \| 0.373 \| 0.000 \| 2.237 \| 2.237 \| 4.714 \| \| rno-miR-141-3p \| 1.275 \| 1.701 \| 1.052 \| 2.115 \| 1.519 \| 1.532 \| 0.182 \| 6.014 \| 1.296 \| 4.043 \| 2.963 \| 2.652 \| 3.394 \| 1.762 \| 0.050 \| 2.215 \| 2.215 \| 4.641 \| \| rno-miR-7a-1-3p \| 1.144 \| 1.156 \| 0.976 \| 2.555 \| 3.566 \| 1.879 \| 0.509 \| 4.419 \| 3.506 \| 4.522 \| 4.019 \| 4.223 \| 4.138 \| 0.402 \| 0.003 \| 2.202 \| 2.202 \| 4.600 \| \| rno-miR-138-1-3p \| 1.752 \| 1.045 \| 1.380 \| 3.928 \| 2.043 \| 2.030 \| 0.504 \| 3.715 \| 4.297 \| 4.423 \| 5.391 \| 4.454 \| 4.456 \| 0.602 \| 0.003 \| 2.196 \| 2.196 \| 4.581 \| \| rno-miR-21-5p \| 1.051 \| 1.012 \| 0.984 \| 5.808 \| 7.072 \| 3.185 \| 1.344 \| 7.067 \| 7.291 \| 6.376 \| 6.028 \| 7.967 \| 6.946 \| 0.766 \| 0.027 \| 2.181 \| 2.181 \| 4.533 \| \| rno-miR-26b-5p \| 1.382 \| 1.204 \| 1.169 \| 5.953 \| 6.958 \| 3.333 \| 1.285 \| 7.102 \| 7.375 \| 6.680 \| 6.797 \| 7.824 \| 7.156 \| 0.462 \| 0.019 \| 2.147 \| 2.147 \| 4.428 \| \| rno-miR-434-3p \| 1.954 \| 1.021 \| 1.197 \| 2.565 \| 3.260 \| 2.000 \| 0.419 \| 4.170 \| 4.070 \| 3.658 \| 3.440 \| 5.852 \| 4.238 \| 0.950 \| 0.006 \| 2.119 \| 2.119 \| 4.345 \| \| rno-miR-155-5p \| 2.088 \| 1.095 \| 1.622 \| 4.637 \| 5.950 \| 3.078 \| 0.941 \| 6.156 \| 6.556 \| 5.538 \| 5.811 \| 7.408 \| 6.294 \| 0.730 \| 0.012 \| 2.045 \| 2.045 \| 4.125 \| \| rno-miR-200b-3p \| 2.117 \| 0.882 \| 1.049 \| 1.740 \| 3.295 \| 1.817 \| 0.433 \| 3.311 \| 4.323 \| 2.137 \| 3.304 \| 5.416 \| 3.698 \| 1.233 \| 0.028 \| 2.036 \| 2.036 \| 4.100 \| \| rno-miR-30e-3p \| 1.407 \| 1.001 \| 1.099 \| 7.542 \| 8.062 \| 3.822 \| 1.628 \| 7.494 \| 7.832 \| 7.871 \| 7.388 \| 8.056 \| 7.728 \| 0.278 \| 0.044 \| 2.022 \| 2.022 \| 4.061 \| \| rno-miR-328b-3p \| 1.591 \| 1.202 \| 1.227 \| 1.548 \| 3.430 \| 1.800 \| 0.415 \| 3.638 \| 4.891 \| 3.139 \| 2.825 \| 2.614 \| 3.421 \| 0.908 \| 0.023 \| 1.901 \| 1.901 \| 3.735 \| \| rno-miR-505-3p \| 2.067 \| 1.021 \| 1.113 \| 1.659 \| 3.374 \| 1.847 \| 0.426 \| 3.858 \| 2.346 \| 2.793 \| 3.363 \| 4.895 \| 3.451 \| 0.989 \| 0.031 \| 1.869 \| 1.869 \| 3.652 \| \| rno-miR-382-5p \| 2.998 \| 0.887 \| 2.511 \| 1.756 \| 3.723 \| 2.375 \| 0.491 \| 4.688 \| 4.899 \| 2.791 \| 3.118 \| 5.508 \| 4.201 \| 1.183 \| 0.035 \| 1.769 \| 1.769 \| 3.407 \| \| rno-miR-499-5p \| 4.989 \| 1.279 \| 1.242 \| 7.231 \| 8.350 \| 4.618 \| 1.474 \| 8.269 \| 7.561 \| 7.755 \| 7.942 \| 8.634 \| 8.032 \| 0.426 \| 0.051 \| 1.739 \| 1.739 \| 3.339 \| \| rno-miR-872-5p \| 1.191 \| 0.994 \| 0.725 \| 0.900 \| 1.262 \| 1.014 \| 0.097 \| 1.418 \| 1.644 \| 1.661 \| 1.360 \| 2.554 \| 1.727 \| 0.481 \| 0.017 \| 1.703 \| 1.703 \| 3.256 \| \| rno-miR-3068-5p \| 2.930 \| 1.721 \| 1.565 \| 4.759 \| 6.075 \| 3.410 \| 0.878 \| 6.945 \| 4.692 \| 5.643 \| 5.288 \| 5.983 \| 5.710 \| 0.839 \| 0.043 \| 1.675 \| 1.675 \| 3.193 \| \| rno-miR-187-3p \| 2.605 \| 1.785 \| 1.694 \| 4.082 \| 2.772 \| 2.588 \| 0.431 \| 4.605 \| 3.219 \| 4.244 \| 3.849 \| 4.971 \| 4.178 \| 0.679 \| 0.017 \| 1.614 \| 1.614 \| 3.062 \| \| rno-miR-217-5p \| 1.640 \| 1.522 \| 1.188 \| 1.749 \| 1.393 \| 1.498 \| 0.098 \| 1.938 \| 1.984 \| 2.509 \| 1.847 \| 3.583 \| 2.372 \| 0.724 \| 0.033 \| 1.583 \| 1.583 \| 2.996 \| \| rno-miR-758-5p \| 0.709 \| 1.224 \| 1.649 \| 2.040 \| 1.840 \| 1.493 \| 0.238 \| 1.635 \| 2.763 \| 2.779 \| 2.525 \| 2.102 \| 2.361 \| 0.489 \| 0.028 \| 1.582 \| 1.582 \| 2.994 \| \| rno-miR-92b-3p \| 2.029 \| 1.875 \| 1.854 \| 3.377 \| 4.104 \| 2.648 \| 0.462 \| 4.749 \| 3.738 \| 3.267 \| 3.950 \| 4.911 \| 4.123 \| 0.693 \| 0.029 \| 1.557 \| 1.557 \| 2.943 \| \| rno-miR-3102 \| 1.850 \| 1.115 \| 1.441 \| 1.484 \| 1.289 \| 1.436 \| 0.122 \| 2.645 \| 2.658 \| 1.547 \| 1.677 \| 2.610 \| 2.227 \| 0.564 \| 0.022 \| 1.551 \| 1.551 \| 2.931 \| \| rno-miR-362-3p \| 1.029 \| 1.003 \| 1.057 \| 1.014 \| 1.871 \| 1.195 \| 0.169 \| 2.564 \| 1.267 \| 1.882 \| 1.649 \| 1.837 \| 1.840 \| 0.472 \| 0.044 \| 1.540 \| 1.540 \| 2.908 \| \| rno-miR-341 \| 1.168 \| 0.963 \| 1.319 \| 2.132 \| 2.338 \| 1.584 \| 0.274 \| 3.027 \| 1.808 \| 2.461 \| 2.485 \| 2.340 \| 2.424 \| 0.434 \| 0.037 \| 1.531 \| 1.531 \| 2.889 \| \| rno-mir-383 \| 1.771 \| 1.140 \| 1.242 \| 1.876 \| 1.426 \| 1.491 \| 0.144 \| 2.480 \| 2.439 \| 1.985 \| 2.196 \| 2.214 \| 2.263 \| 0.201 \| 0.002 \| 1.518 \| 1.518 \| 2.864 \| \| rno-miR-702-5p \| 2.797 \| 2.497 \| 1.706 \| 4.604 \| 4.556 \| 3.232 \| 0.578 \| 4.781 \| 5.213 \| 5.359 \| 4.618 \| 4.552 \| 4.905 \| 0.362 \| 0.024 \| 1.518 \| 1.518 \| 2.863 \| \| rno-miR-500-5p \| 1.030 \| 1.098 \| 1.290 \| 1.003 \| 1.114 \| 1.107 \| 0.050 \| 1.958 \| 1.480 \| 1.443 \| 1.267 \| 2.088 \| 1.647 \| 0.355 \| 0.012 \| 1.488 \| 1.488 \| 2.805 \| \| rno-miR-494-3p \| 3.121 \| 2.629 \| 3.830 \| 4.884 \| 4.948 \| 3.882 \| 0.463 \| 6.178 \| 5.345 \| 5.491 \| 5.477 \| 6.124 \| 5.723 \| 0.395 \| 0.006 \| 1.474 \| 1.474 \| 2.778 \| \| rno-miR-411-5p \| 1.102 \| 0.898 \| 1.293 \| 0.895 \| 1.003 \| 1.038 \| 0.074 \| 1.665 \| 1.219 \| 1.288 \| 1.253 \| 2.167 \| 1.519 \| 0.405 \| 0.040 \| 1.462 \| 1.462 \| 2.756 \| \| rno-miR-379-5p \| 4.438 \| 2.486 \| 3.780 \| 4.276 \| 3.483 \| 3.693 \| 0.347 \| 5.201 \| 5.570 \| 4.693 \| 4.669 \| 6.585 \| 5.344 \| 0.789 \| 0.010 \| 1.447 \| 1.447 \| 2.727 \| \| rno-let-7a-2-3p \| 0.828 \| 0.782 \| 1.122 \| 0.737 \| 1.118 \| 0.918 \| 0.084 \| 1.809 \| 0.995 \| 1.423 \| 1.072 \| 1.324 \| 1.325 \| 0.323 \| 0.041 \| 1.444 \| 1.444 \| 2.720 \| \| rno-miR-483-5p \| 4.671 \| 3.464 \| 2.154 \| 3.557 \| 2.722 \| 3.314 \| 0.425 \| 4.490 \| 4.961 \| 4.520 \| 4.973 \| 4.752 \| 4.739 \| 0.231 \| 0.012 \| 1.430 \| 1.430 \| 2.695 \| \| rno-miR-664-1-5p \| 4.177 \| 3.871 \| 2.893 \| 2.093 \| 4.589 \| 3.525 \| 0.454 \| 4.899 \| 5.337 \| 4.586 \| 4.160 \| 5.979 \| 4.992 \| 0.700 \| 0.029 \| 1.416 \| 1.416 \| 2.669 \| \| rno-miR-30c-5p \| 8.165 \| 6.912 \| 6.085 \| 13.374 \| 13.751 \| 9.657 \| 1.629 \| 13.714 \| 13.189 \| 13.691 \| 13.659 \| 13.791 \| 13.609 \| 0.239 \| 0.042 \| 1.409 \| 1.409 \| 2.656 \| \| rno-miR-742-5p \| 0.856 \| 0.831 \| 0.760 \| 0.849 \| 1.118 \| 0.883 \| 0.061 \| 1.263 \| 1.162 \| 1.333 \| 1.341 \| 1.100 \| 1.240 \| 0.106 \| 0.002 \| 1.404 \| 1.404 \| 2.647 \| \| rno-miR-30a-3p \| 6.594 \| 5.522 \| 4.082 \| 9.850 \| 10.144 \| 7.238 \| 1.196 \| 9.918 \| 10.288 \| 10.245 \| 10.041 \| 10.213 \| 10.141 \| 0.156 \| 0.042 \| 1.401 \| 1.401 \| 2.641 \| \| rno-mir-3085 \| 1.262 \| 1.214 \| 1.242 \| 0.968 \| 1.465 \| 1.230 \| 0.079 \| 2.268 \| 1.460 \| 1.646 \| 1.510 \| 1.640 \| 1.705 \| 0.325 \| 0.021 \| 1.386 \| 1.386 \| 2.613 \| \| rno-miR-182 \| 2.222 \| 2.833 \| 1.758 \| 1.918 \| 2.675 \| 2.281 \| 0.208 \| 3.989 \| 2.505 \| 3.481 \| 3.012 \| 2.545 \| 3.107 \| 0.633 \| 0.047 \| 1.362 \| 1.362 \| 2.570 \| \| rno-miR-196c-5p \| 1.140 \| 0.783 \| 1.238 \| 1.551 \| 1.079 \| 1.158 \| 0.124 \| 1.405 \| 1.254 \| 1.759 \| 1.813 \| 1.569 \| 1.560 \| 0.235 \| 0.039 \| 1.347 \| 1.347 \| 2.543 \| \| rno-miR-200c-5p \| 1.051 \| 0.814 \| 0.698 \| 1.037 \| 1.036 \| 0.927 \| 0.072 \| 1.306 \| 1.376 \| 1.132 \| 1.215 \| 1.172 \| 1.240 \| 0.099 \| 0.006 \| 1.337 \| 1.337 \| 2.527 \| \| rno-mir-877 \| 0.982 \| 0.690 \| 0.769 \| 0.819 \| 0.792 \| 0.810 \| 0.048 \| 1.213 \| 0.804 \| 1.245 \| 1.146 \| 1.008 \| 1.083 \| 0.180 \| 0.020 \| 1.337 \| 1.337 \| 2.525 \| \| rno-mir-3556b \| 0.789 \| 0.935 \| 0.977 \| 0.776 \| 1.338 \| 0.963 \| 0.102 \| 1.520 \| 1.305 \| 1.188 \| 1.172 \| 1.225 \| 1.282 \| 0.143 \| 0.029 \| 1.331 \| 1.331 \| 2.516 \| \| rno-mir-1306 \| 1.297 \| 1.017 \| 0.825 \| 0.929 \| 1.160 \| 1.046 \| 0.083 \| 1.413 \| 1.487 \| 1.365 \| 1.416 \| 1.203 \| 1.376 \| 0.107 \| 0.009 \| 1.316 \| 1.316 \| 2.490 \| \| rno-miR-352 \| 6.179 \| 5.576 \| 4.495 \| 6.660 \| 7.909 \| 6.164 \| 0.567 \| 8.078 \| 8.865 \| 7.523 \| 7.390 \| 8.663 \| 8.104 \| 0.660 \| 0.016 \| 1.315 \| 1.315 \| 2.488 \| \| rno-mir-188 \| 1.353 \| 1.113 \| 0.850 \| 1.159 \| 0.819 \| 1.059 \| 0.100 \| 1.677 \| 1.376 \| 1.310 \| 1.294 \| 1.301 \| 1.392 \| 0.163 \| 0.028 \| 1.314 \| 1.314 \| 2.487 \| \| rno-miR-6318 \| 1.412 \| 1.434 \| 1.374 \| 1.529 \| 1.677 \| 1.485 \| 0.054 \| 2.360 \| 1.658 \| 1.606 \| 2.344 \| 1.773 \| 1.948 \| 0.374 \| 0.030 \| 1.312 \| 1.312 \| 2.483 \| \| rno-miR-448-3p \| 1.029 \| 1.003 \| 1.181 \| 0.899 \| 0.788 \| 0.980 \| 0.066 \| 1.458 \| 1.176 \| 1.179 \| 1.469 \| 1.018 \| 1.260 \| 0.197 \| 0.034 \| 1.285 \| 1.285 \| 2.437 \| \| rno-miR-298-5p \| 5.098 \| 6.143 \| 5.271 \| 3.354 \| 4.712 \| 4.916 \| 0.455 \| 6.551 \| 6.472 \| 6.119 \| 5.628 \| 6.579 \| 6.270 \| 0.403 \| 0.024 \| 1.275 \| 1.275 \| 2.421 \| \| rno-miR-130a-5p \| 0.745 \| 0.678 \| 0.697 \| 0.574 \| 0.589 \| 0.656 \| 0.033 \| 0.924 \| 0.656 \| 0.809 \| 0.820 \| 0.971 \| 0.836 \| 0.122 \| 0.022 \| 1.274 \| 1.274 \| 2.418 \| \| rno-miR-146b-5p \| 5.596 \| 4.544 \| 6.691 \| 7.842 \| 8.001 \| 6.535 \| 0.661 \| 8.283 \| 8.081 \| 8.262 \| 8.429 \| 8.446 \| 8.300 \| 0.148 \| 0.029 \| 1.270 \| 1.270 \| 2.412 \| \| rno-miR-223-5p \| 0.852 \| 0.894 \| 0.837 \| 1.003 \| 0.709 \| 0.859 \| 0.047 \| 1.279 \| 0.915 \| 0.968 \| 1.139 \| 1.152 \| 1.090 \| 0.148 \| 0.022 \| 1.269 \| 1.269 \| 2.410 \| \| rno-mir-671 \| 0.913 \| 1.041 \| 0.760 \| 0.853 \| 1.016 \| 0.916 \| 0.052 \| 1.009 \| 0.933 \| 1.229 \| 1.253 \| 1.372 \| 1.159 \| 0.182 \| 0.036 \| 1.265 \| 1.265 \| 2.403 \| \| rno-miR-27b-5p \| 4.757 \| 4.801 \| 3.854 \| 4.928 \| 5.969 \| 4.862 \| 0.336 \| 6.428 \| 6.213 \| 5.666 \| 5.759 \| 6.559 \| 6.125 \| 0.398 \| 0.011 \| 1.260 \| 1.260 \| 2.395 \| \| rno-mir-6324 \| 1.143 \| 0.939 \| 1.391 \| 1.003 \| 1.133 \| 1.122 \| 0.078 \| 1.570 \| 1.324 \| 1.284 \| 1.535 \| 1.339 \| 1.411 \| 0.132 \| 0.018 \| 1.257 \| 1.257 \| 2.390 \| \| rno-mir-666 \| 1.152 \| 0.967 \| 1.227 \| 1.092 \| 1.109 \| 1.109 \| 0.043 \| 1.234 \| 1.182 \| 1.604 \| 1.669 \| 1.257 \| 1.389 \| 0.229 \| 0.036 \| 1.252 \| 1.252 \| 2.382 \| \| rno-miR-224-5p \| 6.766 \| 4.363 \| 5.613 \| 5.811 \| 6.195 \| 5.749 \| 0.399 \| 7.205 \| 7.046 \| 6.911 \| 7.092 \| 7.580 \| 7.167 \| 0.254 \| 0.009 \| 1.247 \| 1.247 \| 2.373 \| \| rno-mir-7b \| 0.947 \| 1.139 \| 0.955 \| 1.152 \| 1.341 \| 1.107 \| 0.073 \| 1.543 \| 1.490 \| 1.398 \| 1.257 \| 1.209 \| 1.379 \| 0.144 \| 0.023 \| 1.246 \| 1.246 \| 2.373 \| \| rno-miR-216b-5p \| 1.126 \| 1.570 \| 1.185 \| 1.318 \| 1.485 \| 1.337 \| 0.085 \| 1.638 \| 1.664 \| 1.389 \| 1.545 \| 2.088 \| 1.665 \| 0.260 \| 0.052 \| 1.246 \| 1.246 \| 2.371 \| \| rno-mir-344i \| 1.026 \| 1.189 \| 1.156 \| 1.326 \| 1.135 \| 1.167 \| 0.048 \| 1.413 \| 1.448 \| 1.291 \| 1.791 \| 1.272 \| 1.443 \| 0.209 \| 0.030 \| 1.237 \| 1.237 \| 2.357 \| \| rno-mir-876 \| 0.845 \| 1.093 \| 1.171 \| 1.086 \| 0.851 \| 1.009 \| 0.067 \| 1.245 \| 1.078 \| 1.177 \| 1.392 \| 1.305 \| 1.239 \| 0.120 \| 0.029 \| 1.228 \| 1.228 \| 2.342 \| \| rno-mir-504 \| 1.462 \| 1.161 \| 1.446 \| 1.616 \| 1.557 \| 1.449 \| 0.078 \| 1.430 \| 1.785 \| 1.949 \| 2.003 \| 1.673 \| 1.768 \| 0.230 \| 0.039 \| 1.221 \| 1.221 \| 2.330 \| \| rno-miR-25-3p \| 7.382 \| 6.477 \| 5.227 \| 8.088 \| 8.842 \| 7.203 \| 0.630 \| 8.595 \| 8.482 \| 8.769 \| 8.513 \| 9.344 \| 8.740 \| 0.355 \| 0.045 \| 1.213 \| 1.213 \| 2.319 \| \| rno-mir-598 \| 1.380 \| 1.356 \| 1.137 \| 1.380 \| 1.118 \| 1.274 \| 0.060 \| 1.763 \| 1.515 \| 1.674 \| 1.380 \| 1.284 \| 1.523 \| 0.199 \| 0.049 \| 1.196 \| 1.196 \| 2.290 \| \| rno-mir-325 \| 0.999 \| 1.003 \| 1.117 \| 0.822 \| 0.935 \| 0.975 \| 0.048 \| 1.116 \| 1.008 \| 1.060 \| 1.257 \| 1.315 \| 1.151 \| 0.130 \| 0.048 \| 1.180 \| 1.180 \| 2.266 \| \| rno-miR-205 \| 1.051 \| 1.137 \| 1.257 \| 1.088 \| 1.038 \| 1.114 \| 0.040 \| 1.237 \| 1.162 \| 1.177 \| 1.500 \| 1.486 \| 1.312 \| 0.167 \| 0.047 \| 1.178 \| 1.178 \| 2.262 \| \| rno-miR-28-3p \| 7.826 \| 7.163 \| 6.773 \| 8.400 \| 8.556 \| 7.744 \| 0.345 \| 8.948 \| 9.283 \| 9.081 \| 8.793 \| 9.114 \| 9.044 \| 0.184 \| 0.006 \| 1.168 \| 1.168 \| 2.247 \| \| rno-miR-539-5p \| 1.629 \| 1.272 \| 1.404 \| 1.395 \| 1.304 \| 1.401 \| 0.062 \| 1.512 \| 1.634 \| 1.858 \| 1.529 \| 1.629 \| 1.632 \| 0.138 \| 0.030 \| 1.165 \| 1.165 \| 2.243 \| \| rno-miR-10a-5p \| 6.678 \| 5.321 \| 5.482 \| 6.074 \| 6.822 \| 6.075 \| 0.303 \| 6.812 \| 7.151 \| 7.304 \| 6.866 \| 7.239 \| 7.074 \| 0.222 \| 0.014 \| 1.164 \| 1.164 \| 2.241 \| \| rno-miR-673-5p \| 1.229 \| 1.319 \| 1.182 \| 1.091 \| 0.993 \| 1.163 \| 0.056 \| 1.250 \| 1.316 \| 1.308 \| 1.409 \| 1.436 \| 1.344 \| 0.077 \| 0.025 \| 1.156 \| 1.156 \| 2.228 \| \| rno-miR-181b-5p \| 9.360 \| 9.349 \| 8.179 \| 10.873 \| 10.714 \| 9.695 \| 0.498 \| 11.084 \| 11.411 \| 11.287 \| 11.050 \| 11.010 \| 11.168 \| 0.173 \| 0.019 \| 1.152 \| 1.152 \| 2.222 \| \| rno-mir-26a \| 2.703 \| 2.209 \| 2.676 \| 2.457 \| 2.856 \| 2.580 \| 0.113 \| 2.719 \| 2.929 \| 2.958 \| 2.912 \| 3.006 \| 2.905 \| 0.110 \| 0.030 \| 1.126 \| 1.126 \| 2.182 \| \| rno-miR-6216 \| 10.314 \| 10.931 \| 10.589 \| 12.680 \| 12.661 \| 11.435 \| 0.514 \| 13.025 \| 13.006 \| 12.480 \| 12.499 \| 12.965 \| 12.795 \| 0.280 \| 0.033 \| 1.119 \| 1.119 \| 2.172 \| \| rno-miR-19a-5p \| 0.913 \| 0.978 \| 0.861 \| 1.072 \| 0.863 \| 0.937 \| 0.040 \| 0.968 \| 1.069 \| 1.067 \| 1.038 \| 1.094 \| 1.047 \| 0.049 \| 0.042 \| 1.117 \| 1.117 \| 2.169 \| \| rno-miR-20a-5p \| 9.551 \| 9.071 \| 9.213 \| 10.614 \| 10.815 \| 9.853 \| 0.362 \| 11.142 \| 10.499 \| 11.122 \| 10.897 \| 10.807 \| 10.893 \| 0.263 \| 0.026 \| 1.106 \| 1.106 \| 2.152 \| \| rno-miR-92a-3p \| 10.319 \| 10.171 \| 9.719 \| 10.565 \| 11.241 \| 10.403 \| 0.251 \| 11.366 \| 11.526 \| 11.309 \| 11.071 \| 11.730 \| 11.400 \| 0.246 \| 0.007 \| 1.096 \| 1.096 \| 2.137 \| \| rno-miR-29b-2-5p \| 7.137 \| 6.958 \| 6.339 \| 6.899 \| 7.141 \| 6.895 \| 0.147 \| 7.788 \| 7.143 \| 7.614 \| 7.181 \| 7.819 \| 7.509 \| 0.327 \| 0.018 \| 1.089 \| 1.089 \| 2.127 \| \| rno-miR-1-3p \| 11.523 \| 10.778 \| 11.276 \| 12.309 \| 12.925 \| 11.762 \| 0.382 \| 12.460 \| 13.126 \| 12.348 \| 12.563 \| 13.017 \| 12.703 \| 0.347 \| 0.052 \| 1.080 \| 1.080 \| 2.114 \| \| rno-miR-222-3p \| 9.382 \| 9.204 \| 9.572 \| 9.559 \| 9.284 \| 9.400 \| 0.073 \| 9.991 \| 10.021 \| 10.041 \| 10.034 \| 10.182 \| 10.054 \| 0.074 \| 0.000 \| 1.070 \| 1.070 \| 2.099 \| \| rno-miR-221-3p \| 9.598 \| 8.920 \| 8.811 \| 9.262 \| 9.444 \| 9.207 \| 0.150 \| 9.835 \| 9.664 \| 10.010 \| 9.648 \| 9.925 \| 9.817 \| 0.159 \| 0.006 \| 1.066 \| 1.066 \| 2.094 \| \| rno-mir-361 \| 8.636 \| 9.136 \| 8.823 \| 8.886 \| 8.903 \| 8.877 \| 0.080 \| 9.447 \| 9.475 \| 9.576 \| 9.230 \| 9.505 \| 9.447 \| 0.130 \| 0.000 \| 1.064 \| 1.064 \| 2.091 \| \| rno-miR-361-5p \| 11.440 \| 10.973 \| 11.426 \| 11.161 \| 11.140 \| 11.228 \| 0.090 \| 11.797 \| 11.955 \| 11.695 \| 11.563 \| 11.914 \| 11.785 \| 0.161 \| 0.001 \| 1.050 \| 1.050 \| 2.070 \| \| rno-mir-344a-1 \| 1.899 \| 1.966 \| 1.930 \| 1.946 \| 1.993 \| 1.947 \| 0.016 \| 2.034 \| 1.944 \| 2.081 \| 2.055 \| 2.048 \| 2.032 \| 0.053 \| 0.017 \| 1.044 \| 1.044 \| 2.062 \| \| rno-mir-344a-2 \| 1.899 \| 1.966 \| 1.930 \| 1.946 \| 1.993 \| 1.947 \| 0.016 \| 2.034 \| 1.944 \| 2.081 \| 2.055 \| 2.048 \| 2.032 \| 0.053 \| 0.017 \| 1.044 \| 1.044 \| 2.062 \| \| rno-miR-125a-5p \| 12.838 \| 12.818 \| 12.815 \| 12.900 \| 13.108 \| 12.896 \| 0.055 \| 12.860 \| 13.557 \| 13.258 \| 13.159 \| 13.508 \| 13.268 \| 0.283 \| 0.027 \| 1.029 \| 1.029 \| 2.040 \| \| rno-miR-143-3p \| 14.256 \| 14.029 \| 14.071 \| 13.958 \| 13.971 \| 14.057 \| 0.054 \| 13.877 \| 13.875 \| 14.013 \| 13.851 \| 13.779 \| 13.879 \| 0.085 \| 0.027 \| 0.987 \| -1.013 \| 0.496 \| \| rno-miR-139-5p \| 11.014 \| 10.975 \| 10.734 \| 10.645 \| 10.523 \| 10.778 \| 0.095 \| 10.523 \| 10.218 \| 10.645 \| 10.356 \| 10.638 \| 10.476 \| 0.186 \| 0.043 \| 0.972 \| -1.029 \| 0.490 \| \| rno-miR-24-2-5p \| 10.996 \| 10.958 \| 10.614 \| 10.302 \| 10.413 \| 10.657 \| 0.140 \| 10.414 \| 10.154 \| 10.308 \| 10.066 \| 10.199 \| 10.228 \| 0.136 \| 0.023 \| 0.960 \| -1.042 \| 0.486 \| \| rno-miR-133a-5p \| 10.642 \| 10.519 \| 10.061 \| 10.108 \| 10.536 \| 10.373 \| 0.120 \| 9.853 \| 9.626 \| 10.157 \| 9.861 \| 9.193 \| 9.738 \| 0.358 \| 0.013 \| 0.939 \| -1.065 \| 0.478 \| \| rno-miR-210-3p \| 11.358 \| 11.764 \| 10.880 \| 10.550 \| 9.984 \| 10.907 \| 0.310 \| 10.488 \| 9.354 \| 10.412 \| 10.080 \| 9.486 \| 9.964 \| 0.522 \| 0.041 \| 0.914 \| -1.095 \| 0.468 \| \| rno-miR-497-5p \| 10.018 \| 9.933 \| 9.547 \| 9.627 \| 9.140 \| 9.653 \| 0.156 \| 9.235 \| 7.890 \| 9.142 \| 9.031 \| 8.780 \| 8.816 \| 0.545 \| 0.020 \| 0.913 \| -1.095 \| 0.468 \| \| rno-miR-345-5p \| 9.219 \| 9.044 \| 8.505 \| 8.225 \| 8.604 \| 8.719 \| 0.181 \| 8.463 \| 6.429 \| 8.064 \| 7.686 \| 8.161 \| 7.761 \| 0.794 \| 0.043 \| 0.890 \| -1.124 \| 0.459 \| \| rno-miR-345-3p \| 9.004 \| 8.945 \| 8.449 \| 7.590 \| 7.780 \| 8.354 \| 0.291 \| 7.640 \| 7.367 \| 7.521 \| 7.116 \| 7.216 \| 7.372 \| 0.214 \| 0.013 \| 0.882 \| -1.133 \| 0.456 \| \| rno-miR-3473 \| 10.507 \| 10.535 \| 9.708 \| 9.660 \| 9.670 \| 10.016 \| 0.206 \| 9.122 \| 8.491 \| 8.152 \| 8.756 \| 9.426 \| 8.789 \| 0.503 \| 0.004 \| 0.878 \| -1.140 \| 0.454 \| \| rno-mir-3544 \| 1.137 \| 1.190 \| 1.167 \| 1.274 \| 1.337 \| 1.221 \| 0.037 \| 1.155 \| 1.086 \| 1.130 \| 1.003 \| 0.950 \| 1.065 \| 0.086 \| 0.019 \| 0.872 \| -1.147 \| 0.452 \| \| rno-miR-3583-3p \| 0.888 \| 0.978 \| 0.876 \| 0.869 \| 0.906 \| 0.903 \| 0.020 \| 0.938 \| 0.782 \| 0.666 \| 0.798 \| 0.742 \| 0.785 \| 0.100 \| 0.041 \| 0.869 \| -1.150 \| 0.451 \| \| rno-miR-129-1-3p \| 1.210 \| 0.941 \| 1.278 \| 1.261 \| 1.317 \| 1.201 \| 0.067 \| 1.100 \| 1.070 \| 0.948 \| 1.028 \| 1.018 \| 1.033 \| 0.057 \| 0.048 \| 0.860 \| -1.163 \| 0.446 \| \| rno-mir-3571 \| 1.297 \| 1.082 \| 1.272 \| 1.172 \| 0.982 \| 1.161 \| 0.059 \| 1.044 \| 1.067 \| 0.931 \| 0.977 \| 0.949 \| 0.993 \| 0.059 \| 0.032 \| 0.856 \| -1.169 \| 0.445 \| \| rno-mir-509 \| 1.151 \| 0.890 \| 0.887 \| 0.868 \| 0.959 \| 0.951 \| 0.052 \| 0.780 \| 0.794 \| 0.857 \| 0.852 \| 0.758 \| 0.808 \| 0.044 \| 0.034 \| 0.850 \| -1.177 \| 0.442 \| \| rno-miR-146b-3p \| 1.077 \| 1.327 \| 1.106 \| 1.178 \| 1.096 \| 1.157 \| 0.046 \| 1.163 \| 0.843 \| 0.865 \| 1.003 \| 0.907 \| 0.956 \| 0.131 \| 0.027 \| 0.827 \| -1.210 \| 0.432 \| \| rno-miR-127-5p \| 1.785 \| 2.035 \| 2.416 \| 2.176 \| 1.969 \| 2.076 \| 0.106 \| 1.788 \| 1.888 \| 1.334 \| 1.524 \| 1.909 \| 1.689 \| 0.251 \| 0.036 \| 0.813 \| -1.229 \| 0.427 \| \| rno-mir-543 \| 1.096 \| 1.082 \| 1.114 \| 0.880 \| 1.136 \| 1.061 \| 0.046 \| 0.854 \| 0.876 \| 0.852 \| 0.779 \| 0.911 \| 0.855 \| 0.048 \| 0.004 \| 0.805 \| -1.242 \| 0.423 \| \| rno-miR-935 \| 1.121 \| 1.184 \| 1.150 \| 1.326 \| 0.976 \| 1.151 \| 0.056 \| 0.845 \| 0.957 \| 0.804 \| 0.865 \| 1.150 \| 0.924 \| 0.138 \| 0.026 \| 0.803 \| -1.246 \| 0.422 \| \| rno-mir-1249 \| 1.233 \| 1.345 \| 1.242 \| 1.288 \| 1.288 \| 1.279 \| 0.020 \| 0.889 \| 1.278 \| 0.965 \| 1.087 \| 0.863 \| 1.017 \| 0.170 \| 0.010 \| 0.795 \| -1.258 \| 0.418 \| \| rno-mir-345 \| 1.029 \| 1.140 \| 1.291 \| 1.151 \| 1.487 \| 1.219 \| 0.079 \| 1.040 \| 0.919 \| 1.213 \| 0.854 \| 0.809 \| 0.967 \| 0.163 \| 0.047 \| 0.793 \| -1.261 \| 0.417 \| \| rno-miR-883-3p \| 1.036 \| 1.150 \| 1.103 \| 0.904 \| 0.906 \| 1.020 \| 0.050 \| 0.768 \| 0.906 \| 0.666 \| 0.661 \| 1.038 \| 0.808 \| 0.163 \| 0.044 \| 0.792 \| -1.262 \| 0.417 \| \| rno-mir-219a-2 \| 1.347 \| 1.161 \| 1.103 \| 0.979 \| 0.953 \| 1.109 \| 0.071 \| 0.960 \| 0.874 \| 0.934 \| 0.873 \| 0.740 \| 0.876 \| 0.085 \| 0.020 \| 0.790 \| -1.265 \| 0.416 \| \| rno-miR-21-3p \| 1.630 \| 1.107 \| 1.553 \| 1.484 \| 1.456 \| 1.446 \| 0.090 \| 1.180 \| 1.187 \| 1.035 \| 1.187 \| 1.102 \| 1.138 \| 0.068 \| 0.012 \| 0.787 \| -1.271 \| 0.414 \| \| rno-miR-144-3p \| 1.640 \| 1.315 \| 1.408 \| 0.989 \| 1.197 \| 1.310 \| 0.108 \| 1.016 \| 0.977 \| 0.968 \| 1.021 \| 1.029 \| 1.002 \| 0.028 \| 0.022 \| 0.765 \| -1.307 \| 0.404 \| \| rno-miR-3559-5p \| 1.263 \| 1.260 \| 1.561 \| 1.123 \| 1.047 \| 1.251 \| 0.088 \| 1.082 \| 0.743 \| 0.905 \| 0.859 \| 1.064 \| 0.931 \| 0.143 \| 0.019 \| 0.744 \| -1.344 \| 0.394 \| \| rno-mir-181a-2 \| 1.029 \| 1.630 \| 0.957 \| 1.216 \| 1.187 \| 1.204 \| 0.117 \| 1.011 \| 0.765 \| 0.901 \| 0.727 \| 1.074 \| 0.896 \| 0.151 \| 0.052 \| 0.744 \| -1.344 \| 0.394 \| \| rno-miR-344i \| 1.297 \| 1.434 \| 1.474 \| 1.132 \| 0.900 \| 1.247 \| 0.105 \| 0.696 \| 0.974 \| 0.863 \| 1.148 \| 0.925 \| 0.921 \| 0.165 \| 0.035 \| 0.738 \| -1.354 \| 0.391 \| \| rno-miR-185-3p \| 1.364 \| 1.465 \| 0.843 \| 0.985 \| 0.960 \| 1.123 \| 0.122 \| 0.949 \| 0.674 \| 0.731 \| 0.923 \| 0.819 \| 0.819 \| 0.119 \| 0.052 \| 0.729 \| -1.372 \| 0.386 \| \| rno-miR-421-3p \| 1.143 \| 1.438 \| 1.314 \| 1.071 \| 1.120 \| 1.217 \| 0.069 \| 0.937 \| 0.900 \| 0.808 \| 0.938 \| 0.821 \| 0.881 \| 0.063 \| 0.002 \| 0.723 \| -1.382 \| 0.384 \| \| rno-mir-124-1 \| 1.542 \| 1.434 \| 1.364 \| 0.866 \| 1.091 \| 1.259 \| 0.123 \| 0.697 \| 1.020 \| 0.882 \| 1.091 \| 0.752 \| 0.888 \| 0.168 \| 0.033 \| 0.706 \| -1.417 \| 0.374 \| \| rno-miR-489-5p \| 1.143 \| 1.640 \| 1.257 \| 1.137 \| 1.066 \| 1.248 \| 0.102 \| 0.745 \| 0.861 \| 0.729 \| 0.892 \| 1.117 \| 0.869 \| 0.156 \| 0.015 \| 0.696 \| -1.437 \| 0.369 \| \| rno-mir-181b-1 \| 1.044 \| 1.369 \| 1.793 \| 1.664 \| 1.035 \| 1.381 \| 0.156 \| 0.967 \| 0.977 \| 0.887 \| 0.915 \| 1.043 \| 0.958 \| 0.061 \| 0.028 \| 0.693 \| -1.442 \| 0.368 \| \| rno-miR-764-3p \| 1.528 \| 1.132 \| 1.555 \| 1.551 \| 1.018 \| 1.357 \| 0.117 \| 0.687 \| 0.874 \| 0.664 \| 0.954 \| 1.106 \| 0.857 \| 0.186 \| 0.008 \| 0.632 \| -1.583 \| 0.334 \| |  |  |  |  |  |  |  |  |  |  |  |  |  |  |  |  |  |  |
| --- | --- | --- | --- | --- | --- | --- | --- | --- | --- | --- | --- | --- | --- | --- | --- | --- | --- | --- | --- | --- | --- | --- | --- | --- | --- | --- | --- | --- | --- | --- | --- | --- | --- | --- | --- | --- | --- | --- | --- | --- | --- | --- | --- | --- | --- | --- | --- | --- | --- | --- | --- | --- | --- | --- | --- | --- | --- | --- | --- | --- | --- | --- | --- | --- | --- | --- | --- | --- | --- | --- | --- | --- | --- | --- | --- | --- | --- | --- | --- | --- | --- | --- | --- | --- | --- | --- | --- | --- | --- | --- | --- | --- | --- | --- | --- | --- | --- | --- | --- | --- | --- | --- | --- | --- | --- | --- | --- | --- | --- | --- | --- | --- | --- | --- | --- | --- | --- | --- | --- | --- | --- | --- | --- | --- | --- | --- | --- | --- | --- | --- | --- | --- | --- | --- | --- | --- | --- | --- | --- | --- | --- | --- | --- | --- | --- | --- | --- | --- | --- | --- | --- | --- | --- | --- | --- | --- | --- | --- | --- | --- | --- | --- | --- | --- | --- | --- | --- | --- | --- | --- | --- | --- | --- | --- | --- | --- | --- | --- | --- | --- | --- | --- | --- | --- | --- | --- | --- | --- | --- | --- | --- | --- | --- | --- | --- | --- | --- | --- | --- | --- | --- | --- | --- | --- | --- | --- | --- | --- | --- | --- | --- | --- | --- | --- | --- | --- | --- | --- | --- | --- | --- | --- | --- | --- | --- | --- | --- | --- | --- | --- | --- | --- | --- | --- | --- | --- | --- | --- | --- | --- | --- | --- | --- | --- | --- | --- | --- | --- | --- | --- | --- | --- | --- | --- | --- | --- | --- | --- | --- | --- | --- | --- | --- | --- | --- | --- | --- | --- | --- | --- | --- | --- | --- | --- | --- | --- | --- | --- | --- | --- | --- | --- | --- | --- | --- | --- | --- | --- | --- | --- | --- | --- | --- | --- | --- | --- | --- | --- | --- | --- | --- | --- | --- | --- | --- | --- | --- | --- | --- | --- | --- | --- | --- | --- | --- | --- | --- | --- | --- | --- | --- | --- | --- | --- | --- | --- | --- | --- | --- | --- | --- | --- | --- | --- | --- | --- | --- | --- | --- | --- | --- | --- | --- | --- | --- | --- | --- | --- | --- | --- | --- | --- | --- | --- | --- | --- | --- | --- | --- | --- | --- | --- | --- | --- | --- | --- | --- | --- | --- | --- | --- | --- | --- | --- | --- | --- | --- | --- | --- | --- | --- | --- | --- | --- | --- | --- | --- | --- | --- | --- | --- | --- | --- | --- | --- | --- | --- | --- | --- | --- | --- | --- | --- | --- | --- | --- | --- | --- | --- | --- | --- | --- | --- | --- | --- | --- | --- | --- | --- | --- | --- | --- | --- | --- | --- | --- | --- | --- | --- | --- | --- | --- | --- | --- | --- | --- | --- | --- | --- | --- | --- | --- | --- | --- | --- | --- | --- | --- | --- | --- | --- | --- | --- | --- | --- | --- | --- | --- | --- | --- | --- | --- | --- | --- | --- | --- | --- | --- | --- | --- | --- | --- | --- | --- | --- | --- | --- | --- | --- | --- | --- | --- | --- | --- | --- | --- | --- | --- | --- | --- | --- | --- | --- | --- | --- | --- | --- | --- | --- | --- | --- | --- | --- | --- | --- | --- | --- | --- | --- | --- | --- | --- | --- | --- | --- | --- | --- | --- | --- | --- | --- | --- | --- | --- | --- | --- | --- | --- | --- | --- | --- | --- | --- | --- | --- | --- | --- | --- | --- | --- | --- | --- | --- | --- | --- | --- | --- | --- | --- | --- | --- | --- | --- | --- | --- | --- | --- | --- | --- | --- | --- | --- | --- | --- | --- | --- | --- | --- | --- | --- | --- | --- | --- | --- | --- | --- | --- | --- | --- | --- | --- | --- | --- | --- | --- | --- | --- | --- | --- | --- | --- | --- | --- | --- | --- | --- | --- | --- | --- | --- | --- | --- | --- | --- | --- | --- | --- | --- | --- | --- | --- | --- | --- | --- | --- | --- | --- | --- | --- | --- | --- | --- | --- | --- | --- | --- | --- | --- | --- | --- | --- | --- | --- | --- | --- | --- | --- | --- | --- | --- | --- | --- | --- | --- | --- | --- | --- | --- | --- | --- | --- | --- | --- | --- | --- | --- | --- | --- | --- | --- | --- | --- | --- | --- | --- | --- | --- | --- | --- | --- | --- | --- | --- | --- | --- | --- | --- | --- | --- | --- | --- | --- | --- | --- | --- | --- | --- | --- | --- | --- | --- | --- | --- | --- | --- | --- | --- | --- | --- | --- | --- | --- | --- | --- | --- | --- | --- | --- | --- | --- | --- | --- | --- | --- | --- | --- | --- | --- | --- | --- | --- | --- | --- | --- | --- | --- | --- | --- | --- | --- | --- | --- | --- | --- | --- | --- | --- | --- | --- | --- | --- | --- | --- | --- | --- | --- | --- | --- | --- | --- | --- | --- | --- | --- | --- | --- | --- | --- | --- | --- | --- | --- | --- | --- | --- | --- | --- | --- | --- | --- | --- | --- | --- | --- | --- | --- | --- | --- | --- | --- | --- | --- | --- | --- | --- | --- | --- | --- | --- | --- | --- | --- | --- | --- | --- | --- | --- | --- | --- | --- | --- | --- | --- | --- | --- | --- | --- | --- | --- | --- | --- | --- | --- | --- | --- | --- | --- | --- | --- | --- | --- | --- | --- | --- | --- | --- | --- | --- | --- | --- | --- | --- | --- | --- | --- | --- | --- | --- | --- | --- | --- | --- | --- | --- | --- | --- | --- | --- | --- | --- | --- | --- | --- | --- | --- | --- | --- | --- | --- | --- | --- | --- | --- | --- | --- | --- | --- | --- | --- | --- | --- | --- | --- | --- | --- | --- | --- | --- | --- | --- | --- | --- | --- | --- | --- | --- | --- | --- | --- | --- | --- | --- | --- | --- | --- | --- | --- | --- | --- | --- | --- | --- | --- | --- | --- | --- | --- | --- | --- | --- | --- | --- | --- | --- | --- | --- | --- | --- | --- | --- | --- | --- | --- | --- | --- | --- | --- | --- | --- | --- | --- | --- | --- | --- | --- | --- | --- | --- | --- | --- | --- | --- | --- | --- | --- | --- | --- | --- | --- | --- | --- | --- | --- | --- | --- | --- | --- | --- | --- | --- | --- | --- | --- | --- | --- | --- | --- | --- | --- | --- | --- | --- | --- | --- | --- | --- | --- | --- | --- | --- | --- | --- | --- | --- | --- | --- | --- | --- | --- | --- | --- | --- | --- | --- | --- | --- | --- | --- | --- | --- | --- | --- | --- | --- | --- | --- | --- | --- | --- | --- | --- | --- | --- | --- | --- | --- | --- | --- | --- | --- | --- | --- | --- | --- | --- | --- | --- | --- | --- | --- | --- | --- | --- | --- | --- | --- | --- | --- | --- | --- | --- | --- | --- | --- | --- | --- | --- | --- | --- | --- | --- | --- | --- | --- | --- | --- | --- | --- | --- | --- | --- | --- | --- | --- | --- | --- | --- | --- | --- | --- | --- | --- | --- | --- | --- | --- | --- | --- | --- | --- | --- | --- | --- | --- | --- | --- | --- | --- | --- | --- | --- | --- | --- | --- | --- | --- | --- | --- | --- | --- | --- | --- | --- | --- | --- | --- | --- | --- | --- | --- | --- | --- | --- | --- | --- | --- | --- | --- | --- | --- | --- | --- | --- | --- | --- | --- | --- | --- | --- | --- | --- | --- | --- | --- | --- | --- | --- | --- | --- | --- | --- | --- | --- | --- | --- | --- | --- | --- | --- | --- | --- | --- | --- | --- | --- | --- | --- | --- | --- | --- | --- | --- | --- | --- | --- | --- | --- | --- | --- | --- | --- | --- | --- | --- | --- | --- | --- | --- | --- | --- | --- | --- | --- | --- | --- | --- | --- | --- | --- | --- | --- | --- | --- | --- | --- | --- | --- | --- | --- | --- | --- | --- | --- | --- | --- | --- | --- | --- | --- | --- | --- | --- | --- | --- | --- | --- | --- | --- | --- | --- | --- | --- | --- | --- | --- | --- | --- | --- | --- | --- | --- | --- | --- | --- | --- | --- | --- | --- | --- | --- | --- | --- | --- | --- | --- | --- | --- | --- | --- | --- | --- | --- | --- | --- | --- | --- | --- | --- | --- | --- | --- | --- | --- | --- | --- | --- | --- | --- | --- | --- | --- | --- | --- | --- | --- | --- | --- | --- | --- | --- | --- | --- | --- | --- | --- | --- | --- | --- | --- | --- | --- | --- | --- | --- | --- | --- | --- | --- | --- | --- | --- | --- | --- | --- | --- | --- | --- | --- | --- | --- | --- | --- | --- | --- | --- | --- | --- | --- | --- | --- | --- | --- | --- | --- | --- | --- | --- | --- | --- | --- | --- | --- | --- | --- | --- | --- | --- | --- | --- | --- | --- | --- | --- | --- | --- | --- | --- | --- | --- | --- | --- | --- | --- | --- | --- | --- | --- | --- | --- | --- | --- | --- | --- | --- | --- | --- | --- | --- | --- | --- | --- | --- | --- | --- | --- | --- | --- | --- | --- | --- | --- | --- | --- | --- | --- | --- | --- | --- | --- | --- | --- | --- | --- | --- | --- | --- | --- | --- | --- | --- | --- | --- | --- | --- | --- | --- | --- | --- | --- | --- | --- | --- | --- | --- | --- | --- | --- | --- | --- | --- | --- | --- | --- | --- | --- | --- | --- | --- | --- | --- | --- | --- | --- | --- | --- | --- | --- | --- | --- | --- | --- | --- | --- | --- | --- | --- | --- | --- | --- | --- | --- | --- | --- | --- | --- | --- | --- | --- | --- | --- | --- | --- | --- | --- | --- | --- | --- | --- | --- | --- | --- | --- | --- | --- | --- | --- | --- | --- | --- | --- | --- | --- | --- | --- | --- | --- | --- | --- | --- | --- | --- | --- | --- | --- | --- | --- | --- | --- | --- | --- | --- | --- | --- | --- | --- | --- | --- | --- | --- | --- | --- | --- | --- | --- | --- | --- | --- | --- | --- | --- | --- | --- | --- | --- | --- | --- | --- | --- | --- | --- | --- | --- | --- | --- | --- | --- | --- | --- | --- | --- | --- | --- | --- | --- | --- | --- | --- | --- | --- | --- | --- | --- | --- | --- | --- | --- | --- | --- | --- | --- | --- | --- | --- | --- | --- | --- | --- | --- | --- | --- | --- | --- | --- | --- | --- | --- | --- | --- | --- | --- | --- | --- | --- | --- | --- | --- | --- | --- | --- | --- | --- | --- | --- | --- | --- | --- | --- | --- | --- | --- | --- | --- | --- | --- | --- | --- | --- | --- | --- | --- | --- | --- | --- | --- | --- | --- | --- | --- | --- | --- | --- | --- | --- | --- | --- | --- | --- | --- | --- | --- | --- | --- | --- | --- | --- | --- | --- | --- | --- | --- | --- | --- | --- | --- | --- | --- | --- | --- | --- | --- | --- | --- | --- | --- | --- | --- | --- | --- | --- | --- | --- | --- | --- | --- | --- | --- | --- | --- | --- | --- | --- | --- | --- | --- | --- | --- | --- | --- | --- | --- | --- | --- | --- | --- | --- | --- | --- | --- | --- | --- | --- | --- | --- | --- | --- | --- | --- | --- | --- | --- | --- | --- | --- | --- | --- | --- | --- | --- | --- | --- | --- | --- | --- | --- | --- | --- | --- | --- | --- | --- | --- | --- | --- | --- | --- | --- | --- | --- | --- | --- | --- | --- | --- | --- | --- | --- | --- | --- | --- | --- | --- | --- | --- | --- | --- | --- | --- | --- | --- | --- | --- | --- | --- | --- | --- | --- | --- | --- | --- | --- | --- | --- | --- | --- | --- | --- | --- | --- | --- | --- | --- | --- | --- | --- | --- | --- | --- | --- | --- | --- | --- | --- | --- | --- | --- | --- | --- | --- | --- | --- | --- | --- | --- | --- | --- | --- | --- | --- | --- | --- | --- | --- | --- | --- | --- | --- | --- | --- | --- | --- | --- | --- | --- | --- | --- | --- | --- | --- | --- | --- | --- | --- | --- | --- | --- | --- | --- | --- | --- | --- | --- | --- | --- | --- | --- | --- | --- | --- | --- | --- | --- | --- | --- | --- | --- | --- | --- | --- | --- | --- | --- | --- | --- | --- | --- | --- | --- | --- | --- | --- | --- | --- | --- | --- | --- | --- | --- | --- | --- | --- | --- | --- | --- | --- | --- | --- | --- | --- | --- | --- | --- | --- | --- | --- | --- | --- | --- | --- | --- | --- | --- | --- | --- | --- | --- | --- | --- | --- | --- | --- | --- | --- | --- | --- | --- | --- | --- | --- | --- | --- | --- | --- | --- | --- | --- | --- | --- | --- | --- | --- | --- | --- | --- | --- | --- | --- | --- | --- | --- | --- | --- | --- | --- | --- | --- | --- | --- | --- | --- | --- | --- | --- | --- | --- | --- | --- | --- | --- | --- | --- | --- | --- | --- | --- | --- | --- | --- | --- | --- | --- | --- | --- | --- | --- | --- | --- | --- | --- | --- | --- | --- | --- | --- | --- | --- | --- | --- | --- | --- | --- | --- | --- | --- | --- | --- | --- | --- | --- | --- | --- | --- | --- | --- | --- | --- | --- | --- | --- | --- | --- | --- | --- | --- | --- | --- | --- | --- | --- | --- | --- | --- | --- | --- | --- | --- | --- | --- | --- | --- | --- | --- | --- | --- | --- | --- | --- | --- | --- | --- | --- | --- | --- | --- | --- | --- | --- | --- | --- | --- | --- | --- | --- | --- | --- | --- | --- | --- | --- | --- | --- | --- | --- | --- | --- | --- | --- | --- | --- | --- | --- | --- | --- | --- | --- | --- | --- | --- | --- | --- | --- | --- | --- | --- | --- | --- | --- | --- | --- | --- | --- | --- | --- | --- | --- | --- | --- | --- | --- | --- | --- | --- | --- | --- | --- | --- | --- | --- | --- | --- | --- | --- | --- | --- | --- | --- | --- | --- | --- | --- | --- | --- | --- | --- | --- | --- | --- | --- | --- | --- | --- | --- | --- | --- | --- | --- | --- | --- | --- | --- | --- | --- | --- | --- | --- | --- | --- | --- | --- | --- | --- | --- | --- | --- | --- | --- | --- | --- | --- | --- | --- | --- | --- | --- | --- | --- | --- | --- | --- | --- | --- | --- | --- | --- | --- | --- | --- | --- | --- | --- | --- | --- | --- | --- | --- | --- | --- | --- | --- | --- | --- | --- | --- | --- | --- | --- | --- | --- | --- | --- | --- | --- | --- | --- | --- | --- | --- | --- | --- | --- | --- | --- | --- | --- | --- | --- | --- | --- | --- | --- | --- | --- | --- | --- | --- | --- | --- | --- | --- | --- | --- | --- | --- | --- | --- | --- | --- | --- | --- | --- | --- | --- | --- | --- | --- | --- | --- | --- | --- | --- | --- | --- |

| Supplemental Table 5. KEGG pathway enrichment analysis of pathways corresponding to differentially expressed miRNA*. | | | |
| --- | --- | --- | --- |
| **Name** | **miRNAs** | | **Target Genes** |
| p53 signaling pathway | | 31 | 42 |
| Pathways in cancer | | 36 | 234 |
| Colorectal cancer | | 30 | 44 |
| TGF-beta signaling pathway | | 32 | 52 |
| Chronic myeloid leukemia | | 31 | 51 |
| Glioma | | 31 | 44 |
| mTOR signaling pathway | | 31 | 43 |
| Focal adhesion | | 34 | 125 |
| Prostate cancer | | 31 | 55 |
| ECM-receptor interaction | | 29 | 48 |
| * Top 10 significantly enriched pathways appearing on both mirPath and miRNet. | | | |
